# Studies toward Providencin: The Furanyl-Cyclobutanol Segment

**DOI:** 10.1021/acs.orglett.3c00327

**Published:** 2023-02-27

**Authors:** Simon M. Spohr, Alois Fürstner

**Affiliations:** † Max-Planck-Institut für Kohlenforschung, D-45470 Mülheim/Ruhr, Germany

## Abstract

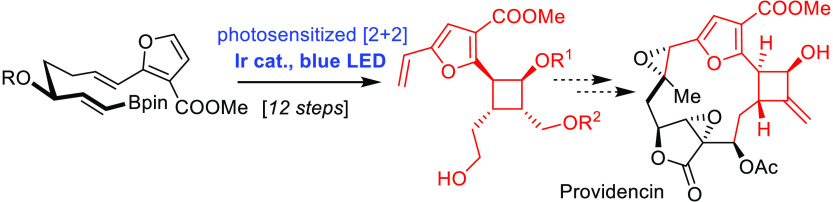

The furanocembranoid providencin remains an unconquered bastion, although the synthesis of 17-deoxyprovidencin—lacking a single −OH group—has been accomplished in the past. This paper describes a practical approach to a properly hydroxylated building block via an iridium-catalyzed photosensitized intramolecular [2 + 2] cycloaddition as the key step. While an attempt to convert this compound into providencin via RCAM failed, it might well be elaborated into the natural product by adopting the literature route.

Providencin (**1**) stands out as a deceptively challenging target among the large number of furanocembranoids and related diterpenes known in the literature.^1−3^ Actually, the total synthesis of (+)-**1** remains elusive, despite numerous creative studies directed toward this prominent marine natural product (Scheme 1).^4−7^ It is the massive ring strain that presents the biggest challenge; this notion is best appreciated if one tries—to no avail—to build a Dreiding model of the compound.^8^ The 7,8-*trans* epoxide (or the corresponding *E*-alkene precursor)^9,10^ is arguably the single most stiffening substructure, closely followed by the cyclobutyl alcohol *trans*-annulated to the macrocyclic frame.

**Scheme 1:**
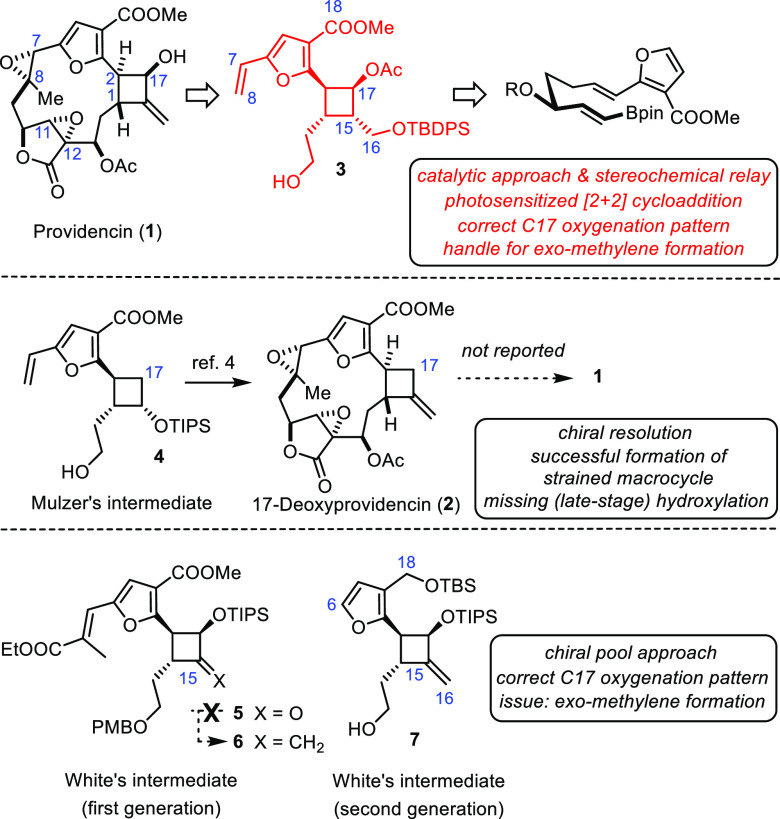
Structure of Providencin and Summary of Relevant Prior Art Concerning the Cyclobutylfuran Segment

A survey of the literature shows that this latter entity, itself, is nontrivial to make in adequately functionalized format. Thus, a heroic effort allowed the Mulzer group to advance compound **4** into non-natural 17-deoxyprovidencin (**2**) via macrocyclization by ring-closing metathesis (RCM), followed by photochemical *Z* → *E* isomerization of the trisubstituted Δ^7,8^-alkene thus formed, as the key steps;^4^ unfortunately, the missing hydroxy group on the four-membered ring precluded the conquest of **1** as the actual target. Although a selective late-stage hydroxylation at the C17 site is obviously challenging in view of the oxidation-prone furan ring, the authors pursued this risky strategy because of the lack of an adequately hydroxylated building block analogous to **4**.^11^ Equally noteworthy is an observation reported by White and Jana that all attempts to build the *exo*-methylene group branching off C15 by reaction of ketone **5** with any of the common methylenating agents failed;^6^ only after considerable simplification of the substitution pattern, including reduction of the C18 ester group and removal of the side chain at the furan C6 position, could this transformation be accomplished and product **7** be secured, although still in modest yield.^6^

In consideration of these critical issues, we conjectured that a building block such as **3** should be adequate and promising, as it features the properly configured C17–OH group, a handle for (late-stage) unveiling of the *exo*-methylene substituent, the vinyl group for use in RCM, as well as the correct oxidation state at C18. In contrast to the routes reported by the groups of Mulzer and White, which relied on chiral resolution or recourse to the chiral pool (d-glucose), respectively, we envisaged an alternative strategy in which the configuration of a single peripheral stereocenter to be set by asymmetric catalysis is relayed with high fidelity to the four chiral centers on the cyclobutane ring of **3**. To this end, an intramolecular photosensitized [2 + 2] cycloaddition reaction of an appropriate alkenylboronate was deemed the method of choice.^12,13^ As will be shown below, this approach affords both enantiomeric forms of the envisaged key compound **3** starting from a single photosubstrate; this stereochemical divergence is favorable because the absolute configuration of providencin remains unknown.^1^ Adaptation of the literature route that transformed **4** into **2**(4) should allow compound **3** to be elaborated into actual providencin (**1**); moreover, **3** provides opportunities to explore yet other conceivable gateways to the strained macrocyclic array of this still elusive target.

Inexpensive 3-furoic acid (**8**) was converted into the known bromide **9**,^14^ which was coupled to alkenyl boronate **11** (Scheme 2); the latter is available at scale upon esterification of commercial 4-pentynoic acid (**10**), hydroboration with catechol borane, followed by pinacol-for-catechol exchange. The ester terminus of **12** was then converted via the Weinreb amide^15^ into the corresponding TMS-capped alkynoate **13** as an adequate substrate for Noyori transfer hydrogenation.^16^ As expected,^17^ this transformation worked exceedingly well and provided multigram amounts of propargyl alcohol **15** with virtually perfect optical purity and quantitative yield. Base-mediated cleavage of the TMS-group preceded protection of the alcohol and hydroboration of the terminal alkyne upon treatment with pinB–H at elevated temperature to give product **16**; this reaction was promoted by catalytic amounts of the 9-*H*-9-BBN dimer.^18−20^

**Scheme 2:**
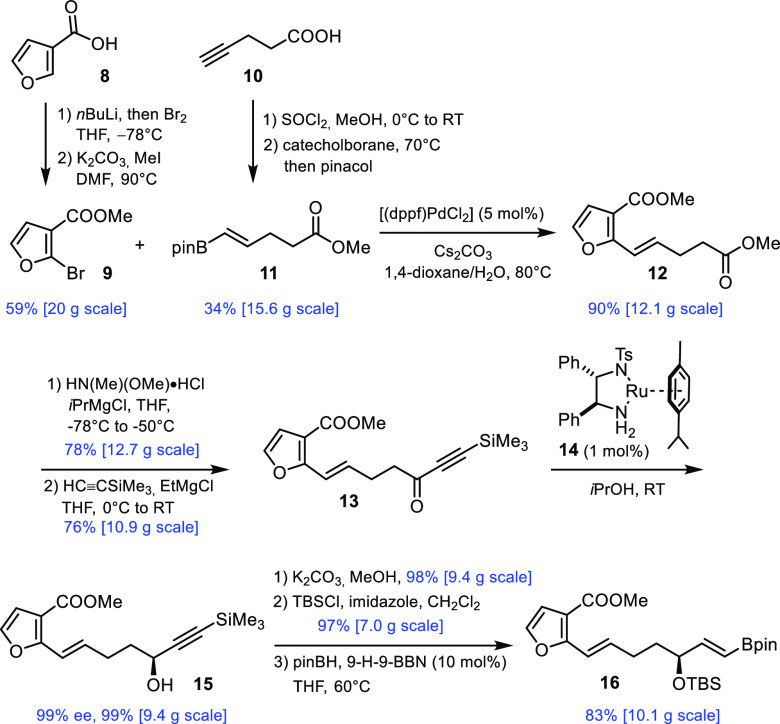
Preparation of the Substrate for the Photosensitized [2 + 2] Cycloaddition, All reactions in this and the other Schemes were performed at ambient temperature, unless stated otherwise. The scales shown in the Schemes refer to the amount of substrate in the single largest batch

With good quantities of **16** in hand, the stage was set for the formation of the cyclobutane ring via photosensitized intramolecular [2 + 2] cycloaddition (Scheme 3).^12^ Specifically, irradiation of a solution of **16** in carefully degassed MeCN with a blue LED in the presence of the iridium complex **17** (1 mol %) as photocatalyst resulted in the clean formation of the two diastereomeric products **18** and **19**, which were readily separated by flash chromatography. This transformation proved nicely scalable and practical: because the blue light is not absorbed by either glass or water, an ordinary jacketed vessel could be used to keep the temperature of the mixture constant throughout the course of the reaction (ca. 4 h, 10 g scale; for details see the Supporting Information). Although the dr of the photocycloaddition (≈1.5:1) is modest,^21^ it is pointed out that the resulting products **18** and **19** are “quasi-enantiomers” if one disregards the stereocenter carrying the −OTBS group; therefore, they can be used to access one or the other enantiomer of the targeted building block **3**, as outlined below, which is in keeping—in an unorthodox way—with the concept of atom economy.^22^

**Scheme 3:**
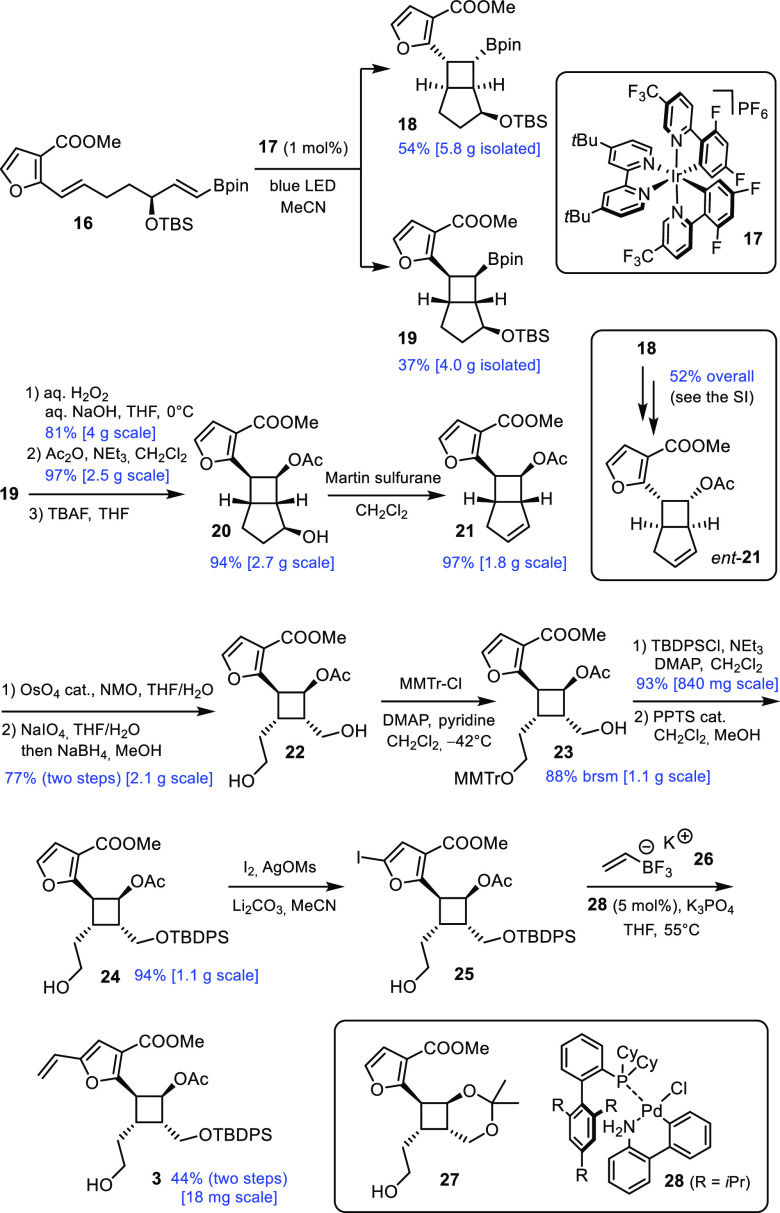
Formation and Elaboration of the Photocycloadducts

Oxidation of the C–B bond of **19** under standard conditions furnished the corresponding alcohol with retention of the configuration. Standard protecting group management then led to compound **20** in readiness for elimination upon exposure to Martin sulfurane,^23^ which proceeded again in almost quantitative yield to give alkene **21**. As already alluded to above, the same sequence of reactions applied to the diastereomeric cyclobutylboronate **18** formed by the photosensitized [2 + 2] cycloaddition reaction leads to the enantiomeric building block *ent*-**21** (for details, see the Supporting Information).

While attempted ozonolysis of **21** resulted in decomposition, a sequence of catalytic dihydroxylation followed by periodate cleavage and instant reduction of the crude dialdehyde furnished diol **22** in a good overall yield. Although its primary alcohols can be differentiated by acetonide formation, this tactic proved erratic because of the high sensitivity of the *trans*-annulated acetal ring in **27**.^24^ Therefore, we resorted to selective tritylation with (*p*-methoxyphenyl)diphenylmethyl chloride at low temperature;^4^ although not perfectly selective, the reaction proved high-yielding, robust, and scalable when combined with one round of recycling of the byproducts to the starting diol **22** (see the Supporting Information). Protection of the remaining −OH group in **23** as the corresponding TBDPS-ether, followed by trityl cleavage, afforded **24**. Gratifyingly, this compound proved amenable to regioselective iodination with the aid of a highly electrophilic sulfonyl hypoiodide generated in situ from I_2_ and AgOMs in MeCN (in connection with a modified work up procedure; see the Supporting Information);^25^ in this context, it is of note that more conventional halogenating agents, such as NBS, caused rapid decomposition. As expected, iodofuran **25** is exceptionally sensitive and needed to be directly used in the next step. Importantly, however, it could be engaged in a Suzuki coupling with vinyltrifluoroborate salt **26**(26,27) to furnish the targeted building block **3**; if elaborated by the route that allowed the Mulzer group to reach 17-deoxyprovidencin (**2**),^4^**3** is expected to open entry into actual providencin (**1**).

Rather than test this option right away, we were tempted to explore a potential—though risky—alternative. Incorporation of an alkyne into a macrocycle is obviously counterproductive whenever angle strain is a major issue; if ring strain is largely derived from transannular interactions, however, a slim and conical triple bond may actually be an advantage.^28^ This notion is manifested in earlier work by our group: thus, access to the very strained 12-membered lactone moiety of the putative cell migration inhibitor lactimidomycin^29,30^ via ring-closing alkyne metathesis (RCAM)^31,32^ had turned out to be much higher-yielding and robust than approaches to this and related targets on the basis of RCM.^33,34^ Equally instructive are the RCAM-based entries into the congested *nor*-cembranoid sinulariadiolide^35^ and the MAP-kinase inhibitor manshurolide.^36^ Actually, the latter consists of a triunsaturated carbocyclic ring bridging a butenolide in a way reminiscent of that of the providencin framework (**1**).

Although we were fully aware that any extrapolation of these cases to **1** might be misleading, we could not help but explore the use of RCAM as a way to forge its skeleton (Scheme 4). To this end, 3-butyn-1-ol (**29**) was elaborated into iodide **31**, as previously described during the synthesis of manshurolide,^36^ except that an asymmetric propynylation step was implemented.^37,38^ Subsequent MOM-protection followed by metal/halogen exchange and borylation of the transient organolithium species furnished alkenylboronate **33**. Suzuki reaction with **25** provided **34** in an unoptimized 39% yield over two steps; the low yield is largely due to the sensitivity of iodofuran **25**. The primary alcohol group was then oxidized, and propynylmagnesium chloride was added to the resulting aldehyde to give diyne **35** as a mixture of diastereomers.^39^ Unfortunately, this compound was not compliant and could not be cyclized even upon treatment with the most active and selective alkyne metathesis catalysts known to date;^36,40,41^ rather, a mixture of oligomers was formed (for more information, see the Supporting Information).

**Scheme 4:**
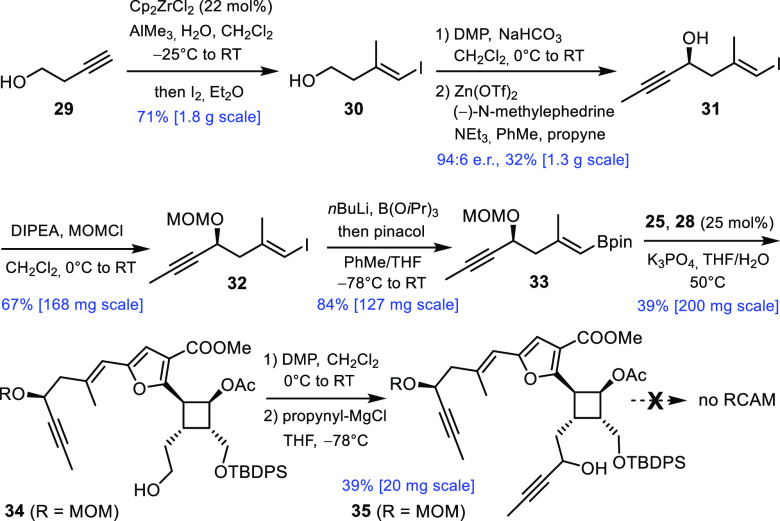
Attempted Macrocyclization by RCAM

Therefore, we must conclude that largely entropy-driven RCAM is not a viable option en route to providencin if a “furan/cyclobutane-first” strategy is pursued, as described herein. For good access to the fully functionalized building block **3**, however, the opportunity to resort to the literature route cited above remains open.^4^ Moreover, yet other methodologies might allow the exceptionally strained scaffold of this target to be forged, as long as the ring closure is accompanied by massive enthalpic gain; ventures along these lines are subject to ongoing studies in this laboratory.

## Data Availability

The data underlying this study are available in the published article and its Supporting Information.

## Supporting Information Available

Detailed experimental procedures, analytical and spectroscopic data, and copies of spectra of new compounds (PDF)
