# Supplementary material for: Studies toward Providencin: The Furanyl-Cyclobutanol Segment
Source: Org Lett. 2023 Feb 27;25(9):1536–40. doi: 10.1021/acs.orglett.3c00327 (PMC10012265; doi:10.1021/acs.orglett.3c00327)
Supplement: Supplementary file 1 — ol3c00327_si_001.pdf [file ol3c00327_si_001.pdf]

Supporting Information

Studies towards Providencin: The Furanyl-Cyclobutanol Segment

Simon M. Spohr and Alois Fürstner*

Max-Planck-Institut für Kohlenforschung, D-45478 Mülheim/Ruhr, Germany

Email: fuerstner@kofo.mpg.de

Table of Contents

General	S-2
Building Blocks	S-3
Photosensitized [2+2] Cycloaddition	S-8
Elaboration of the Photocycloadducts	S-9
Attempted RCAM	S-18
Copies of Spectra	S-23
References	S-61

General. Unless stated otherwise, all reactions were carried out in flame-dried glassware using anhydrous solvents under an argon atmosphere. The following solvents were purified by distillation over the indicated drying agents and were transferred under an argon atmosphere: THF, Et_2O (Mg/anthracene); MeCN, 2,6-lutidine, CH_2Cl_2, DCE (CaH_2); toluene (Na/K alloy); MeOH (Mg; stored over MS 3 Å). DMSO, DMF, NEt_3, pentane and pyridine were dried by an adsorption solvent purification system based on molecular sieves. Molecular sieves (5 Å) were activated at 150 °C for 24 h in high vacuum (1×10^{-3} mbar) and stored under argon.

Thin layer chromatography (TLC): Macherey-Nagel precoated plates (POLYGRAM®SIL/UV254); detection was achieved under UV-Light (254 nm) and by staining with either acidic *p*-anisaldehyde, cerium ammonium molybdenate or basic $KMnO_4$ solution. Flash chromatography: Merck silica gel 60 (40–63 µm) with predistilled or HPLC grade solvents. Preparative LC was performed with an Agilent 1260 infinity prep system (fraction collector G7159 B + G7166A, diode array detector G7115A); stationary phase and conditions for each compound are specified below.

NMR: Spectra were recorded on Bruker AV 400, AV 500, AVIII 600 or AVneo 600 spectrometers in the solvents indicated; chemical shifts (δ) are given in ppm relative to TMS, coupling constants (*J*) in Hz. The solvent signals were used as references and the chemical shifts converted to the TMS scale ($CDCl_3$: δ_C = 77.00 ppm; residual $CHCl_3$ in $CDCl_3$: δ_H = 7.26 ppm; CD_2Cl_2: δ_C = 53.84 ppm; residual $CDHCl_2$ in CD_2Cl_2: δ_H = 5.32 ppm; CD_3OD: δ_C = 49.00 ppm, residual CD_2HOD in CD_3OD: δ_H = 3.31 ppm; $(CD_3)_2SO$: δ_C = 39.52 ppm, residual CD_2HSOCD_3 in $(CD_3)_2SO$: δ_H = 2.50 ppm); all spectra were recorded at 25 °C. Multiplicities are indicated by the following abbreviations: s: singlet, d: doublet, t: triplet, q: quartet, p: pentet, h: hextet, hept: heptet, m: multiplet, br s: broad singlet, qd: quartet of doublet, ddq: doublet-doublet of quartet, dtdd: doublet of triplet of doublet of doublet, tddd: triplet of doublet-doublet-doublet. ^{13}C NMR spectra were recorded in ^1H-decoupled manner and the values of the chemical shifts are rounded to one decimal point. Signal assignments were established using HSQC, HMBC, COSY, NOESY and other 2D experiments.

IR: Spectra were recorded on an Alpha Platinum ATR instrument (Bruker); wavenumbers ($\tilde{\nu}$) in cm^{-1}.

MS (ESI-MS): Finnigan MAT 8200 (70 eV), ESI-MS: ESQ3000 (Bruker), accurate mass determinations: Bruker APEX III FTMS (7 T magnet) or Mat 95 (Finnigan).

Optical rotations ($[\alpha]_D^{20}$) were measured with an A-Krüss Otronic Model P8000-t polarimeter at a wavelength of 589 nm.

Unless stated otherwise, all compounds were commercially available (Alfa Aesar, Aldrich, TCI, Strem Chemicals, ChemPUR) and used as received.

Building Blocks

Methyl 2-bromofuran-3-carboxylate (9). An oven-dried 2 L jacketed vessel equipped with a dropping

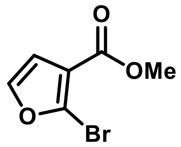

funnel was charged with 3-furoic acid (**8**) (19.73 g, 176 mmol) and THF (800 mL). The resulting solution was cooled to –78 °C before *n*-BuLi (1.6 M in hexanes, 231 mL, 370 mmol) was added dropwise over 2 h. Once the addition was complete, stirring was continued for another 2 h at –78 °C. Next, bromine (9.9 mL, 194 mmol) was added dropwise at this temperature and the resulting mixture was stirred for another 2 h. HCl (1 M, 100 mL) was added and the mixture warmed to rt. The resulting mixture was concentrated *in vacuo* until approximately 100 mL were left before it was diluted with additional HCl (1 M, 200 mL). The aqueous phase was extracted with EtOAc (3 x 350 mL) and the combined organic layers were dried over Na_2SO_4 and concentrated *in vacuo*. The resulting crude 2-bromo-3-carboxylic acid was used in the next step without further purification.

A 1 L round bottom flask equipped with a reflux condenser was charged with this crude material (27.5 g, 144 mmol) and DMF (440 mL). Potassium carbonate (60 g, 432 mmol) was added and the resulting mixture heated to 90 °C (oil bath) for 1.5 h. Next, iodomethane (17.9 mL, 288 mmol) was added and stirring continued for another 12 h at 90 °C. After reaching ambient temperature, water (200 mL) was added. The aqueous phase was extracted with Et_2O (3 x 400 mL) and the combined organic extracts were washed with water (200 mL), brine (100 mL) and dried over sodium sulfate. Concentration *in vacuo* furnished a residue, which was purified by flash chromatography on silica (pentane/*tert*-butyl methyl ether, 9:1) to give the title compound as a white solid (21.4 g, 59% yield over 2 steps). The spectral data are in accordance with the literature.[1] ^{1}H NMR (400 MHz, $CDCl_3$): δ = 7.43 (d, *J* = 2.2 Hz, 1H), 6.76 (d, *J* = 2.1 Hz, 1H), 3.86 (s, 3H); ^{13}C NMR (101 MHz, $CDCl_3$): δ = 162.3, 144.3, 129.0, 117.3, 112.7, 51.8. HRMS (EI): *m/z* calcd. for $C_6H_5O_3Br$ [M^+]: 203.9416, found: 203.9416.

Methyl pent-4-ynoate (S1). Thionyl chloride (21.3 mL, 293 mmol) was added over 30 min to a stirred

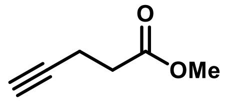

solution of pent-4-ynoic acid (**10**) (25 g, 255 mmol) in methanol (200 mL) at 0 °C. The resulting mixture was stirred for 2 h at room temperature before it was concentrated. The residue was dissolved in CH_2Cl_2 (300 mL) and the solution was successively washed with water (50 mL), sat. aq. $NaHCO_3$ (50 mL) and water (50 mL). The organic phase was dried over $MgSO_4$ and concentrated *in vacuo* (heating: 40 °C, pressure: > 250 mbar) to provide the title compound as a brown oil (22.7 g, 79% yield). The spectral data are in accordance with the literature.[2] ^{1}H NMR (400 MHz, $CDCl_3$): δ = 3.70 (s, 3H), 2.61 – 2.53 (m, 2H), 2.53 – 2.47 (m, 2H), 1.97 (t, *J* = 2.5 Hz, 1H). ^{13}C NMR (101 MHz, $CDCl_3$): δ = 172.2, 82.4, 69.0, 51.8, 33.1, 14.3.

Methyl (*E*)-5-(4,4,5,5-tetramethyl-1,3,2-dioxaborolan-2-yl)pent-4-enoate (11). Catecholborane

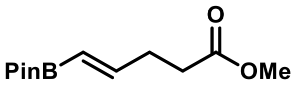

(17.8 mL, 167 mmol) was added over 1 h to a stirred solution of methyl pent-4-ynoate (15.6 g, 139 mmol) at room temperature. The resulting mixture was then stirred at 70 °C (oil bath) for 12 h before it was cooled to room temperature. Pinacol (23.0 g, 195 mmol) was added as a solid and the resulting mixture was vigorously stirred for 3 h at room temperature. The mixture was diluted with Et_2O (500 mL) and the organic phase washed with NaOH (1 M, 2 x 100 mL) and brine (100 mL). The organic layer was dried over Na_2SO_4 and concentrated *in vacuo*. The residue was purified by flash chromatography on silica (pentane:Et_2O, 40:1), furnishing the title compound as a colorless oil (11.4 g, 34% yield). ^{1}H NMR (400 MHz, $CDCl_3$): δ = 6.61 (dt, *J* = 18.0, 5.8 Hz, 1H), 5.46 (dt, *J* = 18.0, 1.6 Hz, 1H), 3.67 (s, 3H), 2.52 – 2.41 (m, 4H), 1.26 (s, 12H); ^{13}C NMR (101 MHz, $CDCl_3$): δ = 173.3, 151.6, 83.1, 51.6, 32.5, 30.5, 24.8; IR (film): $\tilde{\nu}$ = 2979, 1740, 1640, 1438, 1398, 1362, 1322, 1268, 1212, 1165, 1144, 1112, 1004, 971, 896, 850 cm^{-1}. HRMS (ESI): *m/z* calcd. for $C_{12}H_{21}O_4B$ [M$^+$]: 240.1527, found: 240.1529.

Methyl (*E*)-2-(5-methoxy-5-oxopent-1-en-1-yl)furan-3-carboxylate (12). 1,4-Dioxane (85 mL) and

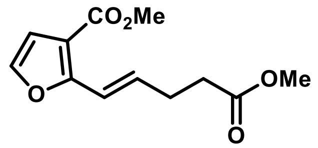

degassed water (8.5 mL) were added to a flask charged with methyl 2-bromofuran-3-carboxylate **9** (12.15 g, 59.25 mmol), boronate **11** (15.65 g, 65.18 mmol), Pd(dppf)Cl_2 (2.17 g, 2.96 mmol, 0.05 eq.) and Cs_2CO_3 (42.47 g, 130.36 mmol). The resulting mixture was stirred at 85 °C (oil bath) for 4 h before it was cooled to room temperature. The mixture was diluted with water (100 mL) and the aqueous phase extracted with Et_2O (3 x 250 mL). The combined extracts were dried over Na_2SO_4 and concentrated *in vacuo*. The residue was purified by flash chromatography on silica (pentane:Et_2O, 4.5:1) to give the title compound as a colorless oil (12.70 g, 90% yield). ^{1}H NMR (400 MHz, $CDCl_3$): δ = 7.24 (d, *J* = 2.0 Hz, 1H), 6.99 (dt, *J* = 16.1, 1.6 Hz, 1H), 6.67 (d, *J* = 2.0 Hz, 1H), 6.48 (dt, *J* = 16.0, 6.8 Hz, 1H), 3.83 (s, 3H), 3.70 (s, 3H), 2.63 – 2.55 (m, 2H), 2.51 (ddd, *J* = 8.6, 6.5, 1.7 Hz, 2H); ^{13}C NMR (101 MHz, $CDCl_3$): δ = 173.1, 164.0, 156.4, 141.0, 133.4, 118.7, 112.8, 111.5, 51.7, 51.5, 33.3, 28.2; IR (film): $\tilde{\nu}$ = 2953, 1735, 1713, 1655, 1569, 1508, 1437, 1410, 1364, 1303, 1260, 1195, 1156, 1093, 1050, 1032, 971, 941, 893, 846, 808, 780, 746, 600, 564 cm^{-1}. HRMS (ESI): *m/z* calcd. for $C_{12}H_{14}O_5$ [M$^+$]: 238.0835, found: 238.0836.

Methyl (*E*)-2-(5-(methoxy(methyl)amino)-5-oxopent-1-en-1-yl)furan-3-carboxylate (S2). *N,O*-

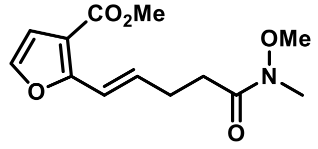

Dimethylhydroxylamine hydrochloride (6.24 g, 63.97 mmol) was added to stirred solution of ester **12** (12.70 g, 53.31 mmol) in THF (450 mL). The resulting mixture was cooled to –78 °C before *i*-PrMgCl (2 M in THF, 64 mL, 127.94 mmol) was added dropwise over the course of 1 h. The mixture was warmed to –50 °C and stirred at this temperature for 30 min before the reaction was quenched with sat. aq. NH_4Cl (150 mL). After reaching room temperature, the aqueous layer was extracted with Et_2O (3 x 250 mL) and the

combined extracts were dried over Na_2SO_4 and concentrated *in vacuo*. The residue was purified by flash chromatography on silica (pentane:Et_2O, 1:2) to give the title compound as a pale yellow oil (11.09 g, 78% yield). ^1H NMR (400 MHz, $CDCl_3$): δ = 7.23 (d, *J* = 1.9 Hz, 1H), 6.99 (dt, *J* = 16.0, 1.4 Hz, 1H), 6.67 (d, *J* = 2.0 Hz, 1H), 6.60 – 6.50 (m, 1H), 3.83 (s, 3H), 3.69 (s, 3H), 3.19 (s, 3H), 2.66 – 2.56 (m, 4H); ^{13}C NMR (101 MHz, $CDCl_3$): δ = 164.0, 156.7, 140.9, 134.5, 118.4, 112.6, 111.4, 61.3, 51.4, 32.2, 31.2, 30.3, 27.9; IR (film): $\tilde{\nu} = 2952$, 1715, 1660, 1569, 1509, 1440, 1413, 1386, 1305, 1264, 1198, 1162, 1138, 1049, 1033, 994, 973, 747 cm^{-1}. HRMS (ESI): *m/z* calcd. for $C_{13}H_{18}O_5N$ [$M+H^+$]: 268.1179, found: 268.1181.

Methyl (*E*)-2-(5-oxo-7-(trimethylsilyl)hept-1-en-6-yn-1-yl)furan-3-carboxylate (13). EtMgCl (2 M in THF, 22.4 mL, 44.8 mmol) was added dropwise to a stirred solution of trimethylsilylacetylene (6.3 mL, 44.8 mmol) in THF (200 mL) at 0 °C. The resulting mixture was stirred at 0 °C for 1 h and at room temperature for 30 min. The mixture was then cooled to 0 °C and added to a solution of Weinreb amide **S2** (10.89 g, 40.74 mmol) in THF (50 mL) at 0 °C via cannula. After stirring at 0 °C for 5 min and at room temperature for 1 h, the reaction was quenched with sat. aq. NH_4Cl (100 mL). The aqueous phase was extracted with Et_2O (3 x 250 mL), the combined extracts were washed with brine (100 mL), dried over Na_2SO_4 and concentrated *in vacuo*. The residue was purified by flash chromatography on silica (pentane:Et_2O, 9:1), furnishing the title compound as a colorless oil (9.48 g, 76% yield). ^1H NMR (400 MHz, $CDCl_3$): δ = 7.24 (d, *J* = 2.0 Hz, 1H), 6.98 (dt, *J* = 16.0, 1.6 Hz, 1H), 6.67 (d, *J* = 2.0 Hz, 1H), 6.47 (dt, *J* = 16.0, 6.9 Hz, 1H), 3.83 (s, 3H), 2.77 (td, *J* = 7.2, 0.8 Hz, 2H), 2.65 – 2.57 (m, 2H), 0.24 (s, 9H); ^{13}C NMR (101 MHz, $CDCl_3$): δ = 186.3, 164.0, 156.4, 141.0, 133.1, 118.8, 112.8, 111.4, 101.8, 98.5, 51.5, 44.2, 27.1, –0.8; IR (film): $\tilde{\nu} = 2956$, 1715, 1675, 1569, 1508, 1439, 1409, 1303, 1252, 1197, 1163, 1137, 1113, 1081, 1047, 1033, 972, 940, 844, 760, 705, 599 cm^{-1}. HRMS (ESI): *m/z* calcd. for $C_{16}H_{20}O_4SiNa$ [$M+Na^+$]: 327.1023, found: 327.1024.

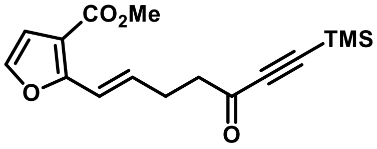

Methyl (*S,E*)-2-(5-hydroxy-7-(trimethylsilyl)hept-1-en-6-yn-1-yl)furan-3-carboxylate (15). Ru(*p*-cymene)[(*S,S*)-Ts-DPEN] (**14**) (187 mg, 0.31 mmol, 0.01 eq.) was added to *i*-PrOH (200 mL) and the mixture vigorously stirred until a faint orange solution had formed. A solution of ynone **13** (9.48 g, 31.14 mmol) in *i*-PrOH (20 mL) was added dropwise, causing a color change to bright pink. The mixture was stirred for 1 h at room temperature before it was concentrated *in vacuo*. The residue was purified by flash chromatography on silica (pentane:*tert*-butyl methyl ether, 4:1) to afford the title compound as a colorless oil (9.39 g, 99% yield, 99% ee). $[\alpha]_D^{20}$ = +34.8° (*c* = 1.0, $CHCl_3$); ^1H NMR (400 MHz, $CDCl_3$): = 7.28 – 7.20 (m, 1H), 7.03 – 6.94 (m, 1H), 6.69 – 6.64 (m, 1H), 6.58 – 6.45 (m, 1H), 4.46 – 4.38 (m, 1H), 3.85 – 3.81 (m, 2H), 2.49 – 2.39 (m, 2H), 1.93 – 1.84 (m, 2H), 1.79 – 1.71 (m, 1H), 0.20 – 0.15 (m, 9H);

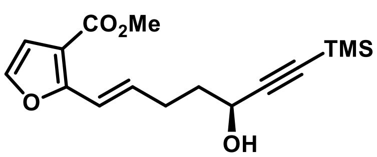

^{13}C NMR (101 MHz, $CDCl_3$): δ = 164.1, 156.7, 140.9, 134.7, 118.4, 112.5, 111.4, 106.2, 90.0, 62.2, 51.5, 36.7, 28.7, –0.2; IR (film): $\tilde{\nu}$ = 3427, 2954, 1717, 1653, 1568, 1509, 1441, 1410, 1304, 1250, 1198, 1161, 1139, 1105, 1053, 1035, 971, 942, 892, 843, 760 cm^{-1}. HRMS (ESI): *m/z* calcd. for $C_{16}H_{22}O_4SiNa$ [M+Na^+]: 329.1179, found: 329.1183.

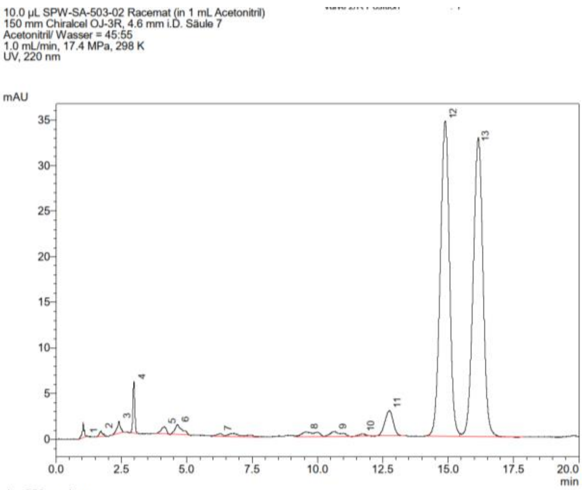

1 220nm,4nm

PDA Ch1 220nm

Peak #	Ret. Time	Area %	Name
1	1.13	0.43	
2	1.71	0.26	
3	2.40	0.63	
4	2.98	1.49	
5	4.13	0.56	
6	4.64	1.10	
7	6.27	0.98	
8	9.57	1.18	
9	10.67	0.90	
10	11.73	0.23	
11	12.74	3.24	
12	14.88	44.31	1st enantiomer
13	16.12	44.69	2nd enantiomer
Total		100.00	

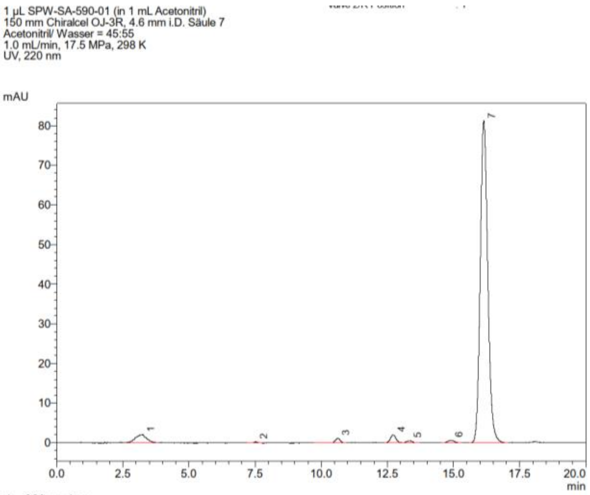

1 220nm,4nm

PDA Ch1 220nm

Peak #	Ret. Time	Area %	Name	
1	3.24	3.38		
2	7.51	0.16		
3	10.62	0.92		
4	12.73	1.61		
5	13.34	0.50		
6	14.90	0.63	1st enantiomer	ee = 98.7 %
7	16.14	92.80	2nd enantiomer	
Total		100.00		

Figure S1. HPLC-traces of *rac*-**15** (left) and enantioenriched **15** (right): t_R = 14.90 min (minor enantiomer) and 16.14 min (major enantiomer) (Chiralcel OJ-3R column, λ = 220 nm, isocratic elution 45:55 acetonitrile/water, flow-rate = 1.0 mL/min)

Methyl (*S,E*)-2-(5-hydroxyhept-1-en-6-yn-1-yl)furan-3-carboxylate (S3). Potassium carbonate (12.7 g, 91.88 mmol) was added to a stirred solution of alcohol **15** (9.39 g, 30.66 mmol) in methanol (136 mL) and the resulting yellow mixture was stirred for 30 min at room temperature. The reaction was quenched upon addition of sat. aq. NH_4Cl (50 mL) and water (80 mL). The aqueous phase was extracted with CH_2Cl_2 (3 x 200 mL) and the combined extracts were dried over Na_2SO_4 and concentrated. The residue was purified by flash chromatography on silica (hexanes:*tert*-butyl methyl ether, 2:1), providing the title compound as a colorless oil (7.01 g, 98% yield). $[\alpha]_D^{20}$ = +21.9° (*c* = 1.0, $CHCl_3$); ^1H NMR (400 MHz, $CDCl_3$): δ = 7.24 (d, *J* = 2.0 Hz, 1H), 7.03 – 6.96 (m, 1H), 6.67 (d, *J* = 2.0 Hz, 1H), 6.50 (dt, *J* = 16.0, 7.1 Hz, 1H), 4.44 (td, *J* = 6.5, 2.1 Hz, 1H), 3.83 (s, 3H), 2.50 (d, *J* = 2.1 Hz, 1H), 2.46 (qd, *J* = 7.1, 1.3 Hz, 2H), 1.98 (br s, 1H), 1.95 – 1.87 (m, 2H); ^{13}C NMR (101 MHz, $CDCl_3$): δ = 164.1, 156.7, 140.9, 134.5, 118.6, 112.5, 111.4, 84.4, 73.4, 61.6, 51.5, 36.6, 28.5; IR (film): $\tilde{\nu}$ = 3436, 3292, 2952,

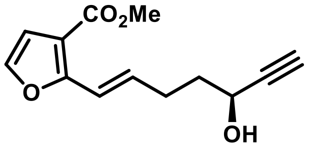

1710, 1652, 1568, 1509, 1440, 1409, 1304, 1263, 1199, 1160, 1137, 1103, 1051, 1033, 971, 939, 892, 747, 661, 600, 569 cm^{-1}. HRMS (ESI): *m/z* calcd. for $C_{13}H_{14}O_4Na$ [M+Na$^+$]: 257.0784, found: 257.0786.

Methyl (*S,E*)-2-(5-((*tert*-butyldimethylsilyl)oxy)hept-1-en-6-yn-1-yl)furan-3-carboxylate (S4).

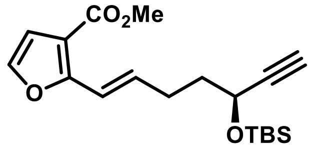

Imidazole (2.24 g, 32.87 mmol) and *tert*-butyldimethylsilyl chloride (4.95 g, 32.87 mmol) were added to a stirred solution of alcohol **S3** (7.00 g, 29.88 mmol) in CH_2Cl_2 (80 mL), and the resulting mixture was stirred for 16 h at room temperature. The reaction was quenched with sat. aq. $NaHCO_3$ (40 mL) and the aqueous phase was extracted with CH_2Cl_2 (3 x 150 mL). The combined extracts were dried over Na_2SO_4 and concentrated, and the residue was purified by flash chromatography on silica (pentane:*tert*-butyl methyl ether, 9:1) to afford the title compound as a colorless oil (10.13 g, 97% yield). $[\alpha]_{\mathrm{D}}^{20}$ = –13.1° (*c* = 2.0, $CHCl_3$); ^{1}H NMR (400 MHz, $CDCl_3$): δ = 7.24 (d, *J* = 2.0 Hz, 1H), 6.97 (dt, *J* = 16.0, 1.5 Hz, 1H), 6.67 (d, *J* = 2.0 Hz, 1H), 6.51 (dt, *J* = 16.0, 7.1 Hz, 1H), 4.41 (td, *J* = 6.2, 2.1 Hz, 1H), 3.84 (s, 3H), 2.46 – 2.38 (m, 3H), 1.91 – 1.83 (m, 2H), 0.91 (s, 9H), 0.13 (d, *J* = 11.2 Hz, 6H); ^{13}C NMR (101 MHz, $CDCl_3$): δ = 164.1, 156.8, 140.8, 135.1, 118.2, 112.4, 111.4, 85.2, 72.5, 62.1, 51.4, 37.7, 28.6, 25.8, 18.2, –4.6, –5.1; IR (film): $\tilde{\nu} = 2952$, 2930, 2886, 2857, 1717, 1654, 1569, 1509, 1471, 1462, 1439, 1410, 1361, 1302, 1259, 1197, 1161, 1139, 1088, 1054, 1035, 1005, 970, 941, 893, 837, 811, 778, 740, 661, 631, 599 cm^{-1}. HRMS (ESI): *m/z* calcd. for $C_{19}H_{29}O_4Si$ [M+H$^+$]: 349.1829, found: 349.1830.

Methyl 2-((*S*,1*E*,6*E*)-5-((*tert*-butyldimethylsilyl)oxy)-7-(4,4,5,5-tetramethyl-1,3,2-dioxaborolan-2-yl)hepta-1,6-dien-1-yl)furan-3-carboxylate (16). A solution of pinacol borane (6.3 mL, 43.47 mmol)

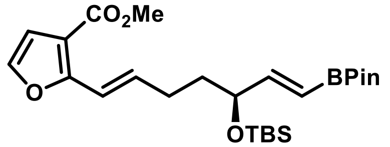

and 9-borabicyclo[3.3.1]nonane (9-H-9-BBN, 353 mg, 0.1 eq.) in THF (3 mL) was added to a stirred solution of alkyne **S4** (10.10 g, 28.98 mmol) in THF (60 mL). The resulting mixture was stirred at 60 °C (oil bath) for 16 h before the reaction was cautiously quenched at room temperature upon dropwise addition of sat. aq. NH_4Cl (20 mL). The aqueous phase was diluted with water (100 mL) and extracted with EtOAc (3 x 200 mL). The combined extracts were washed with brine (100 mL), dried over Na_2SO_4 and concentrated, and the residue was purified by flash chromatography on silica (hexanes:*tert*-butyl methyl ether, 15:1) to provide the title compound as a colorless oil (11.40 g, 83% yield). $[\alpha]_{\mathrm{D}}^{20}$ = +18.3° (*c* = 1.0, $CHCl_3$); ^{1}H NMR (400 MHz, $CDCl_3$): δ = 7.22 (d, *J* = 1.9 Hz, 1H), 6.93 (dt, *J* = 16.0, 1.6 Hz, 1H), 6.66 (d, *J* = 1.9 Hz, 1H), 6.60 – 6.45 (m, 2H), 5.62 (dd, *J* = 18.0, 1.5 Hz, 1H), 4.24 (qd, *J* = 5.8, 1.6 Hz, 1H), 3.83 (s, 3H), 2.30 (dddd, *J* = 8.7, 7.4, 6.3, 1.6 Hz, 2H), 1.68 (ddd, *J* = 9.7, 7.9, 5.9 Hz, 2H), 1.27 (s, 12H), 0.90 (s, 9H), 0.03 (d, *J* = 9.3 Hz, 6H); ^{13}C NMR (101 MHz, $CDCl_3$): δ = 164.1, 157.0, 155.5, 140.7, 136.1, 117.8, 112.2, 111.4, 83.1, 73.4, 51.4, 36.5, 28.4, 25.9, 24.8, 24.7, 18.2, –4.4, –4.9; IR (film): $\tilde{\nu} = 2977$, 2952, 2930, 2857, 1718, 1642, 1569, 1508, 1471, 1463, 1439, 1390, 1364, 1339, 1320, 1259, 1196,

1145, 1086, 1053, 1035, 999, 971, 941, 918, 895, 836, 810, 776, 739, 671, 666 cm^{-1}. HRMS (ESI): *m/z* calcd. for $C_{25}H_{41}O_6BSiNa$ [$M+Na^+$]: 499.2657, found: 499.2661.

Photosensitized [2+2] Cycloaddition

Cyclobutanes 18 and 19. The iridium complex **17** (252 mg, 0.22 mmol, 0.01 eq.) was added to a stirred

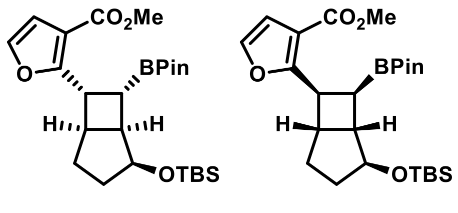

18 19

solution of alkenyl boronic ester **16** (10.70 g, 22.46 mmol) in dry and degassed (three freeze-pump-thaw cycles) MeCN (500 mL) in a 1L-jacketed vessel, which was connected to a stream of cooling water (T ≈ 14 °C). The mixture was irradiated with a blue LED bulb (Hepatochem, 475 nm) for 4 h (see Figure S2 for the reaction setup). The mixture was then concentrated *in vacuo* and the resulting residue purified by flash chromatography on fine silica (hexanes:EtOAc, 17:1) to furnish the diastereomeric products **18** (5.82 g, 54% yield) and **19** (4.03 g, 37% yield) as a colorless oil each.

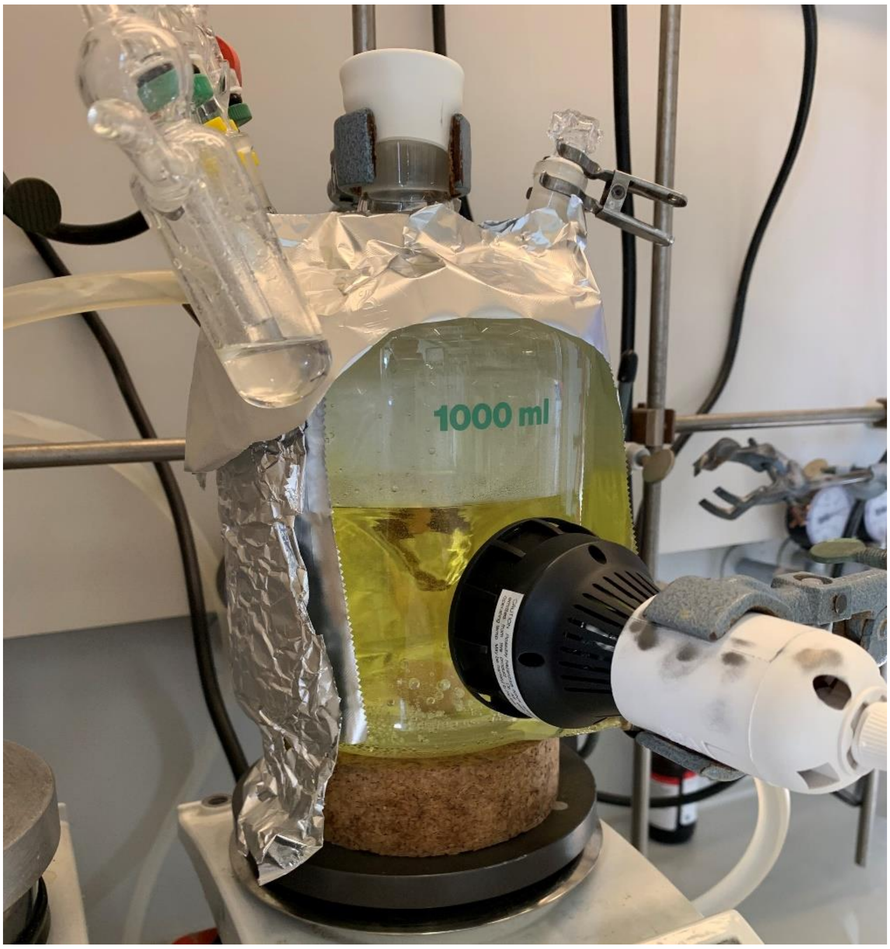

Figure S2. Reaction setup for the photosensitized [2+2] cycloaddition

Analytical and spectroscopic data of **18**: $[\alpha]_D^{20}$ = –69.1° (*c* = 1.0, $CHCl_3$); ^1H NMR (600 MHz, $CDCl_3$): δ = 7.26 (d, *J* = 2.0 Hz, 1H), 6.63 (d, *J* = 1.9 Hz, 1H), 4.22 (ddd, *J* = 10.0, 7.3, 6.3 Hz, 1H), 3.93 (ddd, *J* = 11.7, 4.8, 0.9 Hz, 1H), 3.79 (s, 3H), 2.93 (tdd, *J* = 6.6, 3.5, 1.1 Hz, 1H), 2.85 (q, *J* = 7.0 Hz, 1H), 2.46 (ddd, *J* = 11.6, 6.2, 1.2 Hz, 1H), 2.03 (tdd, *J* = 12.3, 10.0, 8.1 Hz, 1H), 1.93 – 1.85 (m, 1H), 1.64 – 1.57 (m, 2H), 1.11 (s, 6H), 1.04 (s, 6H), 0.87 (s, 9H), 0.05 (s, 3H), 0.02 (s, 3H); ^{13}C NMR (151 MHz, $CDCl_3$) δ 165.0, 164.4, 139.8, 112.3, 111.0, 82.8, 75.4, 51.2, 40.7, 39.2, 36.7, 32.2, 28.9, 25.9, 25.0, 24.6, 18.2, –4.85, –4.89; IR (film): $\tilde{\nu}$ = 2951, 2930, 2885, 2856, 1719, 1595, 1519, 1462, 1441, 1410, 1379, 1323, 1305, 1283, 1250, 1194, 1164, 1142, 1106, 1051, 1032, 1007, 987, 959, 940, 907, 875, 853, 836, 804, 775, 734, 669, 602 cm^{-1}. HRMS (ESI): *m/z* calcd. for $C_{25}H_{41}O_6BSiNa$ [M+Na$^+$]: 499.2657, found: 499.2659.

Analytical and spectroscopic data of **19**: $[\alpha]_D^{20}$ = +60.3° (*c* = 1.0, $CHCl_3$); ^1H NMR (600 MHz, $CDCl_3$): δ = 7.28 (d, *J* = 2.0 Hz, 1H), 6.62 (d, *J* = 2.0 Hz, 1H), 4.00 (d, *J* = 3.9 Hz, 1H), 3.82 (ddd, *J* = 11.4, 4.8, 1.0 Hz, 1H), 3.78 (s, 3H), 3.20 – 3.15 (m, 1H), 2.75 (t, *J* = 7.0 Hz, 1H), 2.17 (tdd, *J* = 13.1, 7.0, 4.0 Hz, 1H), 1.96 (tt, *J* = 12.9, 7.0 Hz, 1H), 1.83 – 1.77 (m, 1H), 1.72 (ddd, *J* = 11.4, 6.7, 1.2 Hz, 1H), 1.59 (dd, *J* = 12.8, 6.9 Hz, 1H), 1.11 (s, 6H), 1.05 (s, 6H), 0.86 (s, 9H), 0.04 (d, *J* = 1.5 Hz, 6H); ^{13}C NMR (151 MHz, $CDCl_3$): δ = 164.9, 164.3, 139.9, 112.2, 111.0, 82.9, 79.2, 51.2, 45.1, 41.3, 35.4, 33.7, 30.4, 25.9, 25.0, 24.4, 18.2, –4.8, –4.8; IR (film): $\tilde{\nu}$ = 2953, 2929, 2886, 2856, 1720, 1595, 1519, 1462, 1440, 1410, 1377, 1319, 1252, 1195, 1165, 1143, 1109, 1057, 1020, 973, 940, 891, 880, 854, 835, 808, 776, 734, 666 cm^{-1}. HRMS (ESI): *m/z* calcd. for $C_{25}H_{42}O_6BSi$ [M+H$^+$]: 477.2838, found: 477.2839.

Elaboration of the Photocycloadducts

Methyl 2-((1*R*,2*S*,5*S*,6*R*,7*S*)-2-((*tert*-butyldimethylsilyl)oxy)-7-hydroxybicyclo[3.2.0]heptan-6-yl)-furan-3-carboxylate (S5). A mixture (40 mL, 2:1 *v/v*) of aq. NaOH (2 M) and H_2O_2 (35% *w/w*) was added

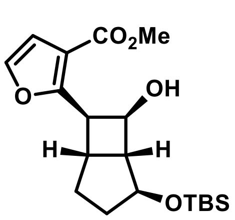

to a stirred solution of boronic ester **19** (4.01 g, 8.42 mmol) in THF (40 mL) at 0 °C and the resulting mixture was vigorously stirred at this temperature for 30 min. The reaction was carefully quenched with sat. aq. NH_4Cl (20 mL) and the mixture diluted with EtOAc (100 mL). The aqueous phase was extracted with EtOAc (3 x 150 mL), the combined extracts were washed with brine (50 mL), dried over Na_2SO_4 and concentrated *in vacuo*, and the residue was purified by flash chromatography on silica (hexanes:*tert*-butyl methyl ether, 3:1) to give the title compound as an amorphous white solid (2.58 g, 81% yield). $[\alpha]_D^{20}$ = +85.7° (*c* = 1.0, $CHCl_3$); ^1H NMR (400 MHz, $CDCl_3$): δ = 7.37 (d, *J* = 2.0 Hz, 1H), 6.69 (d, *J* = 2.0 Hz, 1H), 4.23 (t, *J* = 2.2 Hz, 1H), 4.04 (ddd, *J* = 7.9, 4.1, 0.8 Hz, 1H), 3.84 – 3.77 (m, 4H), 3.24 (td, *J* = 7.8, 4.8 Hz, 1H), 2.66 (ddt, *J* = 8.0, 4.0, 1.1 Hz, 1H), 2.06 – 1.94 (m, 1H), 1.87 – 1.79 (m, 2H), 1.61 – 1.52 (m, 1H), 0.87 (s, 9H), 0.06 (d, *J* = 1.8 Hz, 6H); ^{13}C NMR (101 MHz, $CDCl_3$): δ = 165.0, 160.8, 141.2, 114.2, 111.0, 76.6, 69.8, 56.7, 51.5, 43.2, 36.2, 34.8, 29.1, 25.8, 18.1, –4.67, –4.72; IR (film): $\tilde{\nu}$ = 2953, 2929, 2887, 2856, 1716, 1593, 1518, 1471, 1462, 1441, 1407, 1360, 1340, 1312, 1253, 1199, 1144, 1082, 1059,

1032, 1006, 940, 887, 835, 812, 772, 736 cm^{-1}. HRMS (ESI): *m/z* calcd. for $C_{19}H_{30}O_5SiNa$ [M+Na$^+$]: 389.1754, found: 389.1759.

Methyl 2-((1*R*,2*S*,5*S*,6*R*,7*S*)-7-acetoxy-2-((*tert*-butyldimethylsilyl)oxy)bicyclo[3.2.0]heptan-6-yl)-furan-3-carboxylate (S6). 4-Dimethylaminopyridine (72 mg, 0.59 mmol), triethylamine (2.89 mL, 20.74 mmol) and acetic anhydride (1.96 mL, 20.74 mmol) were added to a stirred solution of alcohol **S5** (2.53 g, 6.92 mmol) in CH_2Cl_2 (63 mL) at 0 °C. The mixture was stirred at room temperature for 1 h before the reaction was quenched with sat. aq. $NaHCO_3$ (30 mL). The aqueous phase was extracted with CH_2Cl_2 (3 x 100 mL), the combined extracts were dried over Na_2SO_4 and concentrated, and the residue was purified by flash chromatography on silica (hexanes:*tert*-butyl methyl ether, 9:1) to provide the title compound as a colorless oil (2.74 g, 97% yield). $[\alpha]_D^{20}$ = +59.8° (*c* = 1.0, $CHCl_3$); ^1H NMR (400 MHz, $CDCl_3$): δ = 7.37 (d, *J* = 2.0 Hz, 1H), 6.67 (d, *J* = 2.0 Hz, 1H), 4.76 (ddd, *J* = 8.2, 4.3, 0.8 Hz, 1H), 4.32 – 4.26 (m, 1H), 4.10 (ddd, *J* = 8.1, 4.7, 1.4 Hz, 1H), 3.79 (s, 3H), 3.30 (td, *J* = 7.7, 4.6 Hz, 1H), 2.82 – 2.73 (m, 1H), 2.05 – 1.82 (m, 3H), 1.78 (s, 3H), 1.62 (dd, *J* = 12.2, 6.1 Hz, 1H), 0.87 (s, 9H), 0.06 (s, 6H); ^{13}C NMR (101 MHz, $CDCl_3$): δ = 169.7, 164.1, 159.2, 141.1, 114.5, 110.8, 76.3, 70.4, 53.4, 51.4, 40.5, 37.0, 34.7, 29.1, 25.8, 20.5, 18.0, –4.77, –4.79; IR (film): $\tilde{\nu}$ = 2954, 2931, 2893, 2857, 1742, 1722, 1598, 1518, 1472, 1462, 1440, 1407, 1363, 1340, 1313, 1291, 1233, 1196, 1158, 1143, 1073, 1056, 1026, 940, 886, 837, 809, 776, 738 cm^{-1}. HRMS (ESI): *m/z* calcd. for $C_{21}H_{32}O_6SiNa$ [M+Na$^+$]: 431.1860, found: 431.1865.

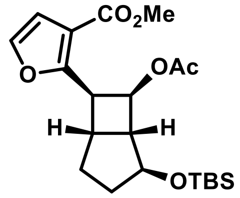

Methyl 2-((1*S*,2*S*,5*S*,6*R*,7*S*)-7-acetoxy-2-hydroxybicyclo[3.2.0]heptan-6-yl)furan-3-carboxylate (20). Tetrabutylammonium fluoride (1 M in THF, 8.05 mL, 8.05 mmol) was added to a stirred solution of silyl ether **S6** (2.74 g, 6.71 mmol) in THF (70 mL) at 0 °C. The mixture was stirred at room temperature for 5 h before the reaction was quenched with sat. aq. NH_4Cl (30 mL). The aqueous phase was extracted with CH_2Cl_2 (3 x 100 mL), the combined extracts were dried over Na_2SO_4 and concentrated, and the residue was purified by flash chromatography on silica (hexanes:EtOAc, 1:1) to give the title compound as a yellow oil (1.85 g, 94% yield). $[\alpha]_D^{20}$ = +58.5° (*c* = 1.0, $CHCl_3$); ^1H NMR (400 MHz, $CDCl_3$): δ = 7.38 (d, *J* = 2.0 Hz, 1H), 6.68 (d, *J* = 1.9 Hz, 1H), 4.82 (ddd, *J* = 8.1, 4.2, 0.8 Hz, 1H), 4.40 (d, *J* = 3.4 Hz, 1H), 4.14 (ddd, *J* = 8.1, 4.8, 1.4 Hz, 1H), 3.79 (s, 3H), 3.37 (ddd, *J* = 8.1, 6.0, 4.6 Hz, 1H), 2.89 – 2.83 (m, 1H), 2.09 – 1.92 (m, 3H), 1.79 (s, 3H), 1.73 – 1.68 (m, 1H); ^{13}C NMR (101 MHz, $CDCl_3$): δ = 169.7, 164.0, 158.8, 141.2, 114.6, 110.8, 76.1, 70.3, 53.1, 51.4, 40.5, 37.0, 34.3, 28.9, 20.5; IR (film): $\tilde{\nu}$ = 3449, 2952, 1739, 1720, 1596, 1518, 1441, 1407, 1375, 1339, 1310, 1292, 1234, 1198, 1158, 1063, 1035, 984, 935, 862, 805, 744, 605 cm^{-1}. HRMS (ESI): *m/z* calcd. for $C_{15}H_{18}O_6Na$ [M+Na$^+$]: 317.0995, found: 317.0997.

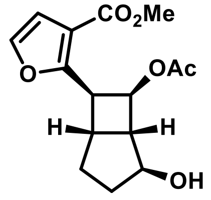

Methyl 2-((1*R*,5*S*,6*R*,7*S*)-7-acetoxybicyclo[3.2.0]hept-2-en-6-yl)furan-3-carboxylate (21). A solution

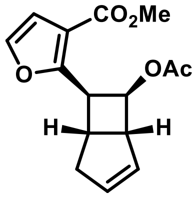

of Martin's sulfurane (6.26 g, 9.31 mmol) in CH_2Cl_2 (10 mL) was added to a stirred solution of alcohol **20** (1.83 g, 6.21 mmol) in CH_2Cl_2 (80 mL) and the resulting mixture was stirred for 5 h at ambient temperature before the reaction was quenched upon addition of sat. aq. $NaHCO_3$ (40 mL). The aqueous phase was extracted with CH_2Cl_2 (3 x 100 mL), the combined organic layers were dried over Na_2SO_4 and concentrated, and the residue was purified by flash chromatography on silica (hexanes:*tert*-butyl methyl ether, 8:1) to furnish the title compound as a yellow oil (1.66 g, 97% yield). $[\alpha]_D^{20}$ = +46.7° (*c* = 1.0, $CHCl_3$); ^1H NMR (400 MHz, $CDCl_3$): δ = 7.36 (d, *J* = 2.0 Hz, 1H), 6.68 (d, *J* = 2.0 Hz, 1H), 5.88 (s, 2H), 5.11 (dd, *J* = 6.8, 1.6 Hz, 1H), 4.22 (td, *J* = 7.1, 1.5 Hz, 1H), 3.79 (s, 3H), 3.63 – 3.55 (m, 1H), 3.34 – 3.27 (m, 1H), 2.69 – 2.58 (m, 1H), 2.36 (ddd, *J* = 17.2, 3.6, 1.3 Hz, 1H), 1.86 (s, 3H); ^{13}C NMR (101 MHz, $CDCl_3$): δ = 170.0, 164.0, 158.7, 141.1, 133.0, 129.7, 114.1, 111.0, 76.6, 51.5, 51.4, 42.5, 39.4, 36.9, 20.7; IR (film): $\tilde{\nu}$ = 2952, 2845, 1738, 1717, 1596, 1516, 1440, 1409, 1372, 1353, 1330, 1293, 1233, 1197, 1165, 1141, 1106, 1067, 1033, 950, 915, 876, 799, 753, 723, 603 cm^{-1}. HRMS (ESI): *m/z* calcd. for $C_{15}H_{16}O_5Na$ [M+Na$^+$]: 299.0889, found: 299.0891.

Methyl 2-((1*S*,2*S*,5*R*,6*S*,7*R*)-2-((*tert*-butyldimethylsilyl)oxy)-7-hydroxybicyclo[3.2.0]heptan-6-yl)furan-3-carboxylate (S7). A mixture (2:1 *v/v*, 4 mL) of aq. NaOH (2 M) and aq. H_2O_2 (35% *w/w*) was

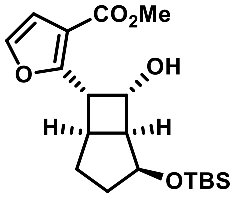

added to a stirred solution of boronic ester **18** (400 mg, 0.84 mmol) in THF (4 mL) at 0 °C. The resulting mixture was vigorously stirred at this temperature for 30 min before the reaction was carefully quenched with sat. aq. NH_4Cl (5 mL). The mixture was diluted with EtOAc (20 mL), the aqueous phase was extracted with EtOAc (3 x 100 mL), and the combined extracts were washed with brine (10 mL), dried over Na_2SO_4 and concentrated *in vacuo*. The residue was purified by flash chromatography on silica (hexanes:*tert*-butyl methyl ether, 7:1) to give the title compound as an amorphous white solid (264 mg, 86% yield). $[\alpha]_D^{20}$ = –104.4° (*c* = 1.0, $CHCl_3$); ^1H NMR (400 MHz, $CDCl_3$): δ = 7.35 (d, *J* = 2.0 Hz, 1H), 6.69 (d, *J* = 1.9 Hz, 1H), 4.66 (dd, *J* = 7.8, 3.3 Hz, 1H), 4.28 – 4.20 (m, 1H), 3.87 (ddd, *J* = 7.9, 5.3, 1.4 Hz, 1H), 3.82 (s, 3H), 3.15 – 3.07 (m, 1H), 2.62 (tdd, *J* = 7.9, 3.4, 1.4 Hz, 1H), 2.03 – 1.87 (m, 2H), 1.80 (tdd, *J* = 12.3, 9.7, 7.1 Hz, 1H), 1.72 – 1.53 (m, 2H), 0.90 (s, 9H), 0.07 (d, *J* = 2.9 Hz, 6H); ^{13}C NMR (101 MHz, $CDCl_3$): δ = 165.0, 160.9, 141.0, 114.0, 111.0, 74.1, 66.6, 51.5, 50.7, 44.4, 35.9, 33.4, 28.0, 25.9, 18.2, –4.77, –4.84; IR (film): $\tilde{\nu}$ = 3488, 2953, 2930, 2884, 2857, 1718, 1593, 1518, 1462, 1441, 1407, 1361, 1341, 1305, 1252, 1198, 1163, 1113, 1064, 1050, 1034, 1007, 939, 907, 872, 837, 802, 777, 736, 671, 667 cm^{-1}. HRMS (ESI): *m/z* calcd. for $C_{19}H_{30}O_5SiNa$ [M+Na$^+$]: 389.1754, found: 389.1758.

Methyl 2-((1*S*,2*S*,5*R*,6*S*,7*R*)-7-acetoxy-2-((*tert*-butyldimethylsilyl)oxy)bicyclo[3.2.0]heptan-6-yl)furan-3-carboxylate (S8).

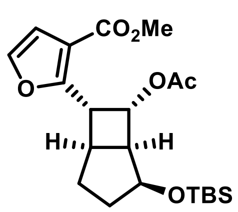

4-Dimethylaminopyridine (7 mg, 0.058 mmol), triethylamine (0.28 mL, 2.04 mmol) and acetic anhydride (0.19 mL, 2.04 mmol) were added to a stirred solution of alcohol **S7** (249 mg, 0.68 mmol) in CH_2Cl_2 (6 mL) at 0 °C. The mixture was stirred at room temperature for 1 h before the reaction was quenched upon addition of sat. aq. $NaHCO_3$ (5 mL). The aqueous phase was extracted with CH_2Cl_2 (3 x 50 mL), the combined organic layers were dried over Na_2SO_4 and concentrated, and the residue was purified by flash chromatography on silica (hexanes:*tert*-butyl methyl ether, 9:1) to provide the title compound as a colorless oil (262 mg, 94% yield). $[\alpha]_{\mathrm{D}}^{20}$ = –67.0° (*c* = 1.0, $CHCl_3$); ^1H NMR (400 MHz, $CDCl_3$): δ = 7.35 (d, *J* = 1.9 Hz, 1H), 6.67 (d, *J* = 1.9 Hz, 1H), 5.46 (dd, *J* = 7.8, 3.7 Hz, 1H), 4.29 – 4.20 (m, 2H), 3.81 (s, 3H), 3.16 – 3.07 (m, 1H), 2.87 (tdd, *J* = 8.0, 3.7, 1.4 Hz, 1H), 2.00 – 1.84 (m, 2H), 1.73 (s, 3H), 1.66 (td, *J* = 6.1, 3.8 Hz, 2H), 0.86 (s, 9H), 0.04 (d, *J* = 12.7 Hz, 6H); ^{13}C NMR (101 MHz, $CDCl_3$): δ = 169.4, 163.9, 159.1, 141.0, 114.6, 111.0, 73.8, 68.0, 51.4, 47.1, 42.3, 36.8, 33.3, 27.9, 25.7, 20.3, 18.1, –4.9, –5.0; IR (film): $\tilde{\nu}$ = 2954, 2931, 2857, 1746, 1722, 1598, 1518, 1463, 1443, 1407, 1362, 1340, 1304, 1284, 1232, 1197, 1160, 1133, 1115, 1054, 1033, 938, 892, 872, 837, 805, 777, 739, 669, 603 cm^{-1}. HRMS (ESI): *m/z* calcd. for $C_{21}H_{32}O_6SiNa$ $[M+Na^+]$: 431.1860, found: 431.1861.

Methyl 2-((1*R*,2*S*,5*R*,6*S*,7*R*)-7-acetoxy-2-hydroxybicyclo[3.2.0]heptan-6-yl)furan-3-carboxylate (S9).

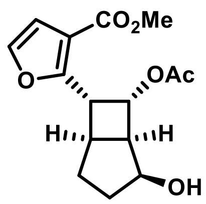

Tetrabutylammonium fluoride (1 M in THF, 0.72 mL, 0.72 mmol) was added to a stirred solution of silyl ether **S8** (246 mg, 0.60 mmol) in THF (6 mL) at 0 °C. The resulting mixture was stirred at room temperature for 5 h before the reaction was quenched with sat. aq. NH_4Cl (5 mL). The aqueous phase was extracted with CH_2Cl_2 (3 x 50 mL), the combined organic layers were dried over Na_2SO_4 and concentrated, and the residue was purified by flash chromatography on silica (hexanes:EtOAc, 1:1) to afford the title compound as a yellow oil (142 mg, 80% yield). $[\alpha]_{\mathrm{D}}^{20}$ = –134.1° (*c* = 1.0, $CHCl_3$); ^1H NMR (400 MHz, $CDCl_3$): δ = 7.37 (d, *J* = 2.0 Hz, 1H), 6.70 (d, *J* = 2.0 Hz, 1H), 5.22 (dd, *J* = 8.6, 4.3 Hz, 1H), 4.29 – 4.19 (m, 2H), 3.81 (s, 3H), 3.19 – 3.10 (m, 1H), 2.97 – 2.89 (m, 1H), 2.17 – 2.09 (m, 1H), 1.94 – 1.84 (m, 4H), 1.83 – 1.64 (m, 2H); ^{13}C NMR (101 MHz, $CDCl_3$): δ = 171.5, 164.2, 158.7, 141.2, 114.7, 110.8, 73.6, 68.0, 51.4, 49.1, 40.9, 36.6, 33.1, 28.1, 20.9; IR (film): $\tilde{\nu}$ = 3467, 2954, 2869, 1819, 1717, 1596, 1518, 1442, 1405, 1376, 1338, 1304, 1235, 1197, 1160, 1133, 1085, 1053, 1034, 942, 886, 805, 752, 604 cm^{-1}. HRMS (ESI): *m/z* calcd. for $C_{15}H_{18}O_6Na$ $[M+Na^+]$: 317.0995, found: 317.0993.

Methyl 2-((1*S*,5*R*,6*S*,7*R*)-7-acetoxybicyclo[3.2.0]hept-2-en-6-yl)furan-3-carboxylate (*ent*-21). A

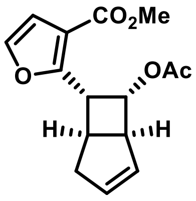

solution of Martin's sulfurane (428 mg, 0.64 mmol) in CH_2Cl_2 (2 mL) was added to a stirred solution of alcohol **S9** (125 g, 0.42 mmol) in CH_2Cl_2 (4 mL). The resulting mixture was stirred for 5 h before the reaction was quenched with sat. aq. $NaHCO_3$ (4 mL). The aqueous phase was extracted with CH_2Cl_2 (3 x 50 mL), the combined extracts were dried over Na_2SO_4 and concentrated, and the residue was purified by flash chromatography on silica (hexanes:*tert*-butyl methyl ether, 8:1) to provide the title compound as a yellow oil (94 mg, 80% yield). $[\alpha]_D^{20}$ = –47.7° (*c* = 1.0, $CHCl_3$); ^1H NMR (400 MHz, $CDCl_3$): δ = 7.36 (d, *J* = 2.0 Hz, 1H), 6.68 (d, *J* = 2.0 Hz, 1H), 5.88 (s, 2H), 5.11 (dd, *J* = 6.7, 1.5 Hz, 1H), 4.22 (td, *J* = 7.1, 1.5 Hz, 1H), 3.79 (s, 3H), 3.62 – 3.56 (m, 1H), 3.34 – 3.28 (m, 1H), 2.70 – 2.57 (m, 1H), 2.36 (ddd, *J* = 17.3, 3.7, 1.3 Hz, 1H), 1.86 (s, 3H); ^{13}C NMR (101 MHz, $CDCl_3$): δ = 170.0, 164.0, 158.7, 141.1, 133.0, 129.7, 114.1, 111.0, 76.6, 51.5, 51.4, 42.5, 39.4, 36.9, 20.7; IR (film): $\tilde{\nu}$ = 2952, 2845, 1737, 1715, 1595, 1516, 1440, 1409, 1372, 1353, 1330, 1292, 1230, 1195, 1164, 1140, 1106, 1066, 1032, 950, 915, 876, 831, 860, 799, 751, 721, 603 cm^{-1}. HRMS (ESI): *m/z* calcd. for $C_{15}H_{20}NO_5$ $[M+NH_4^+]$: 294.1336, found: 294.1337.

Methyl 2-((1*R*,2*S*,3*S*,4*S*)-2-acetoxy-4-(2-hydroxyethyl)-3-(hydroxymethyl)cyclobutyl)furan-3-carboxylate (22).

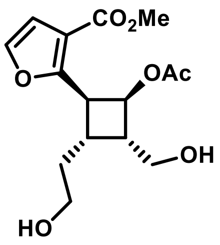

N-Methylmorpholine *N*-oxide (2.09 g, 17.92 mmol) and OsO_4 (4 wt% in water, 0.76 mL, 0.12 mmol, 0.01 eq.) were added to a stirred solution of olefin **21** (1.65 g, 5.97 mmol) in a mixture (10:1 *v/v*, 30 mL) of THF/H_2O at room temperature. The mixture was stirred for 3 d before sat. aq. Na_2SO_3 (10 mL) and *tert*-butyl methyl ether (50 mL) were added. The aqueous phase was extracted with *tert*-butyl methyl ether (3 x 150 mL), the combined extracts were washed with brine (50 mL), dried over Na_2SO_4 and concentrated, and the resulting crude diol was used in the next step without further purification.

Sodium periodate (1.53 g, 7.17 mmol) was added to a stirred solution of this diol (1.85 g, 5.97 mmol) in THF (38 mL) and water (8 mL). The resulting mixture was vigorously stirred at room temperature for 30 min before it was cooled to 0 °C. Methanol (115 mL) was added and stirring continued for 15 min at 0 °C. Sodium borohydride (904 mg, 23.89 mmol) was introduced and the mixture stirred for 30 min at 0 °C. The mixture was poured into a mixture of sat. aq. NH_4Cl (50 mL) and EtOAc (100 mL). After vigorous stirring for 30 min, the aqueous phase was extracted with EtOAc (3 x 250 mL). The combined extracts were washed with sat. aq. $Na_2S_2O_3$ (50 mL) and brine (50 mL) before they were concentrated *in vacuo*. The residue was purified by flash chromatography on silica (CH_2Cl_2:MeOH, 95:5) to give the title compound as a colorless oil (1.44 g, 77% yield over 2 steps). $[\alpha]_D^{20}$ = +55.2° (*c* = 0.5, $CHCl_3$); ^1H NMR (400 MHz, $CDCl_3$): δ = 7.34 (d, *J* = 2.0 Hz, 1H), 6.68 (d, *J* = 2.0 Hz, 1H), 5.10 (ddd, *J* = 8.0, 5.4, 1.0 Hz, 1H), 4.30 (ddd, *J* = 8.0, 6.6, 1.1 Hz, 1H), 3.85 (d, *J* = 7.1 Hz, 2H), 3.80 (s, 3H), 3.70 (ddd, *J* = 10.9, 6.0, 5.0 Hz,

1H), 3.58 (ddd, *J* = 10.6, 8.4, 5.3 Hz, 1H), 3.15 – 3.03 (m, 1H), 2.90 (dtdd, *J* = 10.1, 7.0, 5.4, 1.1 Hz, 1H), 2.69 (s, 2H), 2.04 – 1.93 (m, 1H), 1.87 (s, 3H), 1.72 (ddt, *J* = 13.7, 8.4, 5.2 Hz, 1H); ^{13}C NMR (101 MHz, $CDCl_3$): δ = 171.0, 164.1, 158.6, 141.3, 114.6, 110.9, 71.9, 61.6, 60.6, 51.5, 45.3, 40.8, 33.9, 32.4, 20.7; IR (film): $\tilde{\nu}$ = 3403, 2952, 2875, 1717, 1596, 1518, 1442, 1408, 1372, 1309, 1286, 1236, 1199, 1161, 1134, 1108, 1046, 751, 604 cm^{-1}. HRMS (ESI): *m/z* calcd. for $C_{15}H_{20}O_7Na$ [M+Na$^+$]: 335.1101, found: 335.1101.

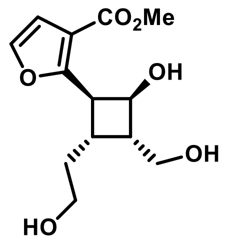

Methyl 2-((1*R*,2*S*,3*S*,4*S*)-2-hydroxy-4-(2-hydroxyethyl)-3-(hydroxymethyl)cyclobutyl)furan-3-carboxylate (S10). Acetyl chloride (0.27 mL, 3.84 mmol) was added to a stirred solution of diol **22** (400 mg, 1.28 mmol) in methanol (13 mL). The resulting mixture was stirred for 4 h at room temperature before it was concentrated *in vacuo*. The residue was purified by flash chromatography on silica (CH_2Cl_2:MeOH, 90:10) to afford the title compound as a colorless oil (234 mg, 67% yield). $[\alpha]_D^{20}$ = +45.9° (*c* = 1.0, $CHCl_3$); ^{1}H NMR (400 MHz, $CDCl_3$): δ = 7.35 (d, *J* = 2.0 Hz, 1H), 6.68 (d, *J* = 2.0 Hz, 1H), 4.37 (dd, *J* = 7.6, 5.3 Hz, 1H), 3.99 (t, *J* = 7.0 Hz, 1H), 3.90 – 3.77 (m, 5H), 3.72 (dt, *J* = 10.5, 5.2 Hz, 1H), 3.60 (ddd, *J* = 10.5, 8.6, 5.0 Hz, 1H), 3.15 (s, 3H), 3.07 – 2.96 (m, 1H), 2.74 (tt, *J* = 9.2, 5.7 Hz, 1H), 1.98 (ddt, *J* = 14.0, 8.2, 4.0 Hz, 1H), 1.69 (ddt, *J* = 13.9, 7.4, 5.0 Hz, 1H); ^{13}C NMR (101 MHz, $CDCl_3$): δ = 164.8, 160.2, 141.4, 114.4, 110.9, 70.3, 61.9, 60.8, 51.6, 47.5, 43.5, 33.2, 32.5; IR (film): $\tilde{\nu}$ = 3366, 2949, 2878, 1712, 1592, 1519, 1442, 1409, 1310, 1257, 1200, 1162, 1132, 1088, 1033, 740, 603 cm^{-1}. HRMS (ESI): *m/z* calcd. for $C_{13}H_{18}O_6Na$ [M+Na$^+$]: 293.0995, found: 293.0995.

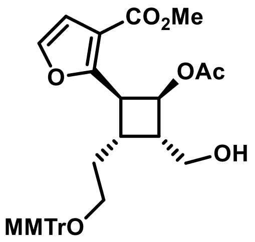

Methyl 2-((1*R*,2*S*,3*S*,4*S*)-2-acetoxy-3-(hydroxymethyl)-4-(2-((4-methoxyphenyl)diphenylmethoxy)-ethyl)cyclobutyl)furan-3-carboxylate (23). 4-Dimethylaminopyridine (22 mg, 0.18 mmol, 0.05 eq.) and pyridine (0.23 mL, 2.83 mmol, 0.8 eq.) were added to a stirred solution of diol **22** (1.10 g, 3.54 mmol) in CH_2Cl_2 (31 mL). The mixture was cooled to –42 °C using an acetonitrile/dry-ice cooling bath before a solution of 4-monomethoxytrityl chloride (765 mg, 2.48 mmol) in CH_2Cl_2 (6 mL) was added dropwise. Stirring was continued for 2 h at –42 °C before the reaction was quenched with sat. aq. NH_4Cl (10 mL). After reaching room temperature, the aqueous phase was extracted with CH_2Cl_2 (3 x 100 mL), the combined extracts were dried over $MgSO_4$ and concentrated, and the residue was purified by flash chromatography on silica (hexanes:EtOAc, 2:1 to pure EtOAc) to furnish the title compound as a white foam (950 mg, 46% yield), undesired mono-protected product **S11** as a white foam (144 mg, 7% yield), bis-protected product as a yellow oil **S12** (165 mg, 5% yield), and recovered starting material **22** as a colorless oil (355 mg, 32% yield).

Analytical and spectroscopic data of **23**: $[\alpha]_D^{20}$ = –23.2° (*c* = 1.0, MeOH); ^{1}H NMR (400 MHz, CD_2Cl_2): δ = 7.33 (dtd, *J* = 5.9, 3.2, 1.5 Hz, 5H), 7.27 – 7.15 (m, 8H), 6.79 – 6.73 (m, 2H), 6.66 (d, *J* = 2.0 Hz, 1H),

5.10 (ddd, J = 8.3, 5.5, 1.0 Hz, 1H), 4.23 (ddd, J = 8.0, 6.4, 1.3 Hz, 1H), 3.85 – 3.73 (m, 5H), 3.71 (s, 3H), 3.27 – 3.18 (m, 1H), 3.07 – 2.93 (m, 2H), 2.88 – 2.76 (m, 1H), 2.19 (dd, J = 6.8, 4.3 Hz, 1H), 2.01 – 1.91 (m, 1H), 1.84 (s, 3H), 1.72 (ddt, J = 13.8, 10.9, 5.4 Hz, 1H); ^{13}C NMR (101 MHz, CD_2Cl_2): δ = 171.5, 164.1, 159.0, 158.9, 145.2, 145.1, 141.6, 136.1, 130.6, 128.7, 128.6, 128.1, 127.09, 127.07, 115.0, 113.3, 111.2, 86.5, 72.6, 61.9, 61.3, 55.5, 51.5, 45.8, 41.0, 33.5, 30.6, 21.0; IR (film): $\tilde{\nu}$ = 3502, 2950, 2872, 2838, 1717, 1604, 1510, 1490, 1445, 1412, 1371, 1301, 1248, 1197, 1179, 1158, 1133, 1113, 1061, 1033, 954, 902, 831, 796, 766, 749, 728, 708, 633, 603, 586, 545 cm^{-1}. HRMS (ESI): m/z calcd. for $C_{35}H_{36}O_8Na$ [M+Na$^+$]: 607.2302, found: 607.2307.

Analytical and spectroscopic data of **S11**: $[\alpha]_D^{20}$ = –14.3° (c = 1.0, MeOH); ^{1}H NMR (400 MHz, CD_2Cl_2): δ

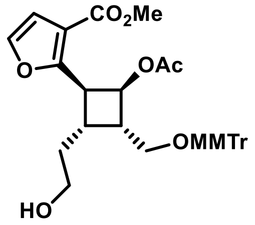

= 7.47 (dt, J = 8.2, 1.3 Hz, 4H), 7.39 (d, J = 2.0 Hz, 1H), 7.37 – 7.29 (m, 6H), 7.27 – 7.16 (m, 2H), 6.89 – 6.84 (m, 2H), 6.68 (d, J = 2.0 Hz, 1H), 5.27 (dd, J = 7.8, 4.7 Hz, 1H), 4.30 (dd, J = 7.8, 5.1 Hz, 1H), 3.80 (s, 3H), 3.77 (s, 3H), 3.52 – 3.37 (m, 2H), 3.32 – 3.27 (m, 2H), 3.08 – 3.00 (m, 2H), 1.83 (s, 3H), 1.76 – 1.66 (m, 1H), 1.57 – 1.50 (m, 1H); ^{13}C NMR (101 MHz, CD_2Cl_2): δ = 170.1, 164.3, 159.4, 159.1, 145.1, 145.0, 141.7, 135.9, 130.8, 128.8, 128.24, 128.22, 127.3, 114.9, 113.5, 111.2, 86.9, 71.8, 61.7, 61.3, 55.6, 51.7, 43.0, 41.9, 33.8, 33.4, 20.8; IR (film): $\tilde{\nu}$ = 3420, 2949, 2909, 2868, 2837, 1737, 1717, 1598, 1509, 1490, 1444, 1411, 1371, 1300, 1233, 1197, 1179, 1155, 1133, 1114, 1071, 1032, 901, 832, 795, 748, 728, 708, 669, 632, 592, 546 cm^{-1}. HRMS (ESI): m/z calcd. for $C_{35}H_{36}O_8Na$ [M+Na$^+$]: 607.2302, found: 607.2305.

Analytical and spectroscopic data of **S12**: $[\alpha]_D^{20}$ = –16.2° (c = 1.0, MeOH); ^{1}H NMR (400 MHz, CD_2Cl_2): δ

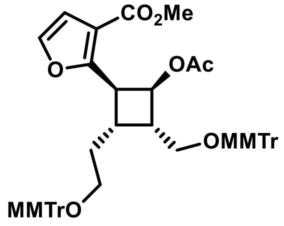

= 7.46 (dq, J = 6.7, 1.2 Hz, 4H), 7.35 (d, J = 1.9 Hz, 1H), 7.34 – 7.27 (m, 10H), 7.26 – 7.12 (m, 10H), 6.86 – 6.81 (m, 2H), 6.75 – 6.70 (m, 2H), 6.67 (d, J = 2.0 Hz, 1H), 5.27 (ddd, J = 7.9, 5.9, 1.0 Hz, 1H), 4.21 (ddd, J = 8.0, 6.1, 1.1 Hz, 1H), 3.78 (s, 3H), 3.76 (s, 3H), 3.69 (s, 3H), 3.25 (d, J = 6.7 Hz, 2H), 3.20 (dq, J = 10.7, 5.4 Hz, 1H), 3.06 – 2.96 (m, 1H), 2.95 – 2.82 (m, 2H), 1.82 (m, 4H), 1.54 (m, 1H); ^{13}C NMR (101 MHz, CD_2Cl_2): δ = 170.0, 164.1, 159.3, 159.1, 158.9, 145.3, 145.2, 145.13, 145.07, 141.5, 136.2, 136.0, 130.8, 130.5, 128.8, 128.7, 128.6, 128.2, 128.0, 127.2, 127.04, 127.01, 115.0, 113.4, 113.2, 111.3, 86.8, 86.4, 78.0, 71.7, 61.9, 61.8, 55.6, 55.5, 51.5, 42.9, 42.0, 33.6, 30.7, 20.8; IR (film): $\tilde{\nu}$ = 2972, 2908, 2870, 1741, 1720, 1606, 1510, 1491, 1463, 1446, 1412, 1364, 1300, 1250, 1232, 1200,1180, 1155, 1134, 1115, 1080, 1034, 989, 936, 901, 850, 831, 796, 766, 748, 727, 707, 672, 665, 633, 614, 586, 545, 464 cm^{-1}. HRMS (ESI): m/z calcd. for $C_{55}H_{52}O_9Na$ [M+Na$^+$]: 879.3503, found: 879.3500.

Recycling of S11+S12 to Diol 22. Pyridinium p-toluenesulfonate (10 mg, 0.04 mmol, 0.1 eq.) was added to a stirred solution of **S11** (119 mg, 0.20 mmol) and **S12** (165 mg, 0.19 mmol) in CH_2Cl_2/MeOH (4:1

v/v, 4 mL) at room temperature. The resulting mixture was stirred for 4 h, before the reaction was quenched with sat. aq. $NaHCO_3$ (2 mL) and water (2 mL). The aqueous phase was extracted with Et_2O (3 x 50 mL). The combined organic extracts were dried over $MgSO_4$ and concentrated, and the residue was purified by flash chromatography on silica (EtOAc) to give product **22** as a colorless oil (99 mg, 80% yield).

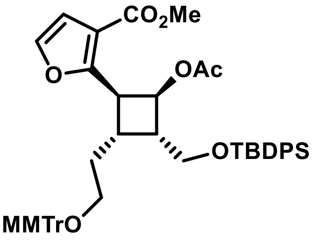

Methyl 2-((1*R*,2*S*,3*S*,4*S*)-2-acetoxy-3-(((*tert*-butyldiphenylsilyl)oxy)methyl)-4-(2-((4-methoxyphenyl)diphenylmethoxy)ethyl)cyclobutyl)furan-3-carboxylate (S13). 4-Dimethylaminopyridine (9 mg, 0.07 mmol, 0.05 eq.), triethylamine (1.4 mL, 10.06 mmol) and *tert*-butyldiphenylsilyl chloride (0.56 mL, 2.16 mmol) were added to a stirred solution of alcohol **23** (840 mg, 1.44 mmol) in CH_2Cl_2 (17 mL). The resulting mixture was stirred for 2 d at room temperature before the reaction was quenched with sat. aq. $NaHCO_3$ (10 mL). The mixture was diluted with CH_2Cl_2 (20 mL), the aqueous phase was extracted with CH_2Cl_2 (3 x 100 mL), the combined organic layers were dried over $MgSO_4$ and concentrated, and the residue was purified by flash chromatography on silica (hexanes:EtOAc, 4:1) to provide the title compound as a colorless oil (1.10 g, 93% yield). $[\alpha]_D^{20}$ = –8.1° (*c* = 1.0, MeOH); ^1H NMR (400 MHz, CD_2Cl_2): δ = 7.69 (dt, *J* = 8.1, 1.6 Hz, 4H), 7.47 – 7.37 (m, 6H), 7.32 (ddt, *J* = 4.6, 3.2, 1.6 Hz, 5H), 7.25 – 7.13 (m, 8H), 6.77 – 6.72 (m, 2H), 6.66 (d, *J* = 2.0 Hz, 1H), 5.31 – 5.27 (m, 1H), 4.24 (ddd, *J* = 7.9, 6.5, 1.1 Hz, 1H), 3.87 (qd, *J* = 10.7, 6.2 Hz, 2H), 3.76 (s, 3H), 3.69 (s, 3H), 3.29 – 3.19 (m, 1H), 3.05 – 2.85 (m, 3H), 2.10 – 1.99 (m, 1H), 1.82 (h, *J* = 5.5 Hz, 1H), 1.78 (s, 3H), 1.08 (s, 9H); ^{13}C NMR (101 MHz, CD_2Cl_2): δ = 170.0, 164.1, 159.2, 158.9, 145.4, 145.2, 141.5, 136.3, 136.1, 136.0, 134.0, 133.9, 130.6, 130.1, 128.7, 128.6, 128.1, 128.0, 127.1, 127.0, 115.0, 113.3, 111.3, 86.4, 71.4, 62.4, 61.9, 55.5, 51.5, 44.5, 41.8, 33.9, 30.9, 27.1, 20.7, 19.5; IR (film): $\tilde{\nu} = 3069$, 2952, 2931, 2858, 1740, 1719, 1603, 1510, 1489, 1463, 1445, 1428, 1412, 1390, 1372, 1302, 1233, 1195, 1180, 1156, 1112, 1089, 1066, 1034, 954, 901, 826, 796, 766, 743, 704, 632, 613, 586, 505 cm^{-1}. HRMS (ESI): *m/z* calcd. for $C_{51}H_{54}O_8SiNa$ [M+Na$^+$]: 845.3480, found: 845.3474.

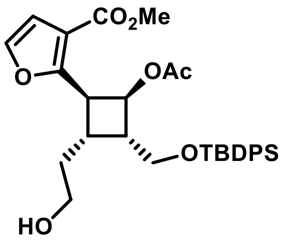

Methyl 2-((1*R*,2*S*,3*S*,4*S*)-2-acetoxy-3-(((*tert*-butyldiphenylsilyl)oxy)methyl)-4-(2-hydroxyethyl)cyclobutyl)furan-3-carboxylate (24). Pyridinium *p*-toluenesulfonate (33 mg, 0.13 mmol, 0.1 eq.) was added to a stirred solution of furan **S13** (1.09 g, 1.32 mmol) in a mixture of CH_2Cl_2/MeOH (4:1 *v/v*, 27 mL). The solution was stirred for 5 h at room temperature before the reaction was quenched upon addition of sat. aq. $NaHCO_3$ (10 mL). The mixture was diluted with water (10 mL), the aqueous phase was extracted with Et_2O (3 x 100 mL), the combined extracts were dried over $MgSO_4$ and concentrated, and the residue was purified by flash chromatography on silica (hexanes:EtOAc, 3:1) to give the title compound as a colorless oil (683 mg, 94% yield). $[\alpha]_D^{20}$ = –3.6° (*c*

= 1.0, $CHCl_3$); ^{1}H NMR (400 MHz, $CDCl_3$): δ = 7.72 – 7.64 (m, 4H), 7.47 – 7.36 (m, 6H), 7.34 (d, *J* = 1.9 Hz, 1H), 6.68 (d, *J* = 1.9 Hz, 1H), 5.29 (dd, *J* = 7.7, 5.5 Hz, 1H), 4.35 – 4.29 (m, 1H), 3.94 – 3.81 (m, 2H), 3.79 (s, 3H), 3.67 – 3.52 (m, 2H), 3.11 (tt, *J* = 10.0, 6.3 Hz, 1H), 2.95 – 2.85 (m, 1H), 1.97 (ddt, *J* = 13.8, 7.6, 6.2 Hz, 1H), 1.82 (s, 3H), 1.80 – 1.73 (m, 1H), 1.08 (s, 9H); ^{13}C NMR (101 MHz, $CDCl_3$): δ = 169.8, 164.0, 158.8, 141.1, 135.7, 133.3, 133.2, 129.8, 127.7, 114.6, 111.0, 71.2, 61.9, 61.3, 51.5, 44.1, 41.4, 33.7, 33.0, 26.9, 20.5, 19.2; IR (film): $\tilde{\nu}$ = 3466, 2953, 2932, 2859, 1740, 1720, 1597, 1518, 1472, 1443, 1428, 1391, 1372, 1307, 1284, 1235, 1197, 1160, 1133, 1111, 1087, 1048, 939, 823, 799, 742, 704, 612, 505, 491 cm^{-1}. HRMS (ESI): *m/z* calcd. for $C_{31}H_{38}O_7SiNa$ [M+Na$^+$]: 573.2279, found: 573.2277.

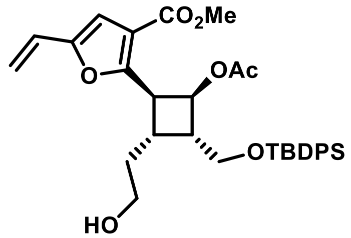

Methyl 2-((1*R*,2*S*,3*S*,4*S*)-2-acetoxy-3-(((*tert*-butyldiphenylsilyl)oxy)methyl)-4-(2-hydroxyethyl)cyclobutyl)-5-vinylfuran-3-carboxylate (3). Lithium carbonate (2 mg, 0.03 mmol), AgOMs (12 mg, 0.06 mmol) and iodine (15 mg, 0.06 mmol) were successively added to a stirred solution of furan **24** (15 mg, 0.03 mmol) in acetonitrile (0.4 mL) at room temperature. The resulting mixture was stirred for 10 min before it was diluted with EtOAc (10 mL) and sat. aq. $Na_2S_2O_3$/$NaHCO_3$ (1:1 *v/v*, 10 mL). The biphasic mixture was vigorously stirred for 10 min until full decolorization and solvation of the precipitate initially formed was reached. The aqueous phase was diluted with water (5 mL) and extracted with EtOAc (3 x 20 mL). The combined organic layers were washed with brine (30 mL), dried over $MgSO_4$ and concentrated. The crude iodofuran **25** thus formed was used in the next step without further purification.

The commercial palladium complex XPhos-Pd-G2 (**28**) (3 mg, 0.004 mmol) was added to a stirred solution of iodofuran **25** (18 mg, 0.027 mmol) and potassium (ethenyl)trifluoroborate (**26**) (7 mg, 0.05 mmol) in a mixture of THF (1 mL) and aqueous K_3PO_4 (3 M, 0.1 mL). The resulting mixture was stirred for 6 h at 55 °C (oil bath) before Et_2O (20 mL) and water (5 mL) were added. The aqueous phase was extracted with Et_2O (3 x 70 mL), the combined organic layers were washed with brine (10 mL), dried over $MgSO_4$ and concentrated. The residue was purified by flash chromatography on silica (hexanes:EtOAc, 3:1) to afford the title compound as a brown oil (6.8 mg, 44% yield over 2 steps). $[\alpha]_D^{20}$ = –84.6° (*c* = 0.5, $CHCl_3$); ^{1}H NMR (400 MHz, CD_2Cl_2): δ = 7.72 – 7.67 (m, 4H), 7.48 – 7.38 (m, 6H), 6.54 – 6.44 (m, 2H), 5.76 – 5.68 (m, 1H), 5.30 – 5.21 (m, 2H), 4.32 (ddd, *J* = 7.8, 6.7, 1.1 Hz, 1H), 3.98 – 3.83 (m, 2H), 3.76 (s, 3H), 3.64 – 3.49 (m, 2H), 3.11 (tddd, *J* = 9.9, 6.6, 5.7, 1.0 Hz, 1H), 2.98 – 2.87 (m, 1H), 2.02 – 1.91 (m, 1H), 1.85 – 1.73 (m, 4H), 1.08 (s, 9H); ^{13}C NMR (101 MHz, CD_2Cl_2): δ = 170.2, 164.2, 158.8, 152.0, 136.1, 136.0, 133.9, 133.8, 130.1, 128.1, 124.8, 116.3, 113.4, 108.9, 71.5, 62.4, 61.5, 51.7, 44.6, 41.9, 34.5, 33.6, 27.0, 20.8, 19.5; IR (film): $\tilde{\nu}$ = 3463, 3071, 2952, 2932, 2858, 1740, 1719, 1643, 1591, 1538, 1472, 1442, 1428, 1411, 1373, 1298, 1229, 1111, 1068, 980, 939, 906, 823, 781, 741, 703, 611, 505, 491 cm^{-1}. HRMS (ESI): *m/z* calcd. for $C_{33}H_{40}O_7SiNa$ [M+Na$^+$]: 599.2435, found: 599.2435.

Attempted RCAM

(*E*)-4-Iodo-3-methylbut-3-en-1-ol (30). This compound was prepared according to the literature procedure.[3] ^{1}H NMR (400 MHz, $CDCl_3$): δ = 6.02 (q, *J* = 1.2 Hz, 1H), 3.72 (t, *J* = 6.3 Hz, 2H), 2.48 (td, *J* = 6.3, 1.1 Hz, 2H), 1.88 (d, *J* = 1.1 Hz, 3H); ^{13}C NMR (101 MHz, $CDCl_3$): δ = 144.6, 76.9, 60.1, 42.4, 23.8; HRMS (ESI): *m/z* calcd. for C_5H_9OI [M^+]: 211.9692, found: 211.9694.

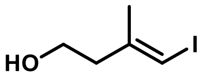

(*S,E*)-1-Iodo-2-methylhept-1-en-5-yn-4-ol (31). Sodium bicarbonate (5.35 g, 63.67 mmol) and Dess-Martin periodinane (6.75 g, 15.92 mmol) were added to a solution of alcohol **30** (1.35 g, 6.37 mmol) in CH_2Cl_2 (50 mL) at 0 °C. The resulting mixture was stirred at room temperature for 1 h before it was cooled to 0 °C and a mixture of sat. aq. $Na_2S_2O_3$/$NaHCO_3$ (1:1 *v/v*, 50 mL) was added. The resulting mixture was vigorously stirred for 30 min before it was diluted with water (100 mL) and extracted with CH_2Cl_2 (3 x 150 mL). The combined organic layers were washed with sat. aq. $NaHCO_3$ (3 x 150 mL), dried over $MgSO_4$ and concentrated *in vacuo* (Caution: the aldehyde is volatile! The temperature must be kept at 20 °C and the pressure > 250 mbar). The crude aldehyde was used in the next step without further purification.

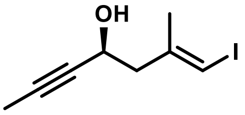

Triethylamine (1.85 mL, 13.29 mmol) was added to a vigorously stirred suspension of $Zn(OTf)_2$ (4.60 g, 12.65 mmol) and (1*R*,2*S*)-(–)-*N*-methylephedrine (2.38 g, 13.29 mmol) in toluene (43 mL). The resulting mixture was stirred for 2 h at room temperature before it was cooled to 0 °C and liquid propyne (4 mL, 98.7 mmol) was added via cannula. After stirring for another 45 min at room temperature, a solution of the crude aldehyde (1.33 g, 6.33 mmol) in toluene (6 mL) was slowly added over the course of 4 h. Once the addition was complete, stirring was continued for 16 h before the reaction was quenched with sat. aq. NH_4Cl (30 mL). The mixture was diluted with water (30 mL), the aqueous phase was extracted with Et_2O (3 x 150 mL), the combined extracts were washed with brine (50 mL), dried over $MgSO_4$ and concentrated (T ≥ 20 °C; the compound is heat sensitive!), and the residue was purified by flash chromatography on silica (pentane:Et_2O, 4:1) to provide the title compound as a yellow oil (512 mg, 32% yield, 88% ee). The characterization data are in accordance with the literature.[4] $[\alpha]_D^{20}$ = –12.9° (*c* = 1.0, $CHCl_3$); ^{1}H NMR (400 MHz, $CDCl_3$): δ = 6.08 (q, *J* = 1.1 Hz, 1H), 4.48 – 4.43 (m, 1H), 2.64 – 2.51 (m, 2H), 1.91 (d, *J* = 1.1 Hz, 3H), 1.84 (d, *J* = 2.1 Hz, 3H). ^{13}C NMR (101 MHz, $CDCl_3$): δ = 143.5, 81.9, 79.4, 78.4, 60.6, 47.6, 24.3, 3.5. HRMS (ESI): *m/z* calcd. for $C_8H_{12}OI$ [$M+H^+$]: 250.9927, found: 250.9927.

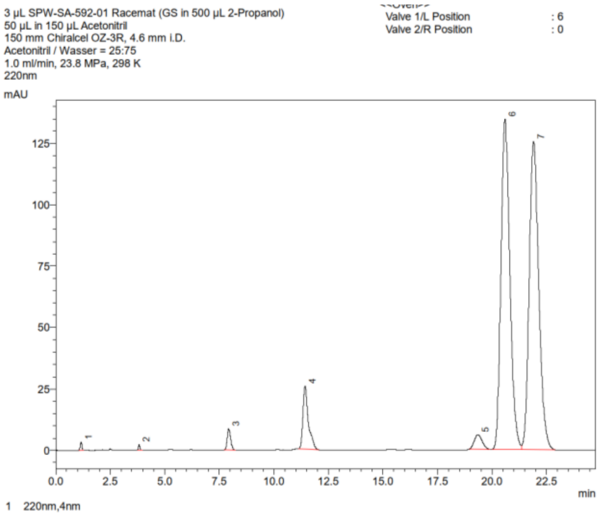

PDA Ch1 220nm

Peak #	Ret. Time	Area %	Name	Separation Factor	Resolution(USP)
1	1.16	0.21		--	--
2	3.82	0.13		--	20.36
3	7.93	1.14		2.55	20.00
4	11.43	5.33		1.52	10.27
5	19.36	1.83	1. Isomer	1.77	14.54
6	20.60	45.64	2. Isomer/1. Enantiomer	1.07	1.73
7	21.91	45.72	2. Isomer/2. Enantiomer	1.07	1.70
Total		100.00			

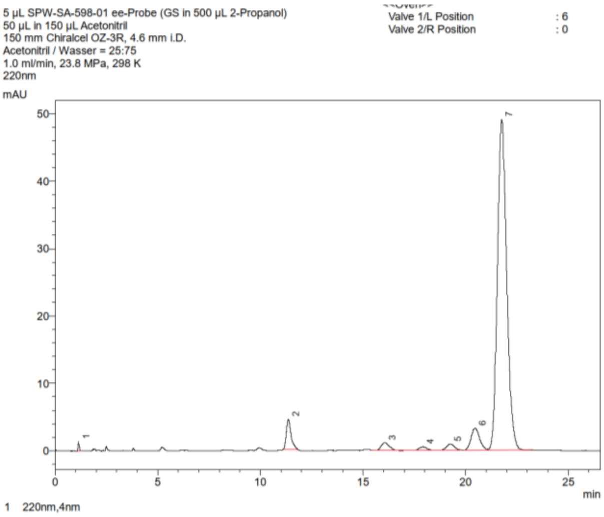

PDA Ch1 220nm

Peak #	Ret. Time	Area %	Name	
1	1.15	0.33		
2	11.36	4.09		
3	16.06	1.67		
4	17.92	0.73		
5	19.25	1.37	1. Isomer	
6	20.46	5.32	2. Isomer/1. Enantiomer	ee = 88.4%
7	21.75	86.49	2. Isomer/2. Enantiomer	
Total		100.00		

Figure S3. HPLC-traces of rac-**31** (left) and enantioenriched **31** (right): t_R = 20.46 min (minor enantiomer) and 21.75 min (major enantiomer) (Chiralcel OZ-3R column, λ = 220 nm, isocratic elution 25:75 acetonitrile/water, flow-rate = 1.0 mL/min).

(*S*,*E*)-1-Iodo-4-(methoxymethoxy)-2-methylhept-1-en-5-yne (32). *N*,*N*-Diisopropylethylamine (0.94 mL, 5.39 mmol) and chloromethyl methyl ether (0.21 mL, 2.70 mmol) were added to a stirred solution of alcohol **31** (168 mg, 0.67 mmol) in CH_2Cl_2 (1.9 mL) at 0 °C. The resulting mixture was stirred at room temperature for 16 h before the reaction was quenched with sat. aq. $NaHCO_3$ (3 mL). The aqueous phase was extracted with CH_2Cl_2 (3 x 50 mL), the combined extracts were dried over $MgSO_4$ and concentrated, and the residue was purified by flash chromatography on silica (pentane:Et_2O, 60:1) to afford the title compound as a colorless oil (133 mg, 67% yield).

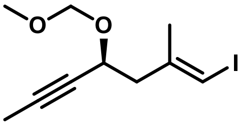

$[\alpha]_D^{20}$ = –57.8° (*c* = 0.5, $CHCl_3$); ^1H NMR (400 MHz, CD_2Cl_2): δ = 6.06 (q, *J* = 1.1 Hz, 1H), 4.88 (d, *J* = 6.8 Hz, 1H), 4.51 (dd, *J* = 6.8, 0.5 Hz, 1H), 4.43 – 4.36 (m, 1H), 3.30 (s, 3H), 2.66 – 2.50 (m, 2H), 1.89 (d, *J* = 1.1 Hz, 3H), 1.83 (d, *J* = 2.1 Hz, 3H); ^{13}C NMR (101 MHz, CD_2Cl_2): δ = 144.3, 94.2, 82.7, 78.1, 77.4, 64.3, 55.9, 45.8, 24.5, 3.6; IR (film): $\tilde{\nu}$ = 2949, 2918, 2888, 2849, 2822, 1439, 1377, 1346, 1277, 1226, 1148, 1096, 1060, 1027, 969, 947, 919, 761, 671 cm^{-1}. HRMS (ESI): *m/z* calcd. for $C_{10}H_{15}O_2INa$ [M+Na$^+$]: 317.0009, found: 317.0011.

(*S*,*E*)-2-(4-(Methoxymethoxy)-2-methylhept-1-en-5-yn-1-yl)-4,4,5,5-tetramethyl-1,3,2-dioxaborolane (33). *n*-BuLi (1.6 M in hexanes, 0.35 mL, 0.56 mmol) was added dropwise to a stirred solution of alkenyl iodide **32** (127 mg, 0.43 mmol) and triisopropyl borate (0.13 mL, 0.56 mmol) in a mixture of THF (0.7 mL) and

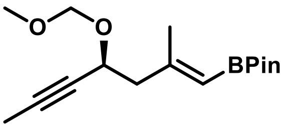

toluene (2.7 mL) at –78 °C. The resulting mixture was stirred for 40 min at –78 °C before pinacol (77 mg, 0.65 mmol) was added as a solid. The mixture was warmed to room temperature and stirring was continued for an additional 16 h. Et_2O (50 mL) was added and the organic phase was washed with sat. aq. NH_4Cl (10 mL), water (10 mL), brine (10 mL), dried over $MgSO_4$ and concentrated. The residue was purified by flash chromatography on silica (pentane:Et_2O, 20:1 to 10:1) to give the title compound as a colorless oil (107 mg, 84% yield). $[\alpha]_D^{20}$ = –97.4° (*c* = 0.5, $CHCl_3$); ^1H NMR (400 MHz, CD_2Cl_2): δ = 5.16 (q, *J* = 1.1 Hz, 1H), 4.89 (d, *J* = 6.7 Hz, 1H), 4.52 (dd, *J* = 6.8, 0.5 Hz, 1H), 4.43 (ddt, *J* = 9.4, 6.3, 2.1 Hz, 1H), 3.30 (s, 3H), 2.54 – 2.40 (m, 2H), 2.01 (d, *J* = 1.0 Hz, 3H), 1.83 (d, *J* = 2.1 Hz, 3H), 1.24 (s, 12H); ^{13}C NMR (101 MHz, CD_2Cl_2): δ = 158.0, 94.2, 83.0, 82.1, 78.0, 64.9, 55.8, 48.8, 25.0, 21.5, 3.6; IR (film): $\tilde{\nu}$ = 2978, 2923, 1640, 1441, 1401, 1370, 1350, 1319, 1283, 1262, 1214, 1144, 1098, 1064, 1030, 970, 919, 853 cm^{-1}. HRMS (ESI): *m/z* calcd. for $C_{16}H_{27}O_4BNa$ [M+Na$^+$]: 317.1894, found: 317.1896.

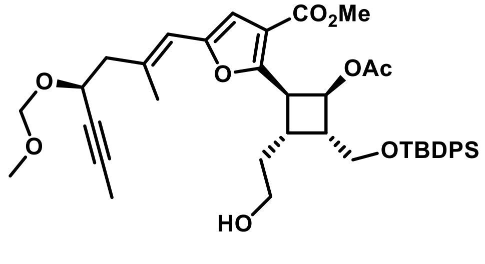

Methyl 2-((1*R*,2*S*,3*S*,4*S*)-2-acetoxy-3-(((*tert*-butyldiphenylsilyl)oxy)methyl)-4-(2-hydroxyethyl)cyclobutyl)-5-((*S*,*E*)-4-(methoxymethoxy)-2-methylhept-1-en-5-yn-1-yl)furan-3-carboxylate (34). Lithium carbonate (29 mg, 0.40 mmol), AgOMs (162 mg, 0.80 mmol) and iodine (203 mg, 0.80 mmol) were successively added to a stirred solution of furan **24** (200 mg, 0.36 mmol) in acetonitrile (4.8 mL) at room temperature. The resulting mixture was stirred for 10 min before EtOAc (10 mL) and a mixture of sat. aq. $Na_2S_2O_3$:$NaHCO_3$ (1:1 *v/v*, 10 mL) were added. The biphasic mixture was vigorously stirred for 10 min until decolorization and solvation of a precipitate was reached. The aqueous phase was diluted with water (5 mL) and extracted with EtOAc (3 x 70 mL). The combined extracts were washed with brine (30 mL), dried over $MgSO_4$ and concentrated *in vacuo*. The crude iodofuran **25** was used in the next step without further purification.

The commercial palladium complex XPhos-Pd-G2 (**28**) (47 mg, 0.06 mmol, 0.25 eq.) was added to a stirred solution of **25** (200 mg, 0.24 mmol) and boronic ester **33** (76 mg, 0.26 mmol) in a mixture of THF (1.8 mL) and aqueous K_3PO_4 (3 M, 0.2 mL) at room temperature. The resulting mixture was stirred for 4 h at 50 °C (oil bath) before it was diluted with Et_2O (20 mL) and water (5 mL). The aqueous phase was extracted with Et_2O (3 x 70 mL), the combined organic layers were washed with brine (10 mL), dried over $MgSO_4$ and concentrated, and the residue was purified by flash chromatography on silica (hexanes:EtOAc, 3:1 to 2:1) to afford the title compound as a brown oil (97 mg, 39% yield over 2 steps). $[\alpha]_D^{20}$ = –48.7° (*c* = 1.0, $CHCl_3$); ^1H NMR (400 MHz, CD_2Cl_2): δ = 7.71 – 7.65 (m, 4H), 7.48 – 7.36 (m, 6H), 6.46 (s, 1H), 6.15 (d, *J* = 1.4 Hz, 1H), 5.27 (ddd, *J* = 7.8, 5.6, 0.9 Hz, 1H), 4.92 (d, *J* = 6.8 Hz, 1H), 4.54 (d, *J* = 6.8 Hz, 1H), 4.48 (ddq, *J* = 8.1, 6.2, 2.1 Hz, 1H), 4.31 (ddd, *J* = 7.5, 6.3, 0.9 Hz, 1H), 3.95 – 3.82 (m, 2H), 3.76 (s, 3H), 3.59 – 3.46 (m, 2H), 3.30 (s, 3H), 3.05 (tt, *J* = 9.9, 5.9 Hz, 1H), 2.95 (dtd, *J* = 10.5, 6.3,

5.2 Hz, 1H), 2.62 – 2.49 (m, 2H), 2.09 (d, J = 1.2 Hz, 3H), 2.01 – 1.90 (m, 1H), 1.83 (d, J = 2.1 Hz, 3H), 1.82 – 1.71 (m, 4H), 1.07 (s, 9H); ^{13}C NMR (101 MHz, CD_2Cl_2): δ = 170.1, 164.4, 157.6, 152.3, 135.99, 135.97, 135.7, 133.80, 133.75, 130.1, 128.1, 116.4, 116.1, 108.9, 94.1, 82.4, 77.7, 71.5, 64.8, 62.3, 61.5, 55.7, 51.7, 47.5, 44.6, 41.7, 34.2, 33.5, 27.0, 20.7, 19.4, 19.2, 3.6; IR (film): $\tilde{\nu}$ = 3503, 3071, 3047, 2930, 2858, 1741, 1718, 1597, 1549, 1442, 1428, 1377, 1233, 1149, 1110, 1058, 1031, 975, 939, 919, 823, 778, 742, 704, 611, 505, 491 cm^{-1}. HRMS (ESI): m/z calcd. for $C_{41}H_{52}O_9SiNa$ [M+Na$^+$]: 739.3272, found: 739.3275.

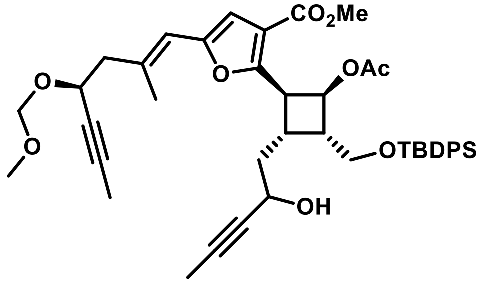

Methyl 2-((1*R*,2*S*,3*S*,4*S*)-2-acetoxy-3-(((*tert*-butyldiphenylsilyl)oxy)methyl)-4-(2-hydroxypent-3-yn-1-yl)cyclobutyl)-5-((*S*,*E*)-4-(methoxymethoxy)-2-methyl-hept-1-en-5-yn-1-yl)furan-3-carboxylate (35). Sodium bicarbonate (35 mg, 0.42 mmol) and Dess-Martin-periodinane (36 mg, 0.08 mmol) were added to a solution of alcohol **34** (20 mg, 0.03 mmol) in CH_2Cl_2 (0.8 mL) at 0 °C. The resulting mixture was stirred at this temperature for 3.5 h before it was diluted with CH_2Cl_2 (3 mL). The reaction was quenched with a mixture of sat. aq. $Na_2S_2O_3$/$NaHCO_3$ (1:1 *v/v*, 3 mL) and the resulting mixture was vigorously stirred for 30 min. The aqueous phase was diluted with water (2 mL) and extracted with CH_2Cl_2 (3 x 30 mL). The combined organic layers were washed with sat. aq. $NaHCO_3$ (3 x 20 mL), dried over $MgSO_4$ and concentrated. The crude aldehyde thus formed was used in the next step without further purification.

1-Propinylmagnesium chloride (0.5 M in THF, 0.13 mL, 0.07 mmol) was added dropwise to a stirred solution of this crude aldehyde (19 mg, 0.03 mmol) in THF (1.3 mL) at –78 °C. The resulting mixture was stirred for 10 min at –78 °C before the reaction was quenched with sat. aq. NH_4Cl (1 mL). After reaching room temperature, the mixture was diluted with *tert*-butyl methyl ether (10 mL) and water (3 mL). The aqueous phase was extracted with *tert*-butyl methyl ether (3 x 20 mL), the combined organic layers were washed with brine (4 mL), dried over $MgSO_4$ and concentrated. The residue was purified by flash chromatography on silica (hexanes:EtOAc, 3:1) to provide the title compound as a faint yellow oil (7.8 mg, 39% yield over 2 steps). $[\alpha]_D^{20}$ = –68.0° (c = 0.25, $CHCl_3$); ^{1}H NMR (400 MHz, CD_2Cl_2, mixture of diastereomers): δ = 7.69 (dt, J = 7.9, 1.5 Hz, 4H), 7.48 – 7.37 (m, 6H), 6.46 (d, J = 2.1 Hz, 1H), 6.16 (dt, J = 1.4, 0.7 Hz, 1H), 5.30 – 5.23 (m, 1H), 4.92 (d, J = 6.8 Hz, 1H), 4.54 (d, J = 6.8 Hz, 1H), 4.48 (tt, J = 6.0, 2.1 Hz, 1H), 4.40 – 4.33 (m, 1H), 4.28 (s, 1H), 3.95 – 3.81 (m, 2H), 3.75 (s, 3H), 3.30 (d, J = 0.7 Hz, 3H), 3.28 – 3.18 (m, 1H), 2.96 – 2.85 (m, 1H), 2.63 – 2.49 (m, 2H), 2.10 (d, J = 1.4 Hz, 3H), 2.08 – 2.01 (m, 1H), 2.01 – 1.89 (m, 1H), 1.83 (d, J = 2.0 Hz, 3H), 1.79 (d, J = 1.4 Hz, 3H), 1.74 (d, J = 2.1 Hz, 1H, minor isomer), 1.72 (d, J = 2.1 Hz, 2H, major isomer), 1.08 (d, J = 0.7 Hz, 9H); ^{13}C NMR (101 MHz, CD_2Cl_2, mixture of diastereomers): δ = 170.1, 164.3, 157.44, 157.39, 152.29, 152.25, 136.03, 136.01,

135.7, 135.6, 133.73, 133.68, 133.64, 130.1, 128.1, 116.44, 116.39, 116.0, 108.93, 108.90, 94.1, 82.4, 81.4, 81.2, 80.6, 80.3, 77.7, 71.8, 71.7, 64.78, 64.76, 62.3, 61.7, 61.5, 55.8, 51.6, 47.5, 44.6, 41.9, 41.7, 38.8, 33.8, 27.0, 20.7, 19.4, 19.2, 3.6, 3.5; IR (film): $\tilde{\nu}$ = 3466, 3071, 2952, 2930, 2857, 1740, 1717, 1597, 1548, 1471, 1441, 1428, 1376, 1232, 1148, 1106, 1058, 1029, 938, 919, 823, 779, 742, 704, 612, 505, 490 cm^{-1}. HRMS (ESI): *m/z* calcd. for $C_{44}H_{54}O_9SiNa$ [$M+Na^+$]: 777.3429, found: 777.3420.

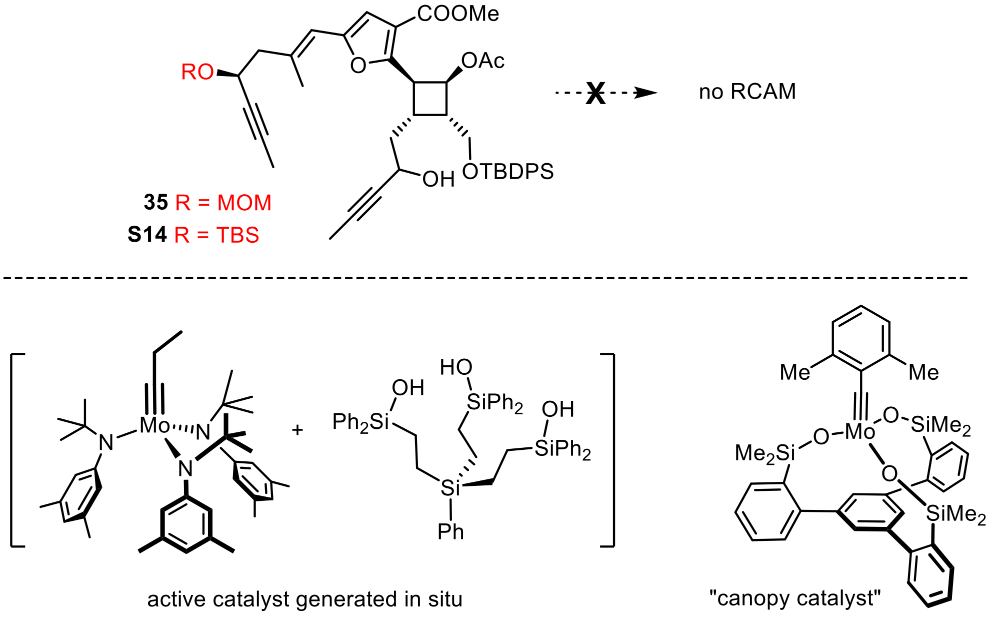

Scheme S1. Attempted RCAM reactions. In addition to the failed attempts of converting compound **35** into a monomeric cycloalkyne with the aid of an active catalyst generated in situ[4] or with a structurally well-defined "canopy catalyst" (see main text),[5] preliminary experiments using the analogous TBS-ether derivative **S14** were also met with failure. The protecting group pattern reflects previous experiences which had shown that recourse to one protected and one unprotected –OH group is a favorable choice when cycloalkynes comprising a butyne-1,4-diol subunit need to be made.[4]

1H (CDCl3, 400.12 MHz)

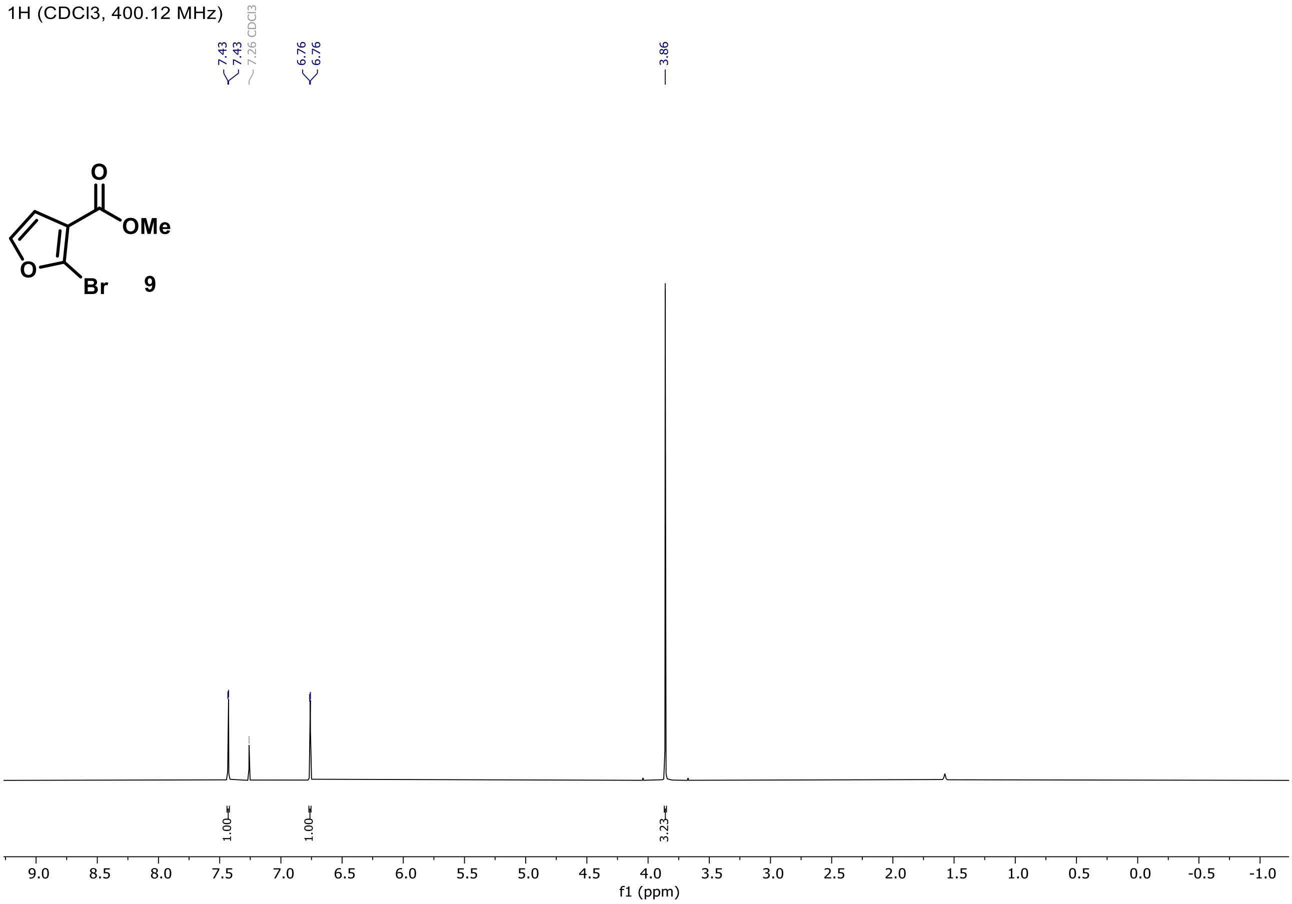

13C (CDCl3, 100.62 MHz)

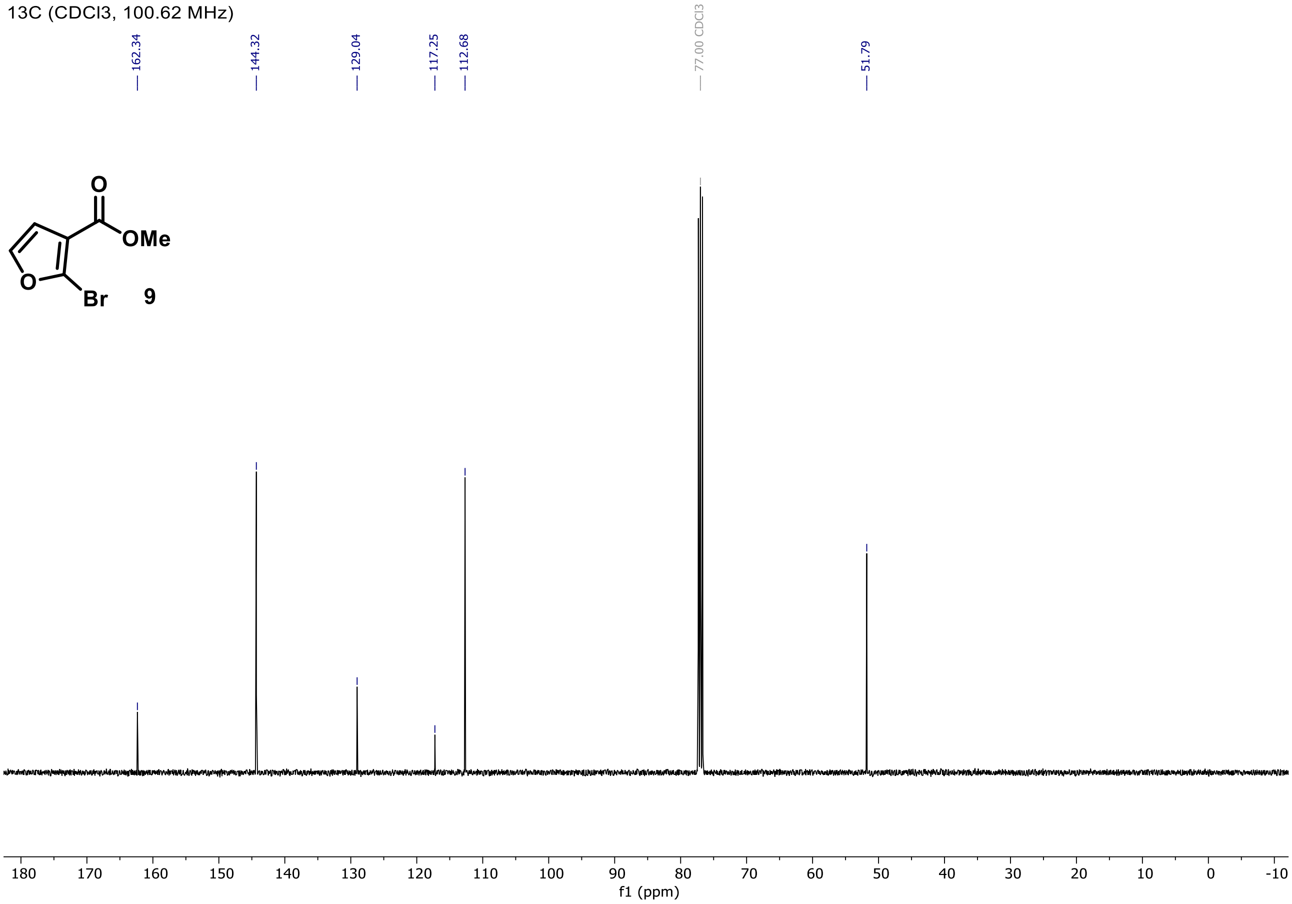

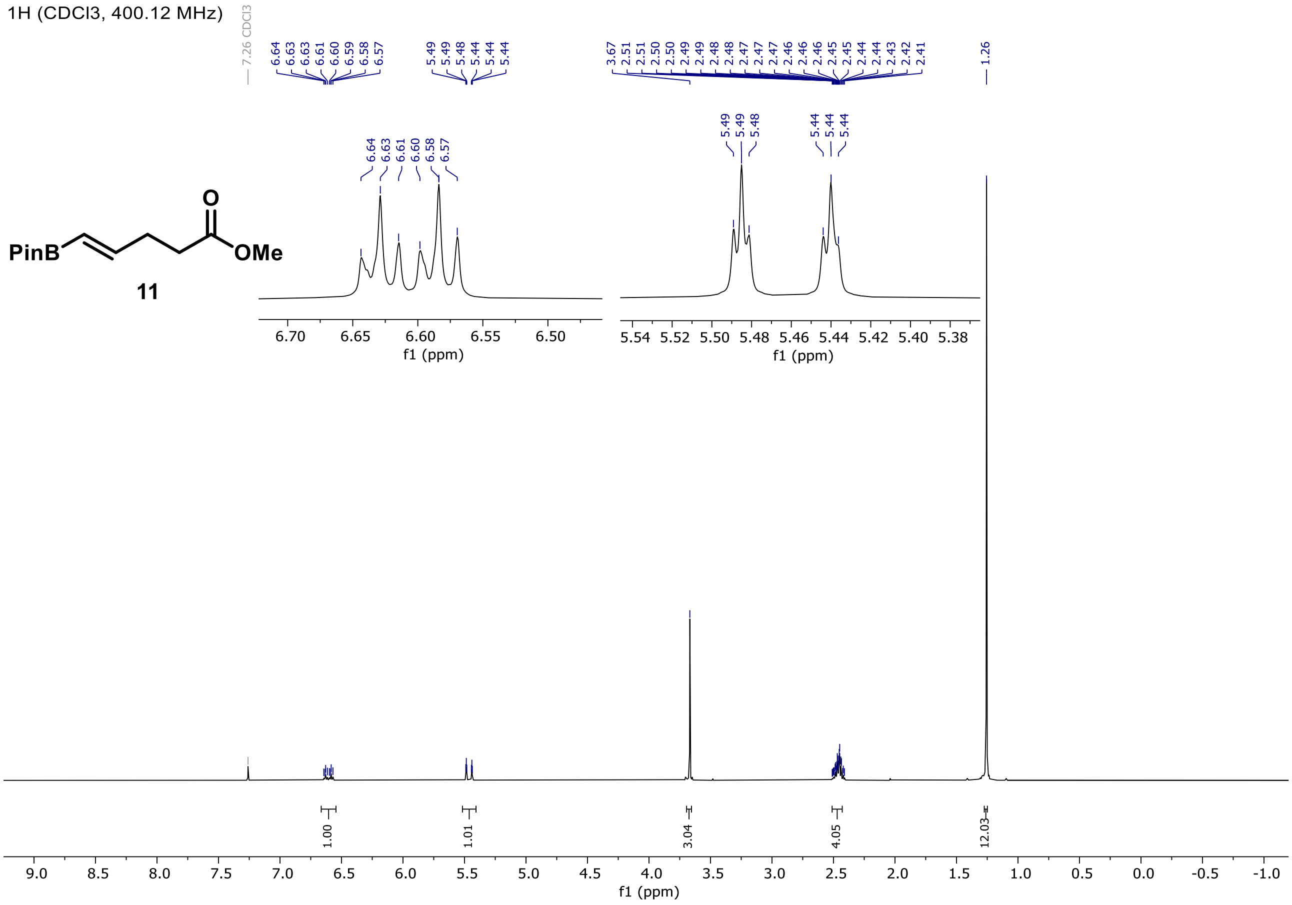
1H (CDCl3, 400.12 MHz)
PinB
OMe
O
11
f1 (ppm)

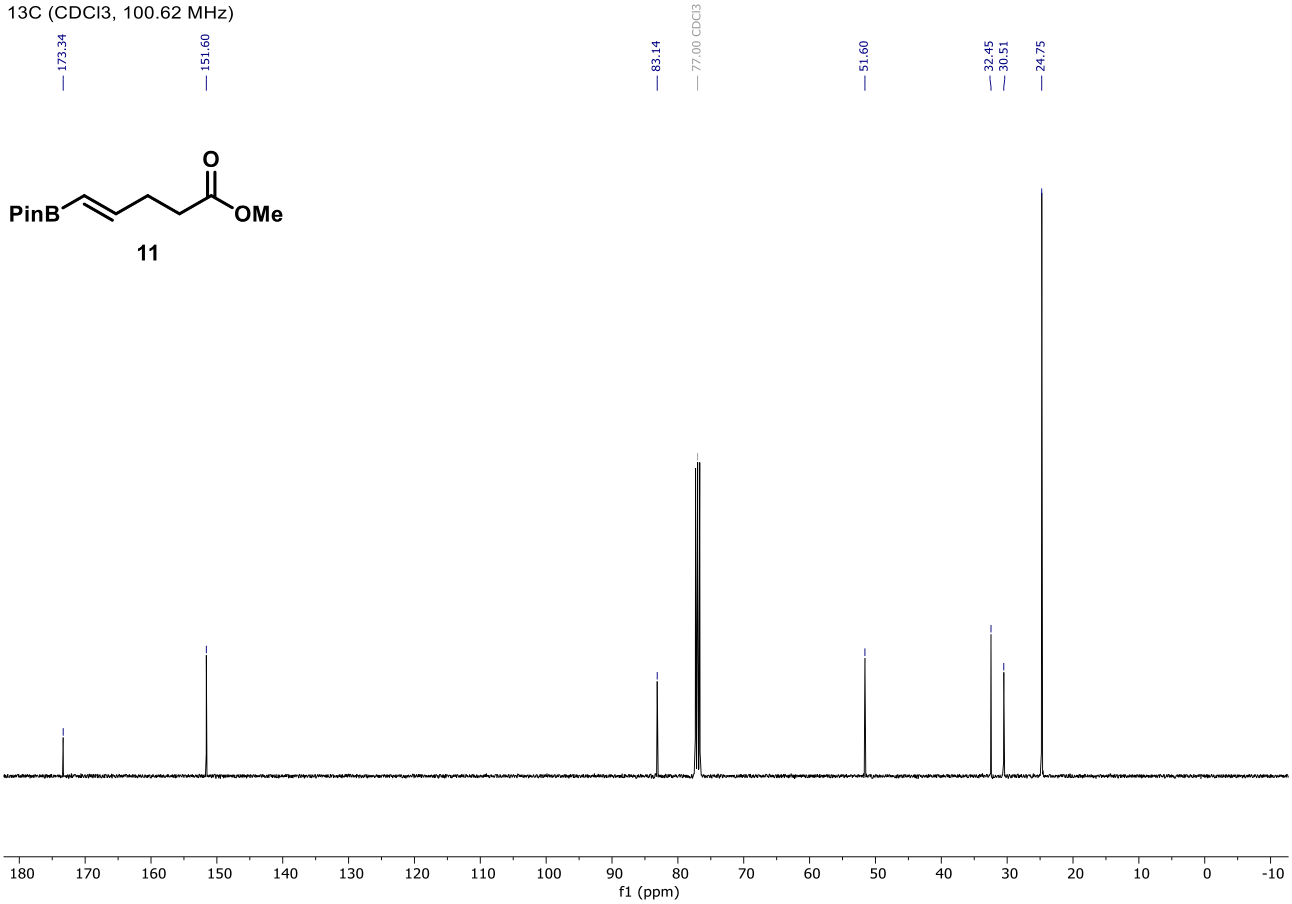
13C (CDCl3, 100.62 MHz)
PinB
OMe
O
11
f1 (ppm)

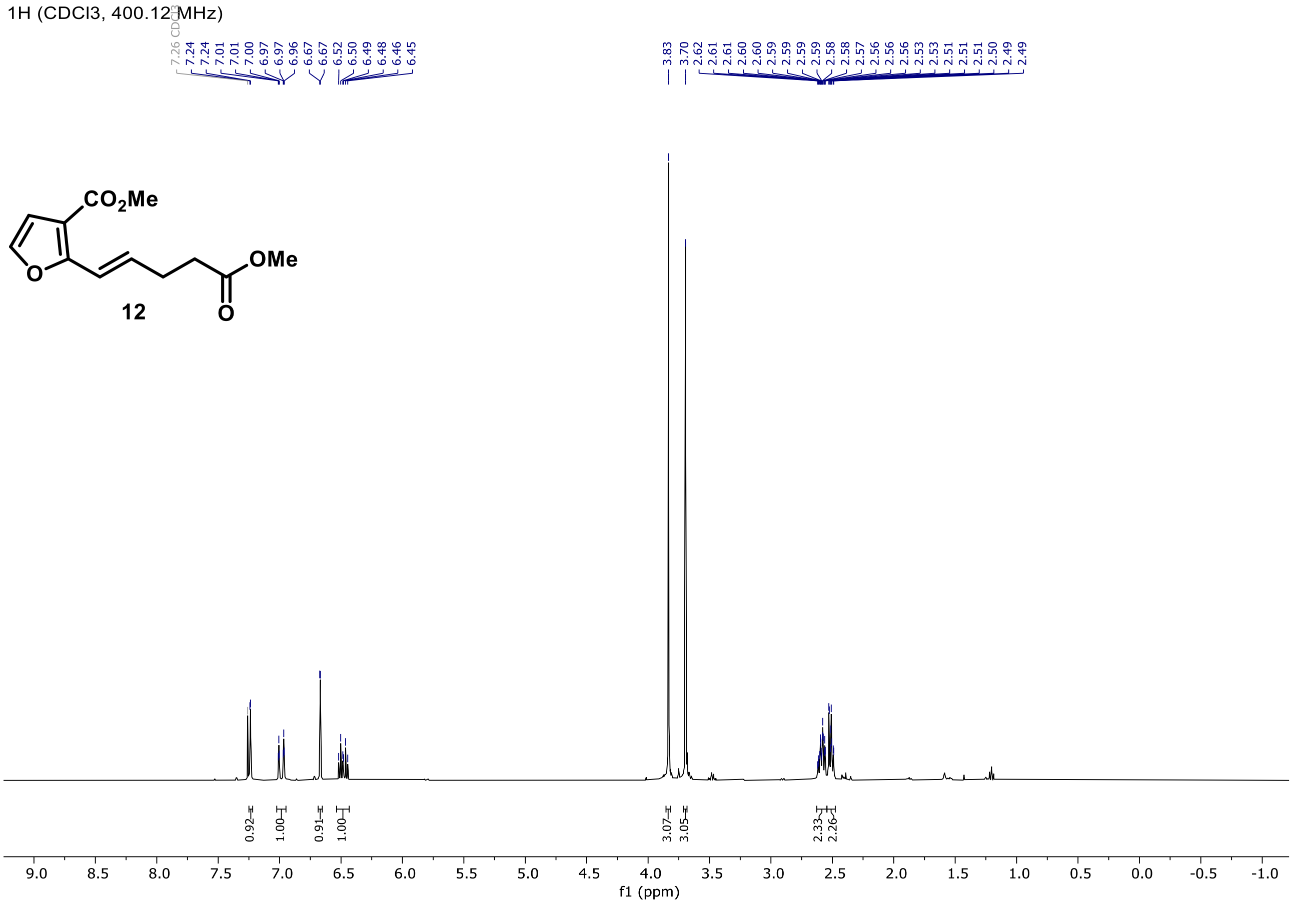
1H (CDCl3, 400.12 MHz)
7.26 CDCl3
7.24 7.24 7.01 7.00 6.97 6.97 6.96 6.67 6.67 6.52 6.50 6.49 6.48 6.46 6.45
3.83 3.70 2.62 2.61 2.61 2.60 2.60 2.59 2.59 2.59 2.59 2.58 2.58 2.57 2.56 2.56 2.56 2.53 2.53 2.51 2.51 2.51 2.50 2.49 2.49
CO2Me
OMe
O
12
0.92 1.00 0.91 1.00 3.07 3.05 2.33 2.26
9.0 8.5 8.0 7.5 7.0 6.5 6.0 5.5 5.0 4.5 4.0 3.5 3.0 2.5 2.0 1.5 1.0 0.5 0.0 -0.5 -1.0
f1 (ppm)

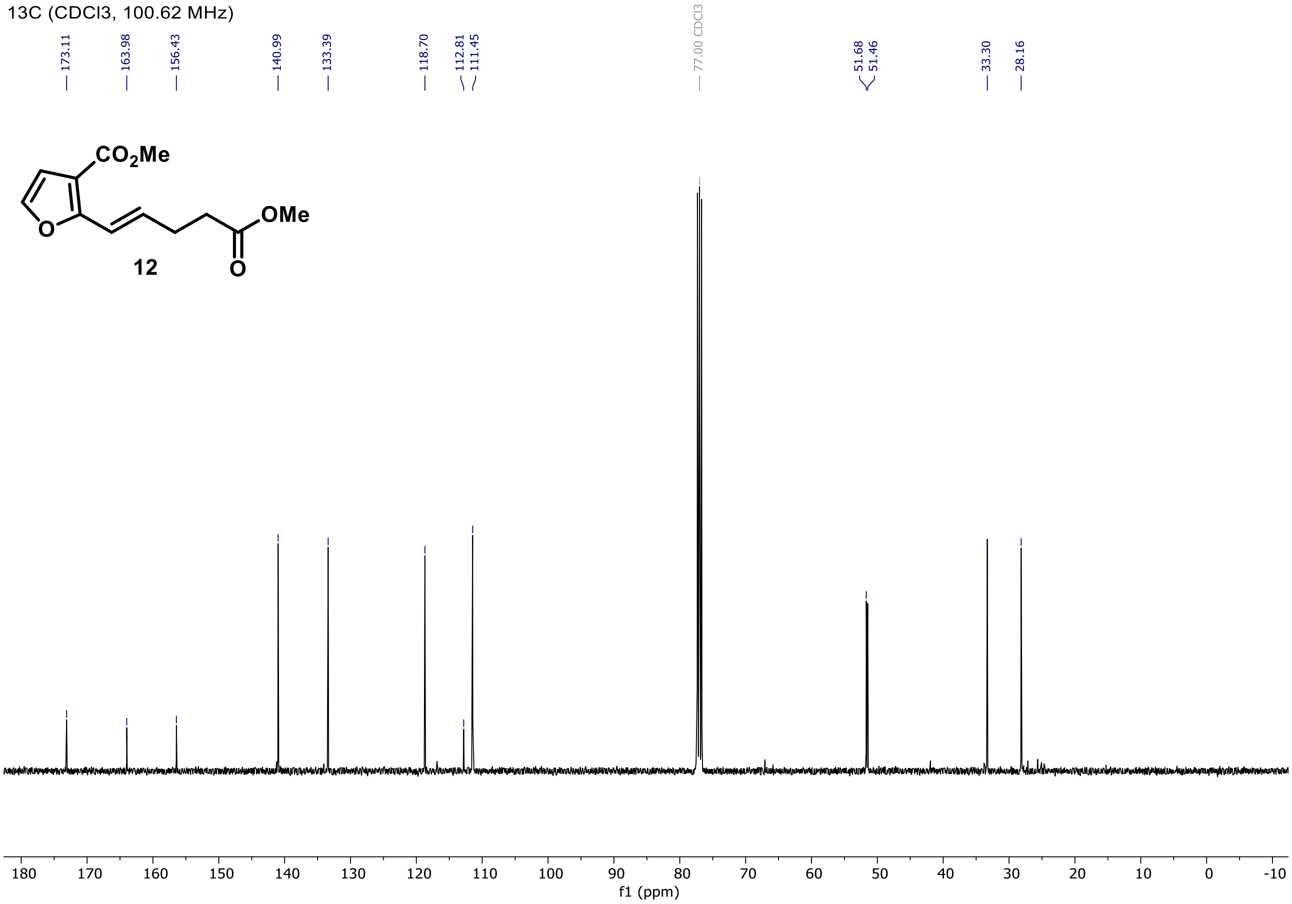
13C (CDCl3, 100.62 MHz)
173.11
163.98
156.43
140.99
133.39
118.70
112.81
111.45
77.00 CDCl3
51.68
51.46
33.30
28.16
CO2Me
OMe
O
12
180 170 160 150 140 130 120 110 100 90 80 70 60 50 40 30 20 10 0 -10
f1 (ppm)

1H (CDCl3, 400.12 MHz)

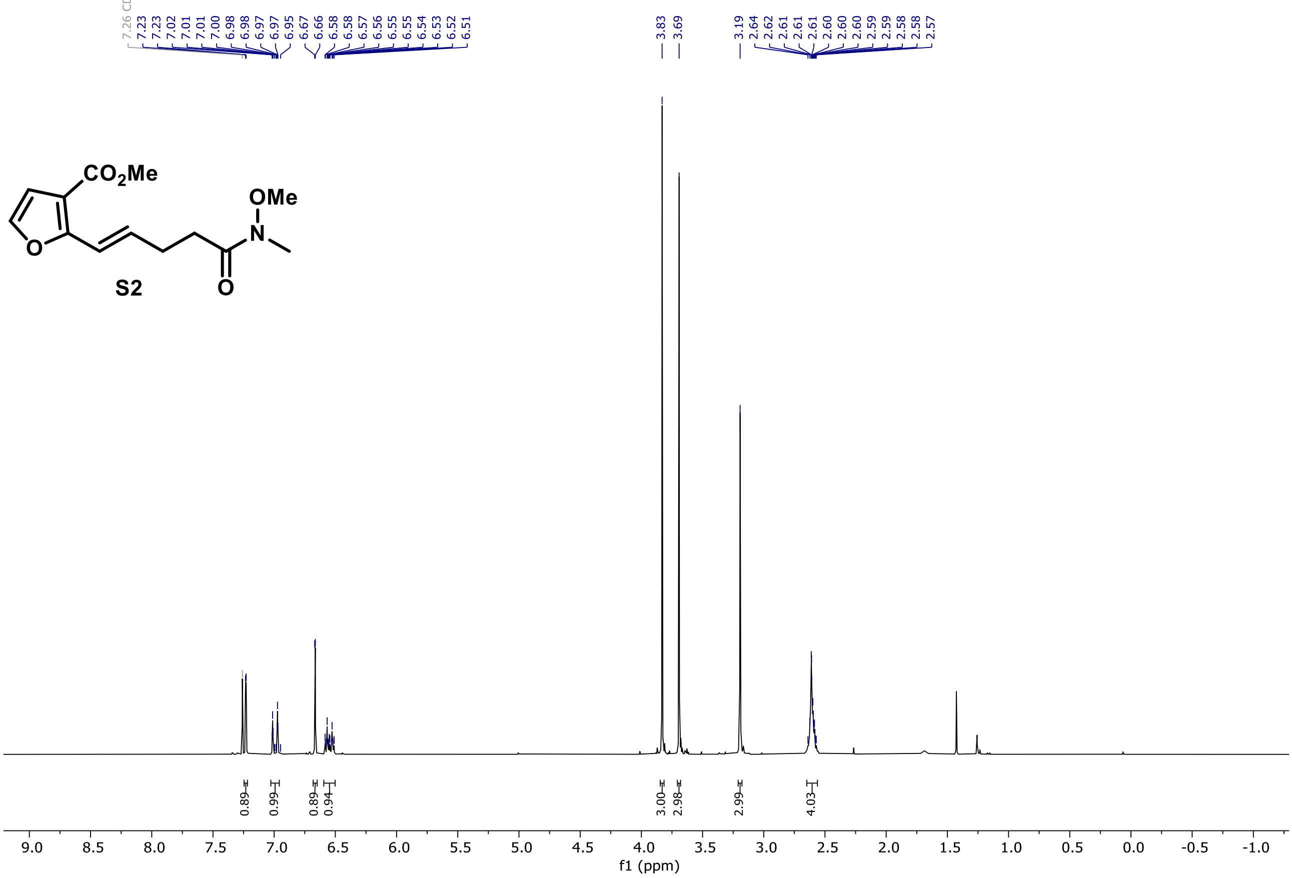

13C (CDCl3, 100.62 MHz)

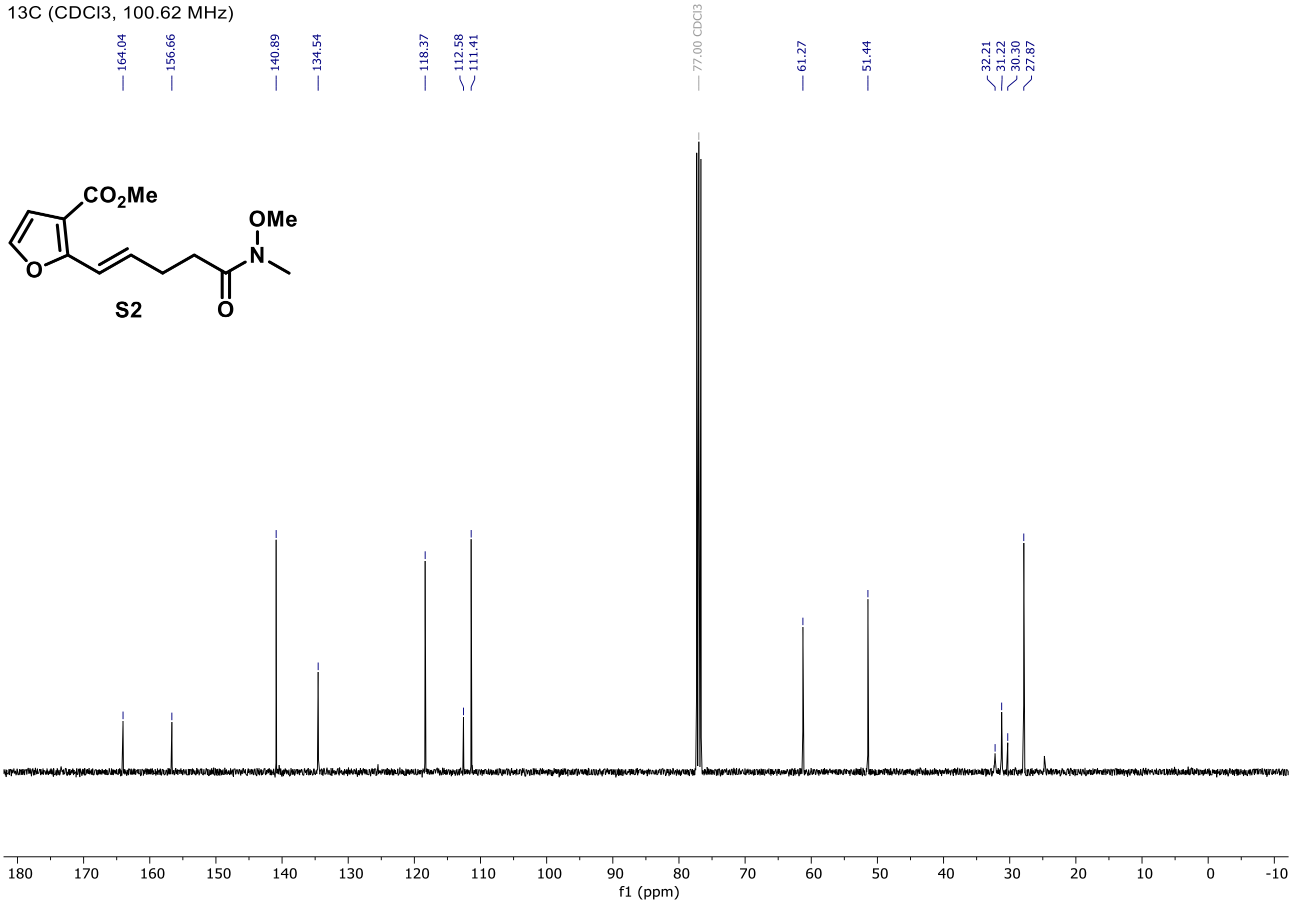

1H (CDCl3, 400.12 MHz)

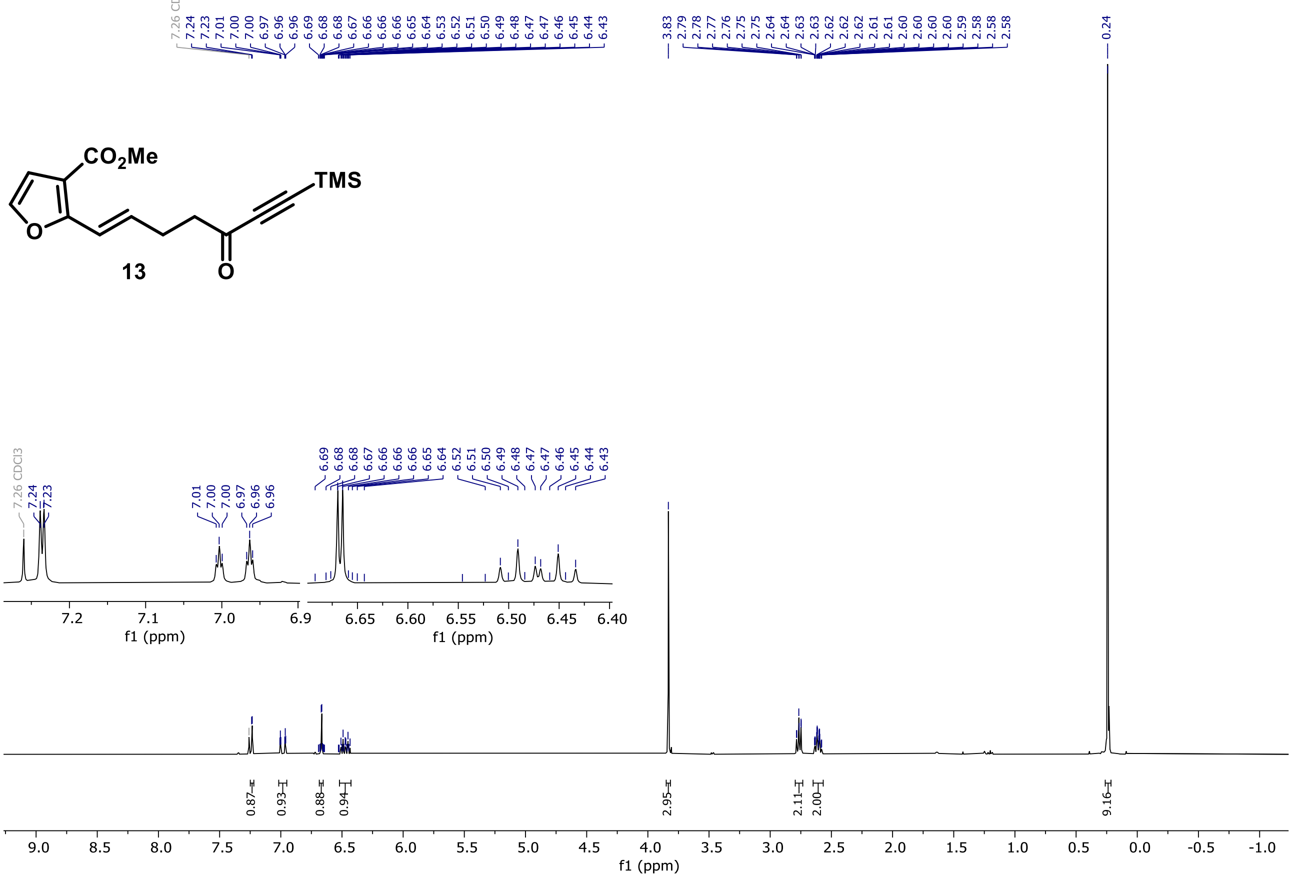

13C (CDCl3, 100.62 MHz)

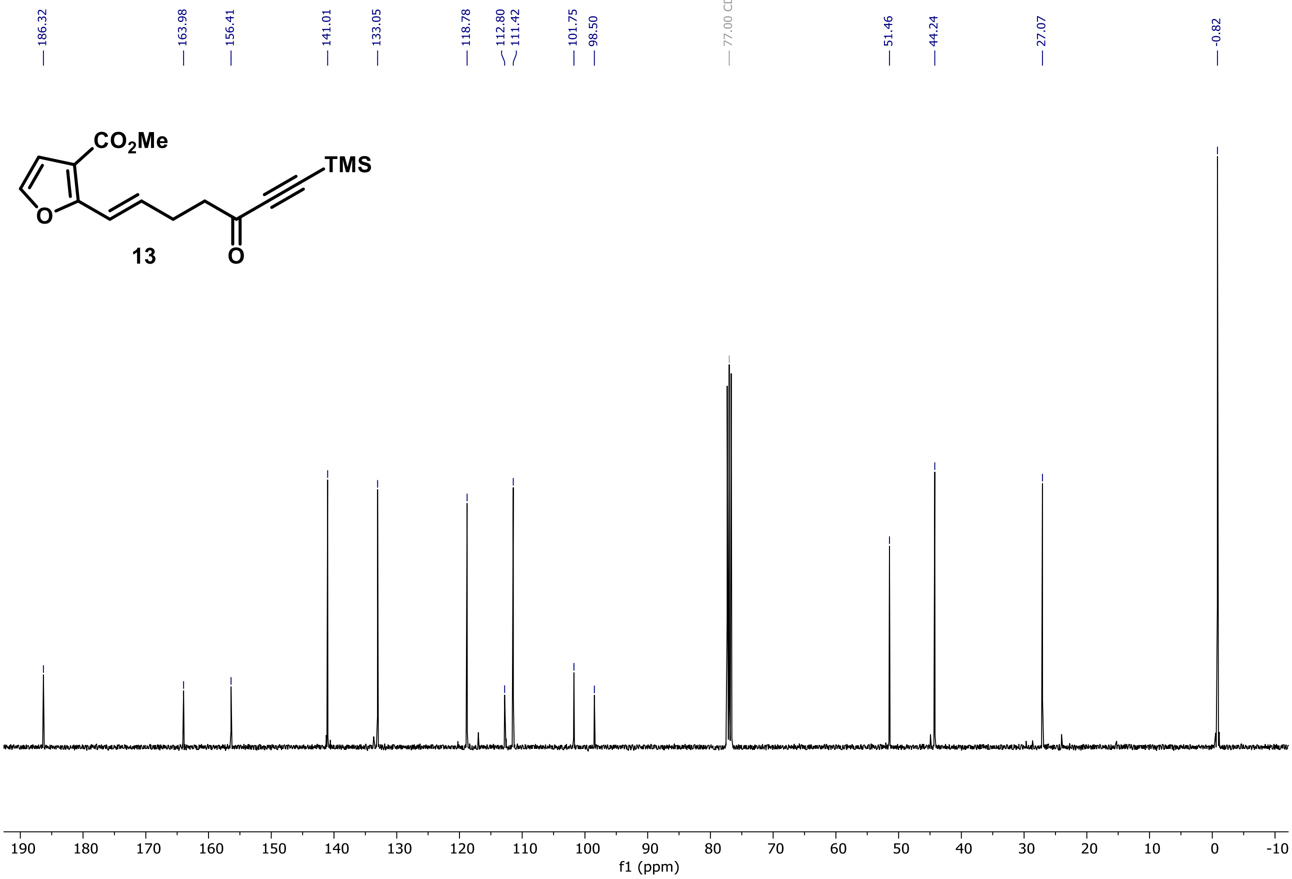

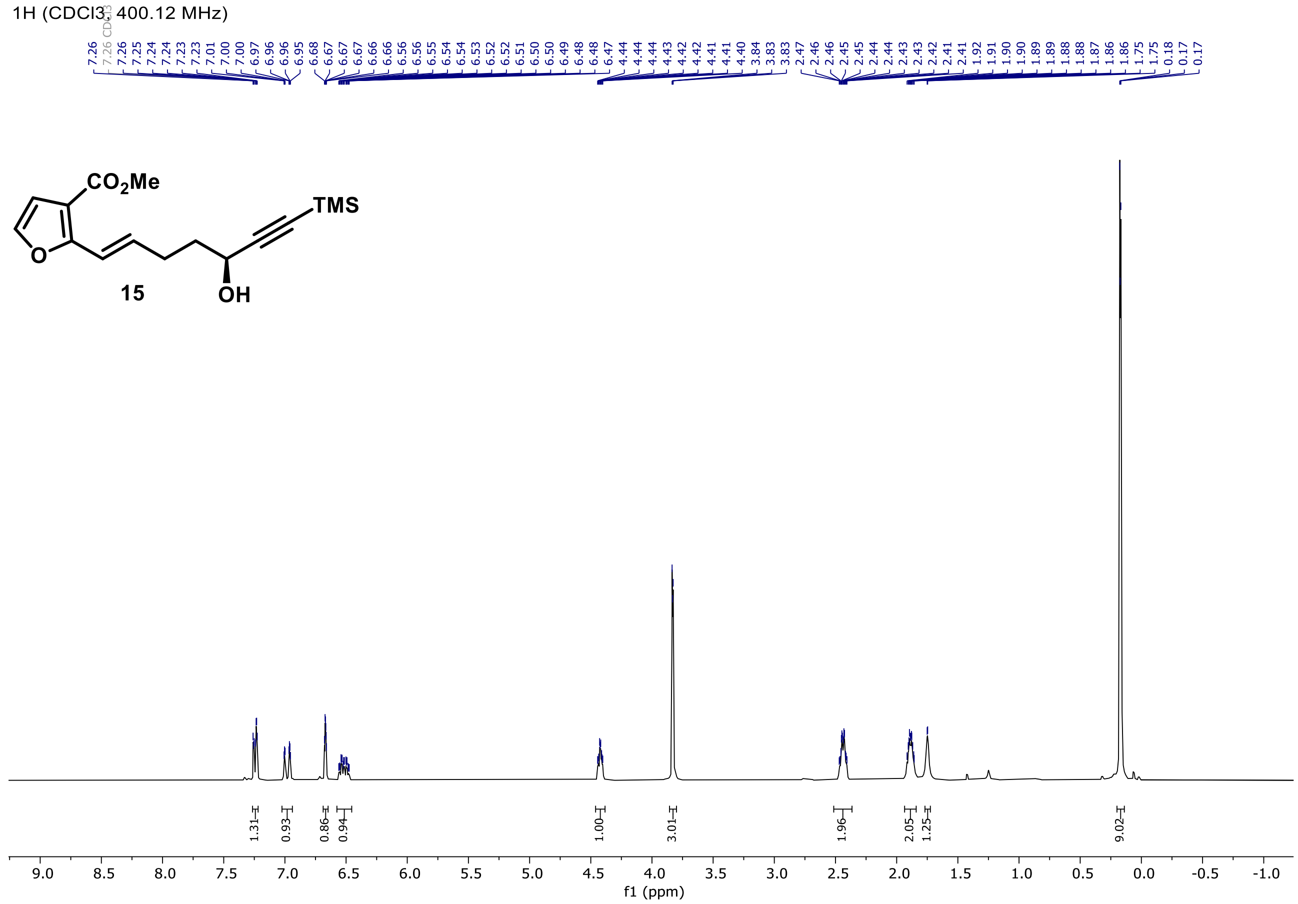
1H (CDCl3, 400.12 MHz)
7.26 7.26 CDCl3 7.26 7.25 7.24 7.24 7.23 7.23 7.01 7.00 7.00 6.97 6.96 6.96 6.95 6.68 6.67 6.67 6.67 6.66 6.66 6.56 6.56 6.55 6.54 6.54 6.53 6.53 6.52 6.52 6.51 6.50 6.50 6.49 6.48 6.48 6.47 4.44 4.44 4.44 4.43 4.42 4.42 4.41 4.41 4.40 3.84 3.83 2.47 2.46 2.45 2.45 2.44 2.44 2.43 2.43 2.42 2.41 2.41 1.92 1.91 1.90 1.89 1.88 1.88 1.87 1.86 1.86 1.75 1.75 0.18 0.17 0.17
CO2Me
TMS
O
15
OH
1.31 0.93 0.86 0.94 1.00 3.01 1.96 2.05 1.25 9.02
9.0 8.5 8.0 7.5 7.0 6.5 6.0 5.5 5.0 4.5 4.0 3.5 3.0 2.5 2.0 1.5 1.0 0.5 0.0 -0.5 -1.0
f1 (ppm)

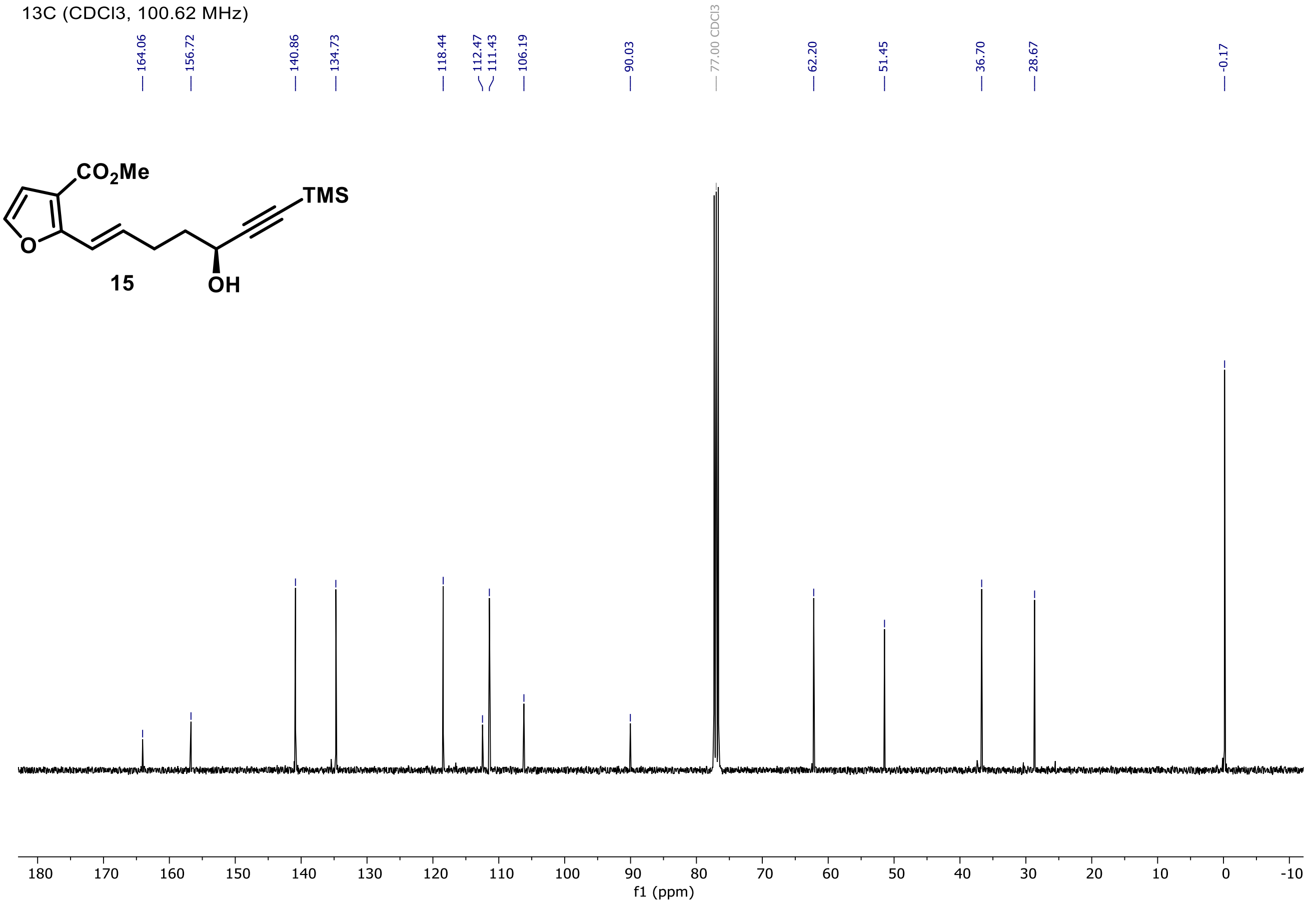
13C (CDCl3, 100.62 MHz)
164.06 156.72 140.86 134.73 118.44 112.47 111.43 106.19 90.03 77.00 CDCl3 62.20 51.45 36.70 28.67 -0.17
CO2Me
TMS
O
15
OH
180 170 160 150 140 130 120 110 100 90 80 70 60 50 40 30 20 10 0 -10
f1 (ppm)

1H (CDCl3, 400.12 MHz)

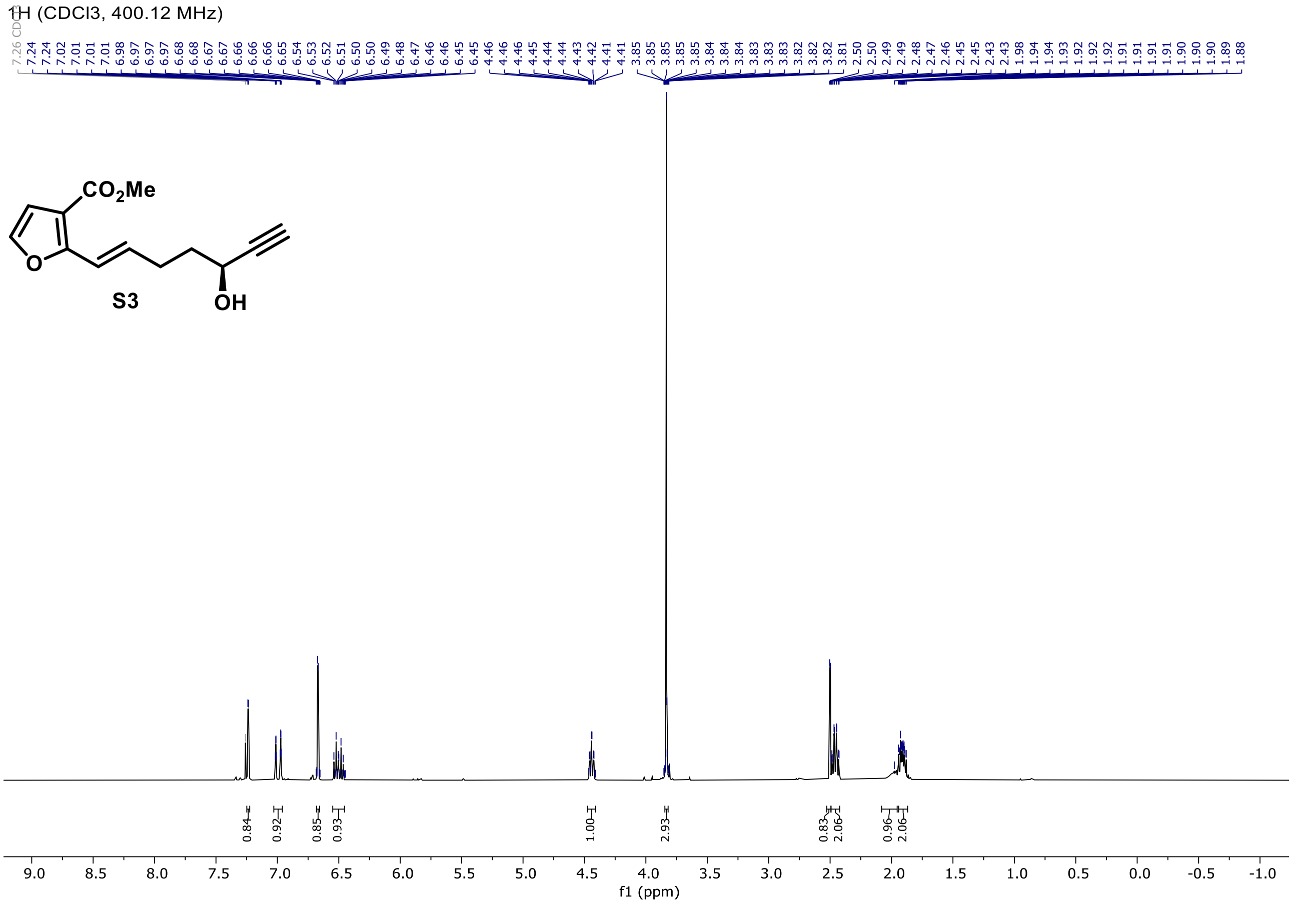

13C (CDCl3, 100.62 MHz)

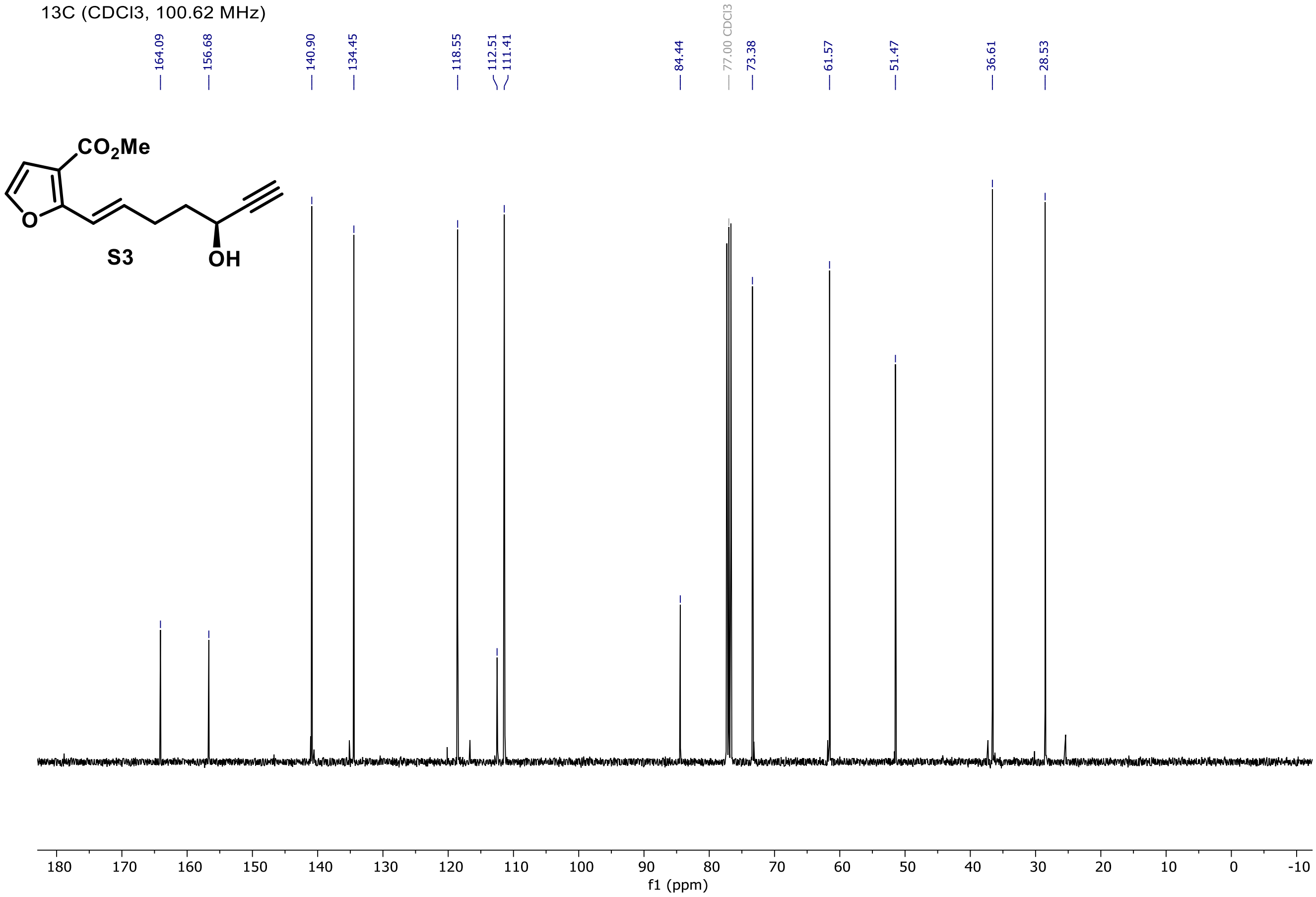

1H (CDCl3, 400.12 MHz)

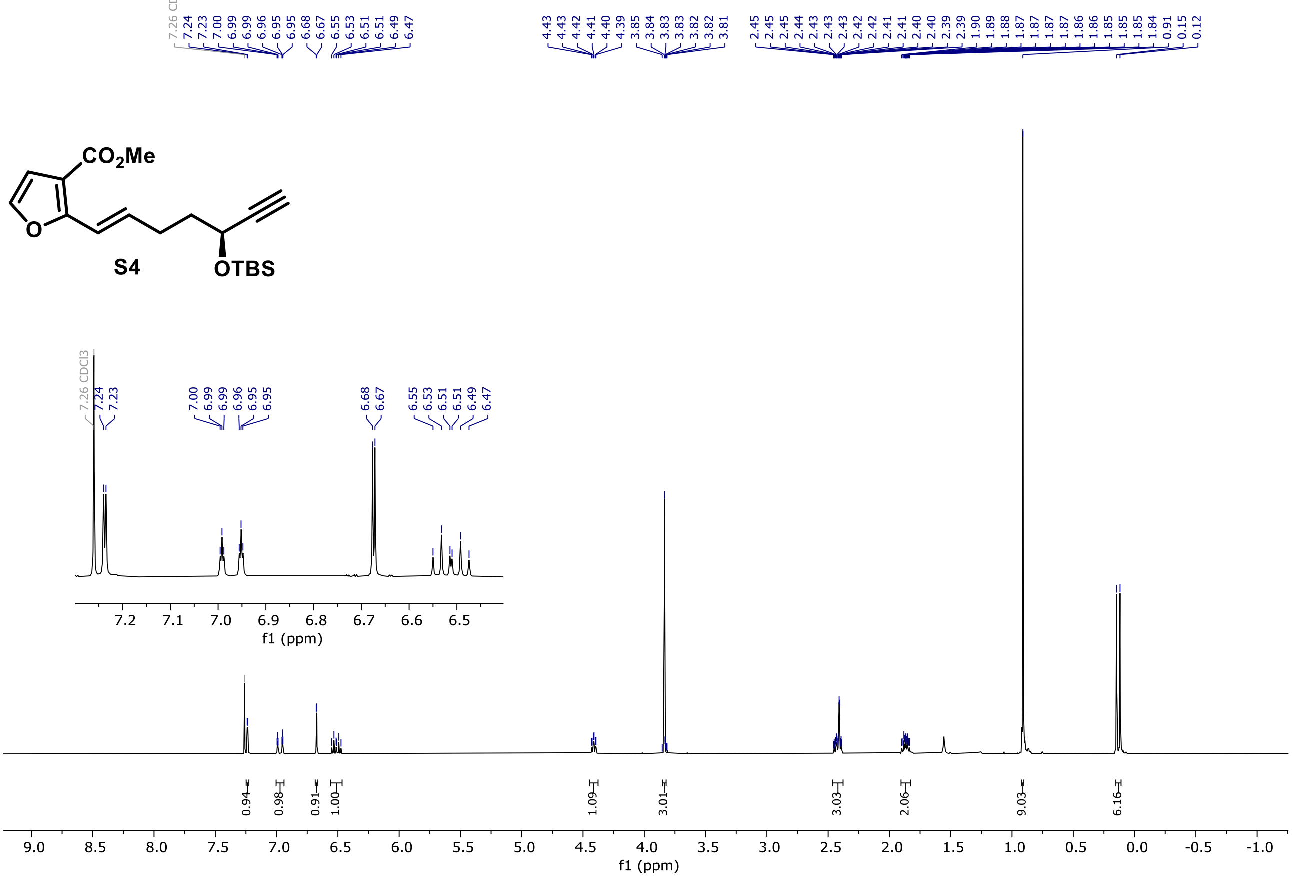

13C (CDCl3, 100.62 MHz)

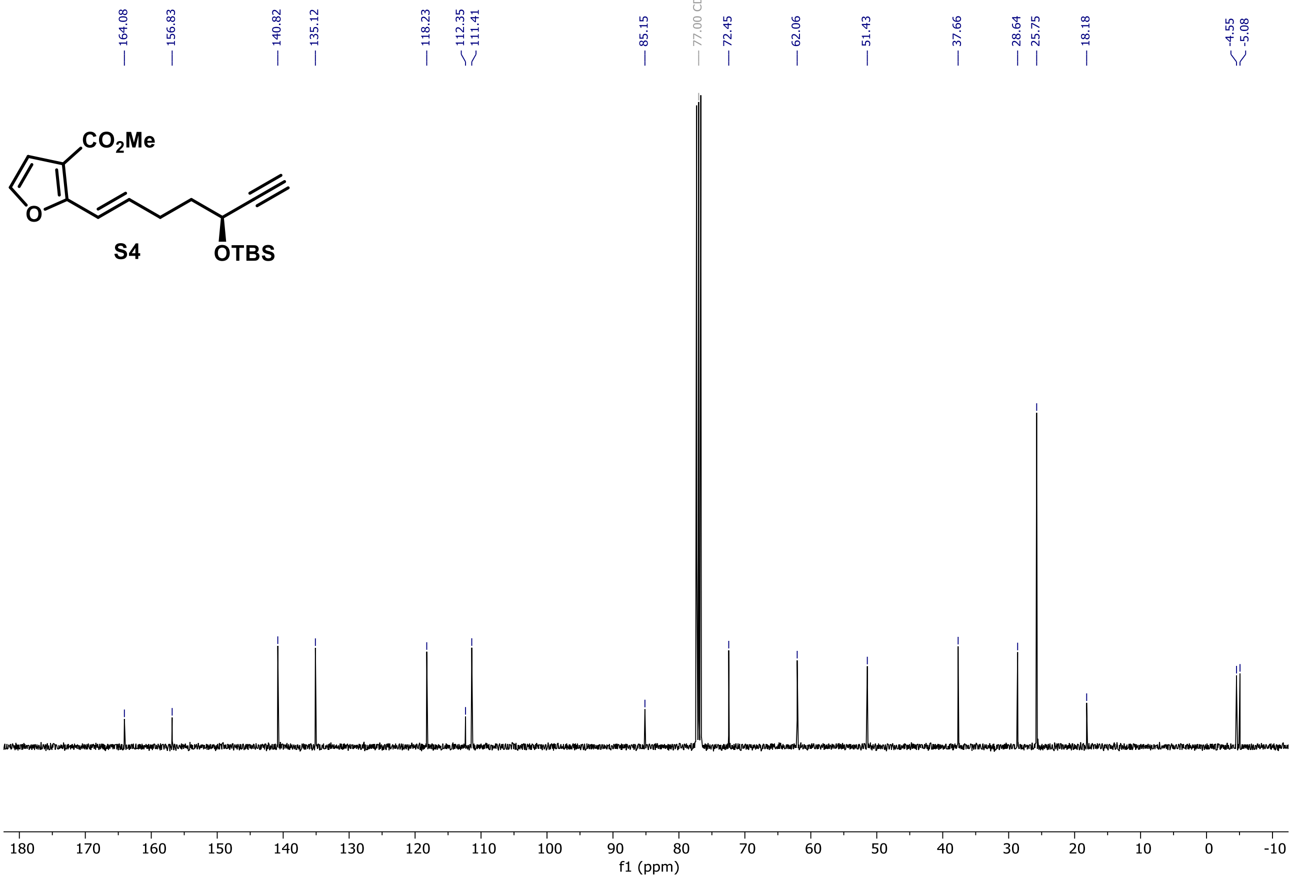

1H (CDCl3, 400.12 MHz)

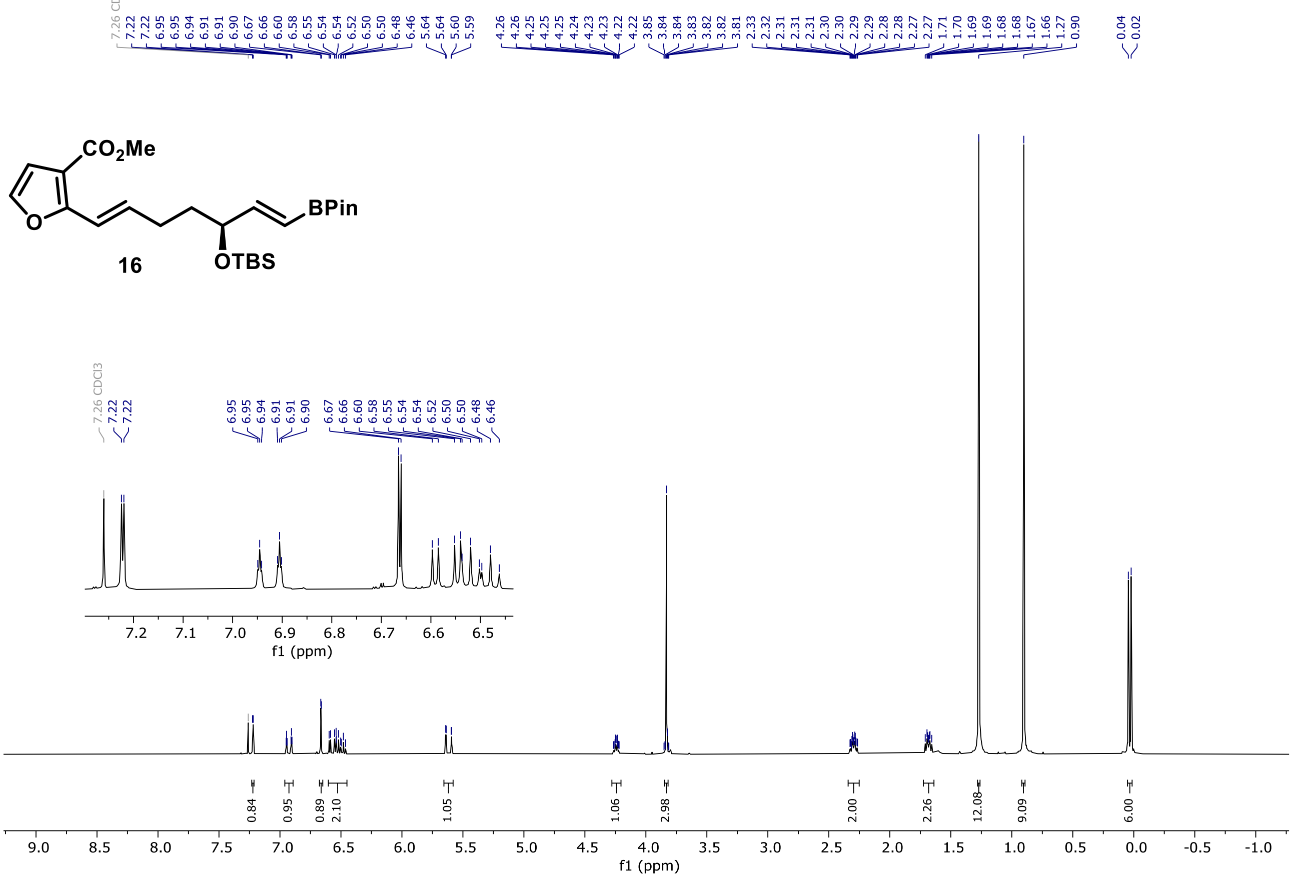

13C (CDCl3, 100.62 MHz)

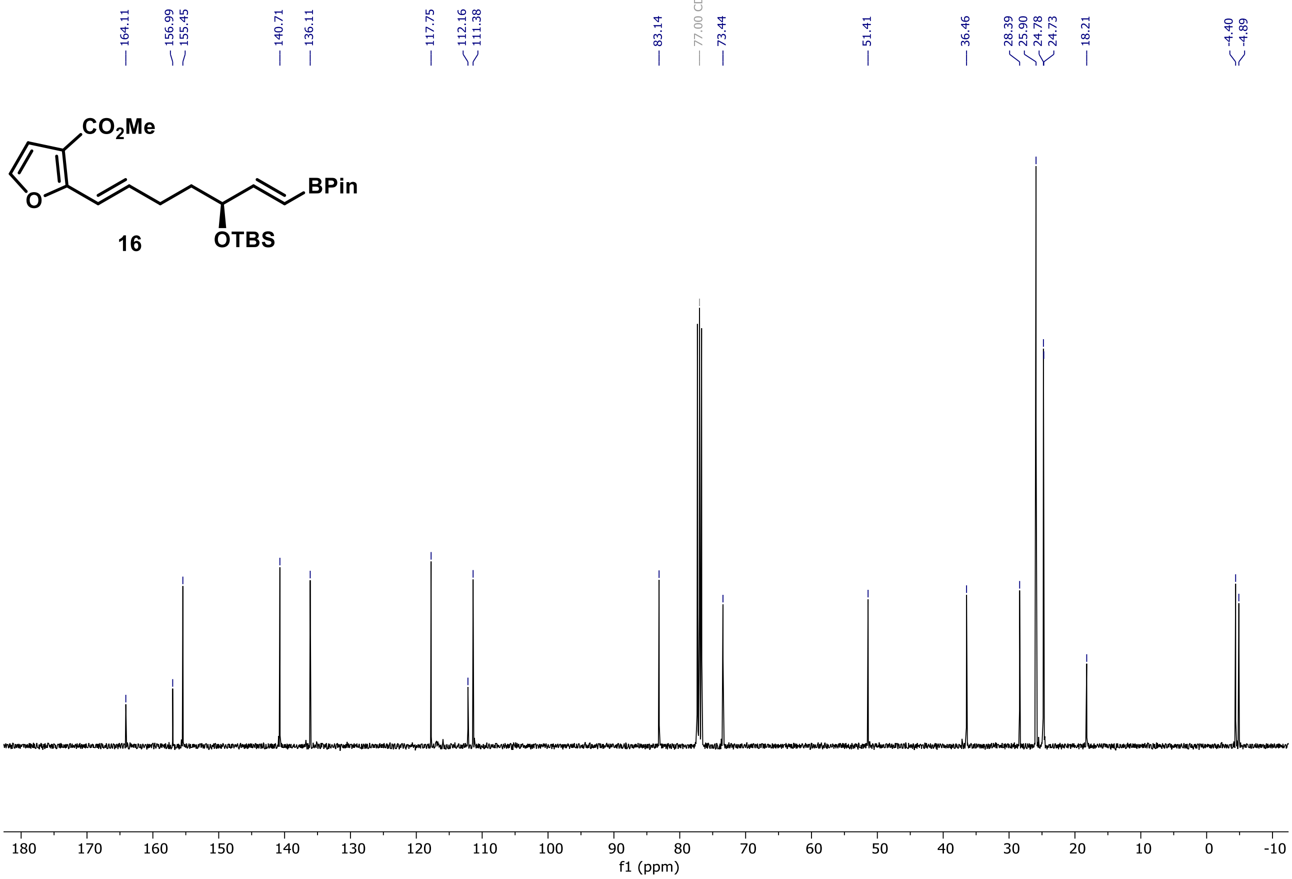

1H (CDCl3, 600.20 MHz)

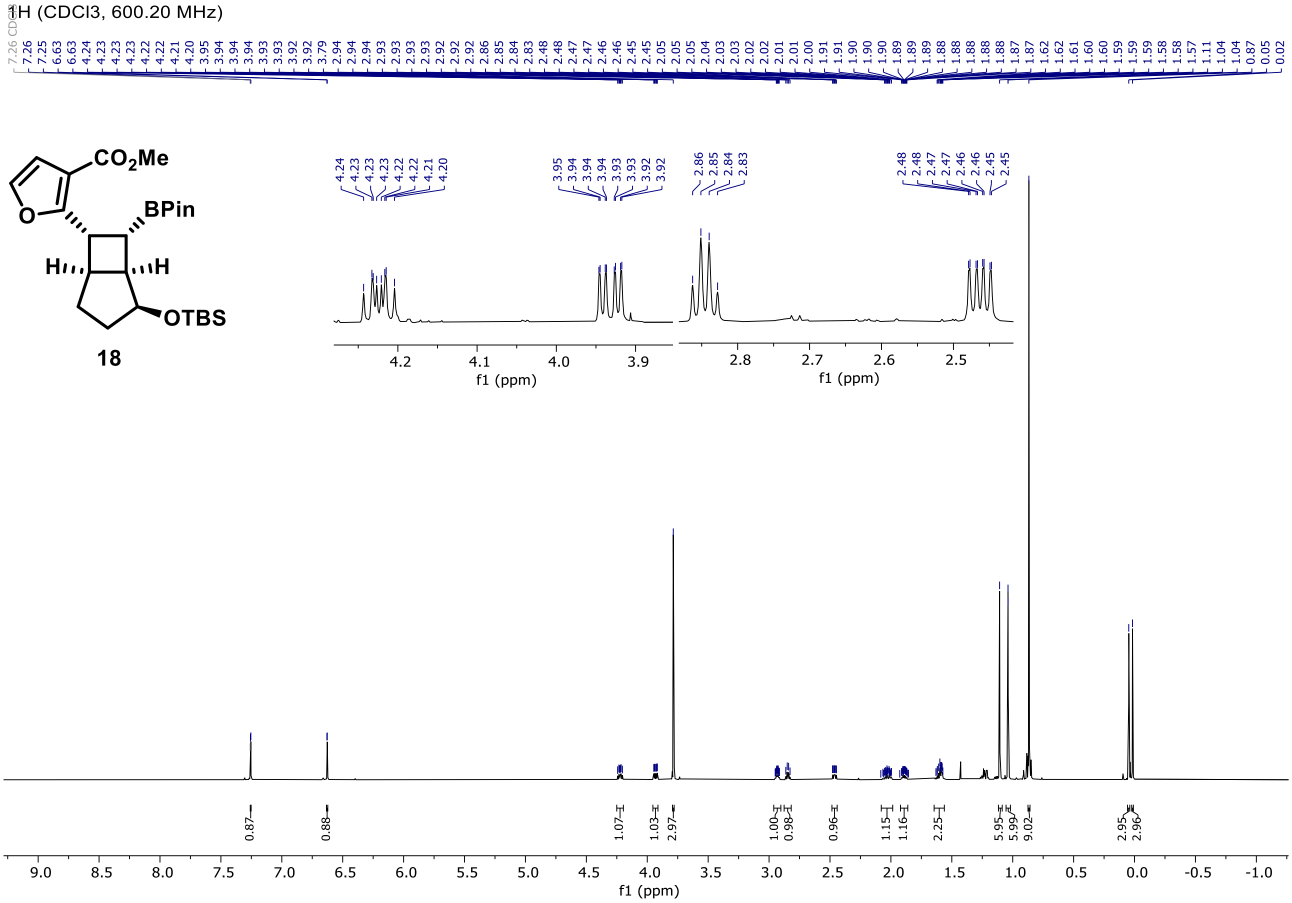

13C (CDCl3, 150.94 MHz)

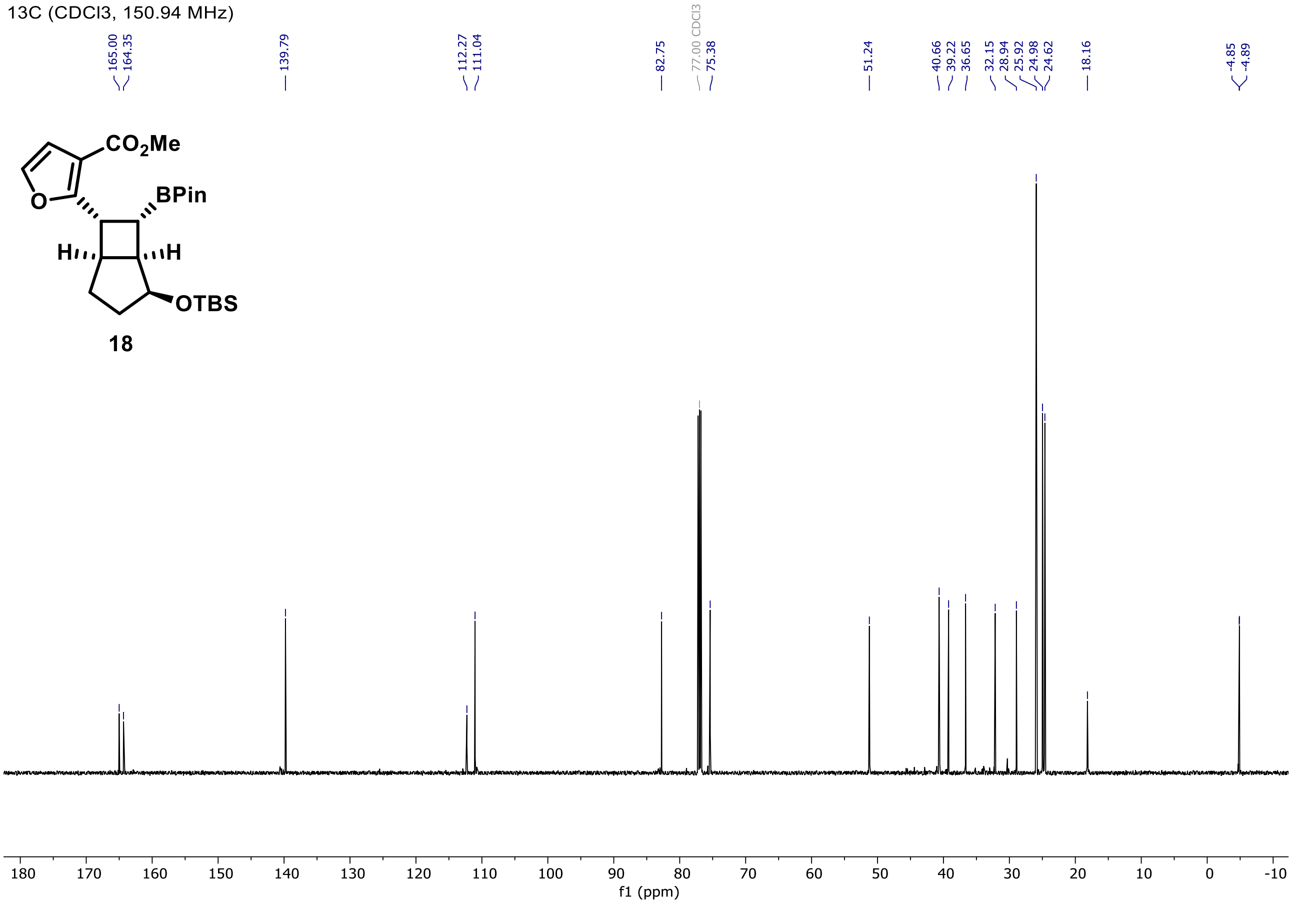

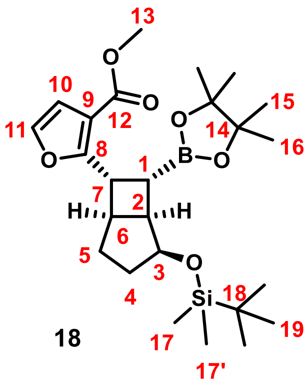

18

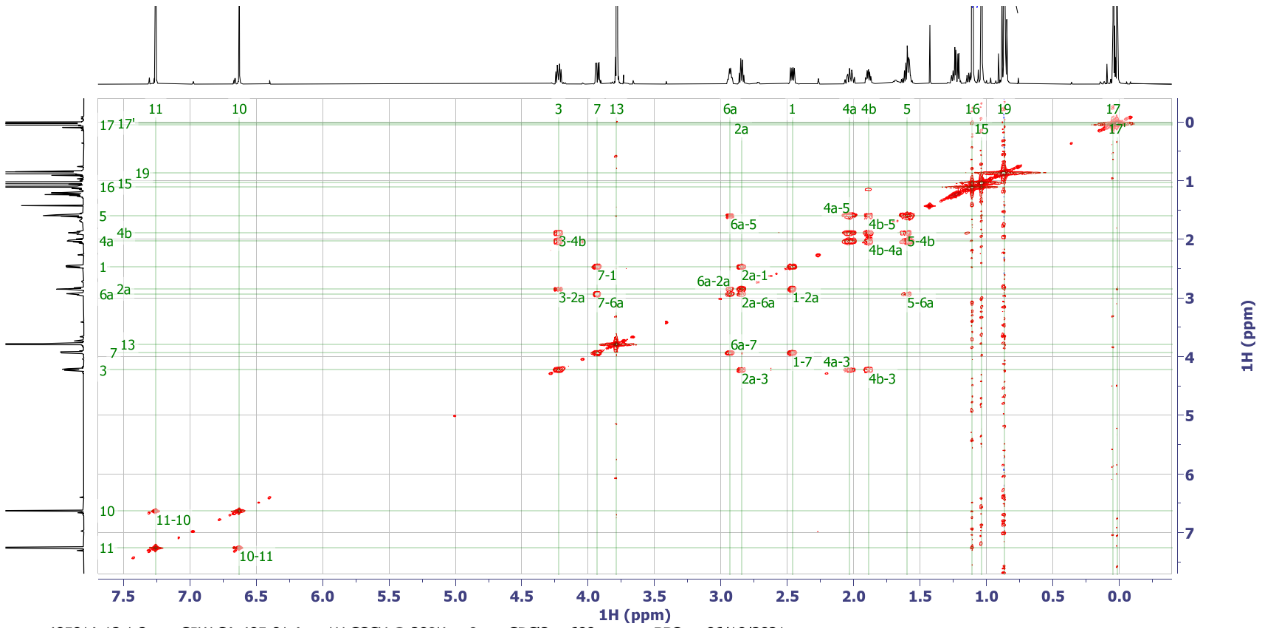

spwsa49501A.12.1.2rr — SPW-SA-495-01 A — 1H COSY @ 298K — 8 mg CDCl3 av600neo cryoBBO — 06/10/2021

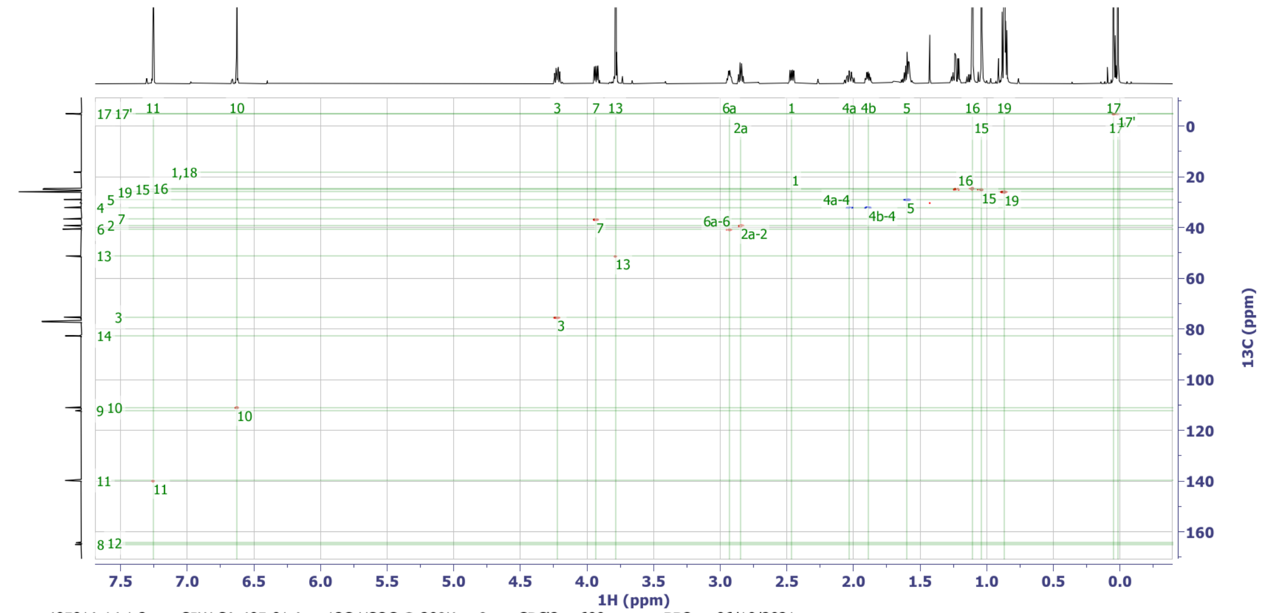

spwsa49501A.14.1.2rr — SPW-SA-495-01 A — 13C-HSQC @ 298K — 8 mg CDCl3 av600neo cryoBBO — 06/10/2021

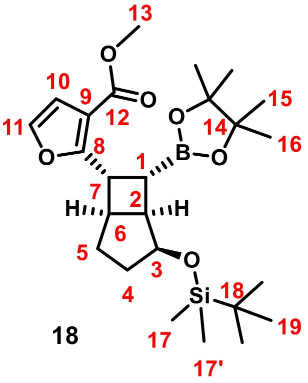

18

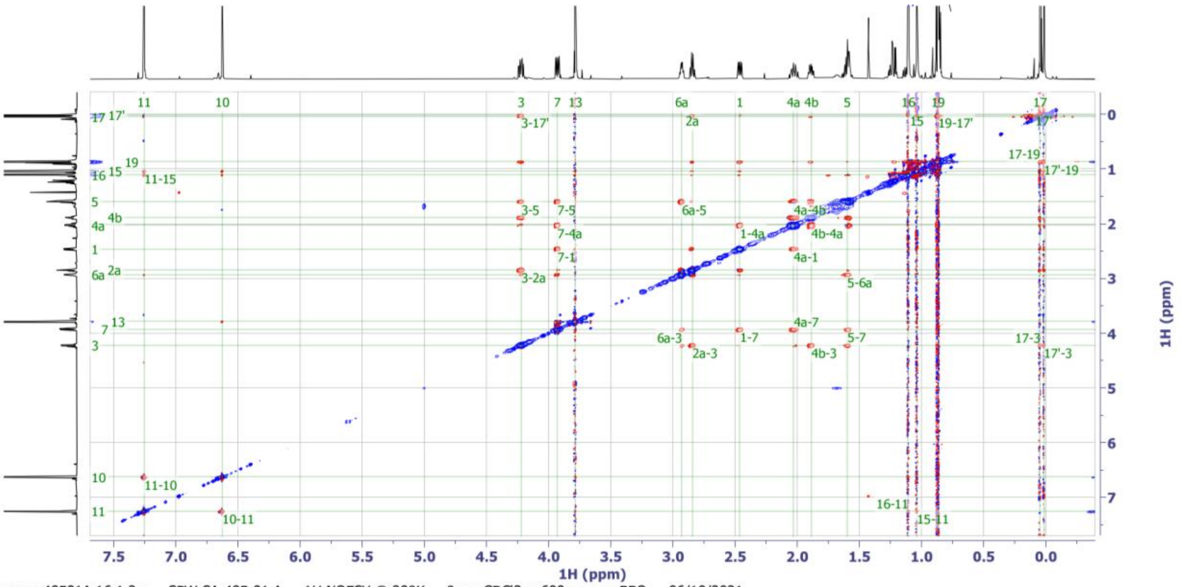

spwsa49501A.16.1.2rr — SPW-SA-495-01 A — 1H NOESY @ 298K — 8 mg CDCl3 av600neo cryoBBO — 06/10/2021

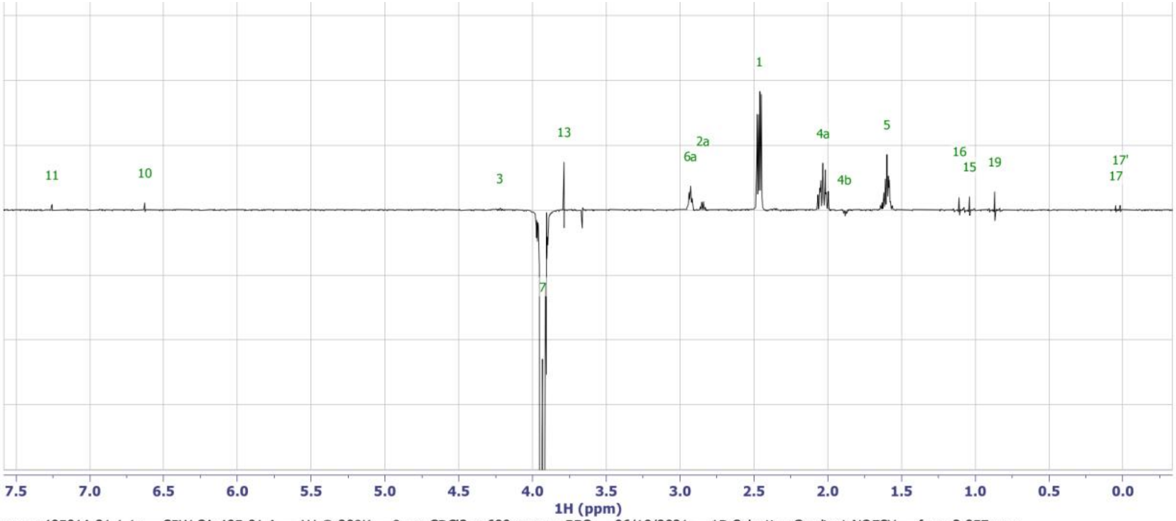

spwsa49501A.21.1.1r — SPW-SA-495-01 A — 1H @ 298K — 8 mg CDCl3 av600neo cryoBBO — 06/10/2021 — 1D Selective Gradient NOESY — freq: 3.957ppm

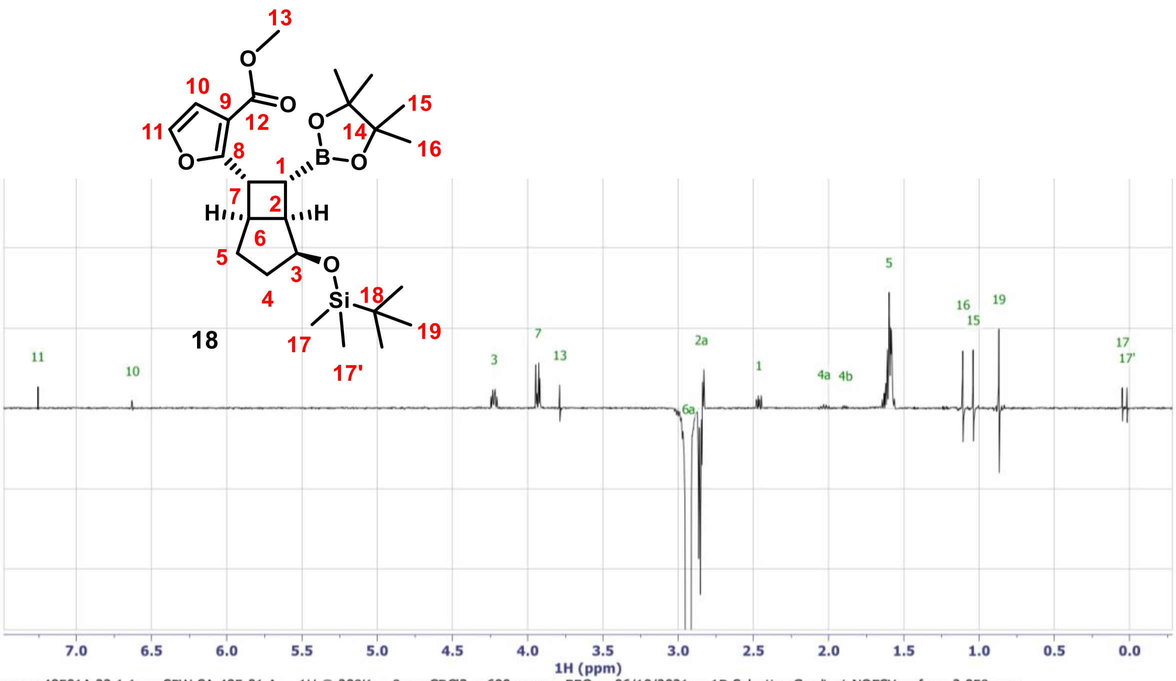

spwsa49501A.22.1.1r — SPW-SA-495-01 A — 1H @ 298K — 8 mg CDCl3 av600neo cryoBBO — 06/10/2021 — 1D Selective Gradient NOESY — freq: 2.958ppm

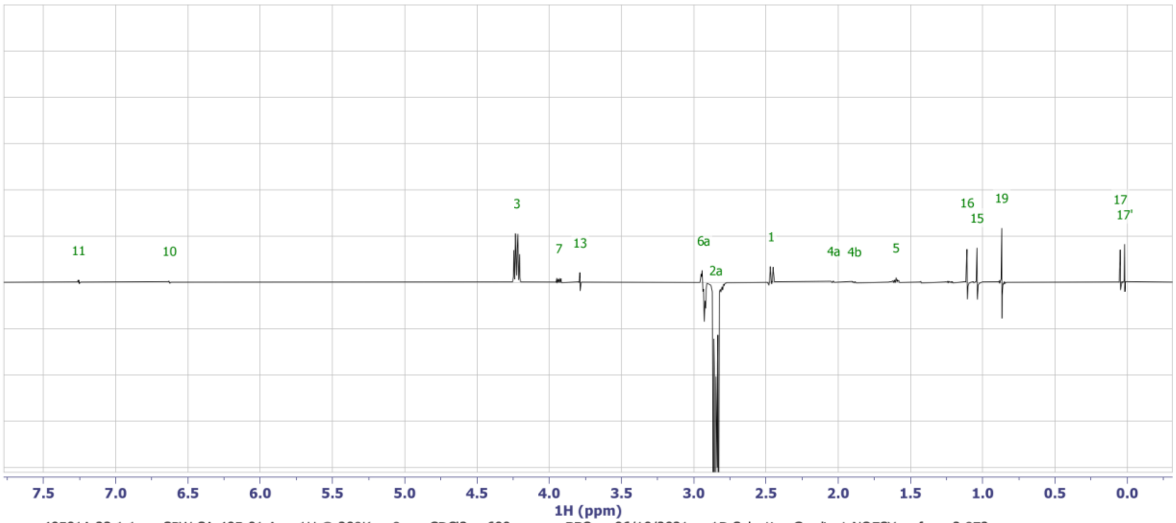

spwsa49501A.23.1.1r — SPW-SA-495-01 A — 1H @ 298K — 8 mg CDCl3 av600neo cryoBBO — 06/10/2021 — 1D Selective Gradient NOESY — freq: 2.872ppm

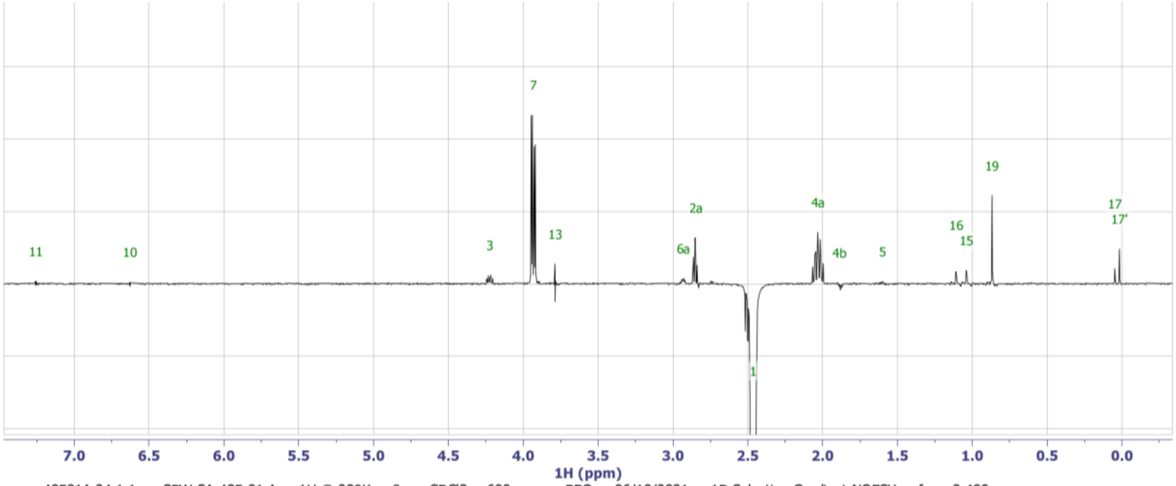

spwsa49501A.24.1.1r — SPW-SA-495-01 A — 1H @ 298K — 8 mg CDCl3 av600neo cryoBBO — 06/10/2021 — 1D Selective Gradient NOESY — freq: 2.490ppm

1H (CDCl3, 600.20 MHz)

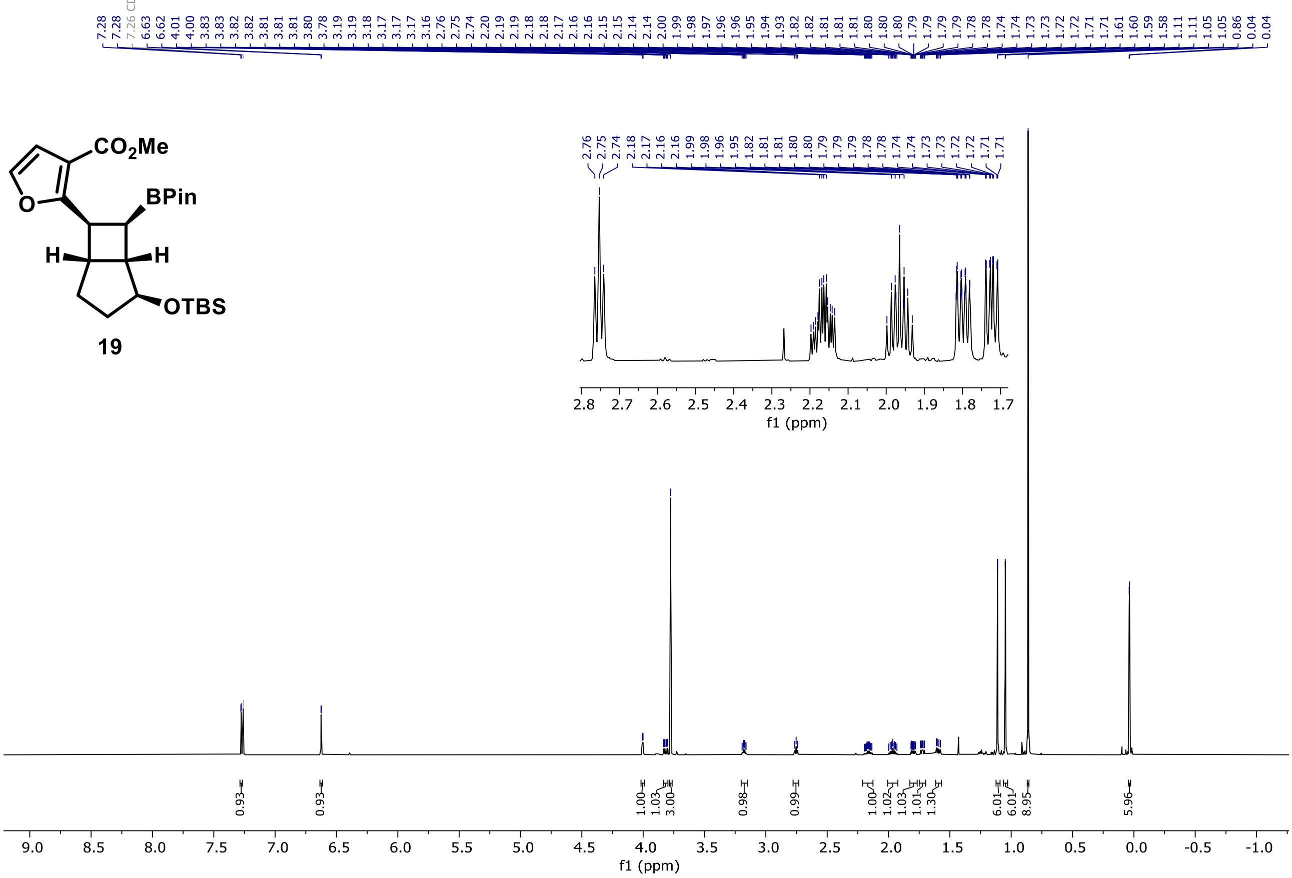

13C (CDCl3, 150.94 MHz)

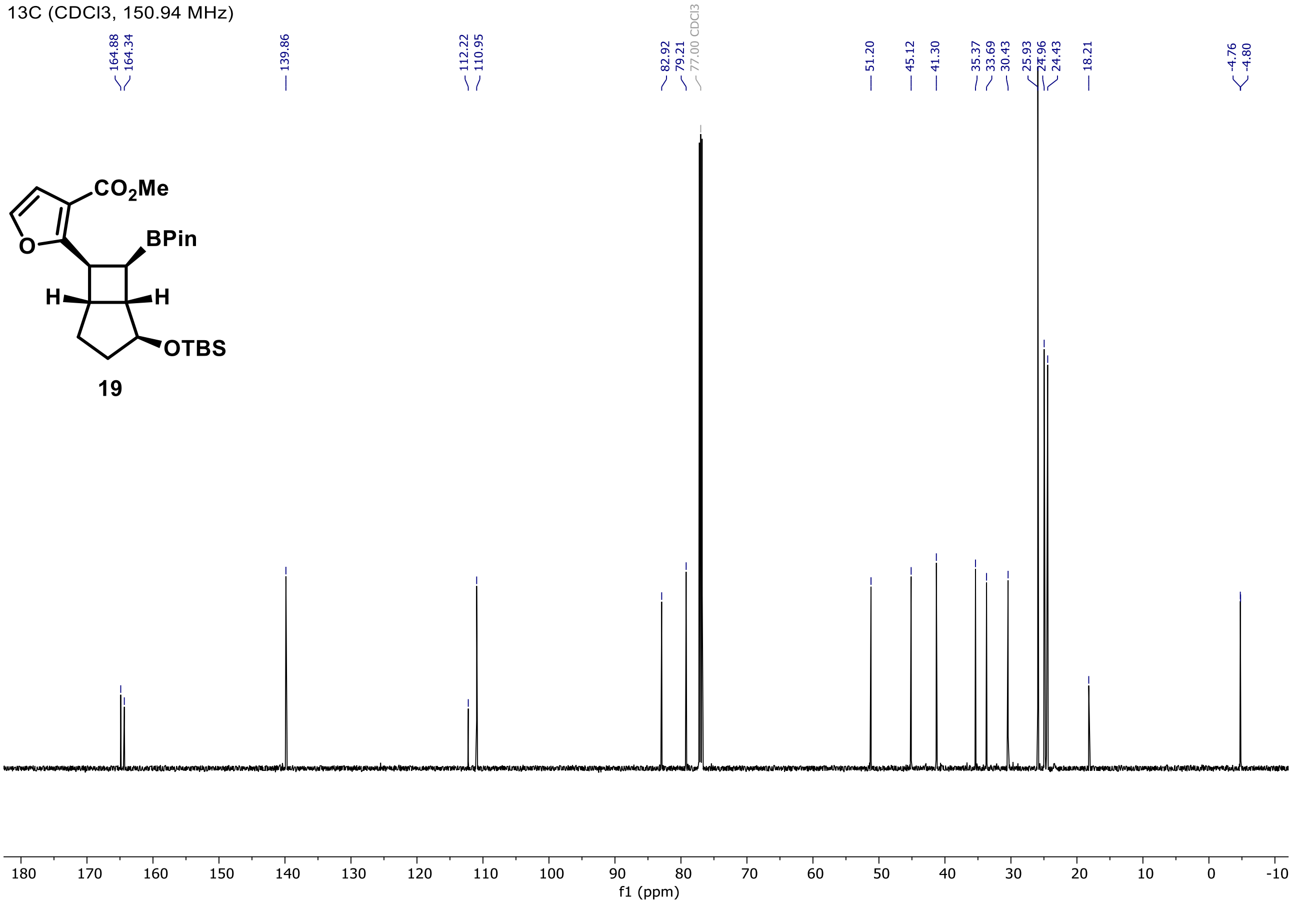

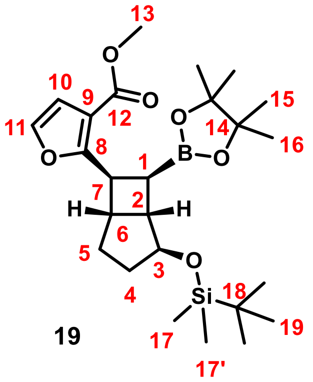

19

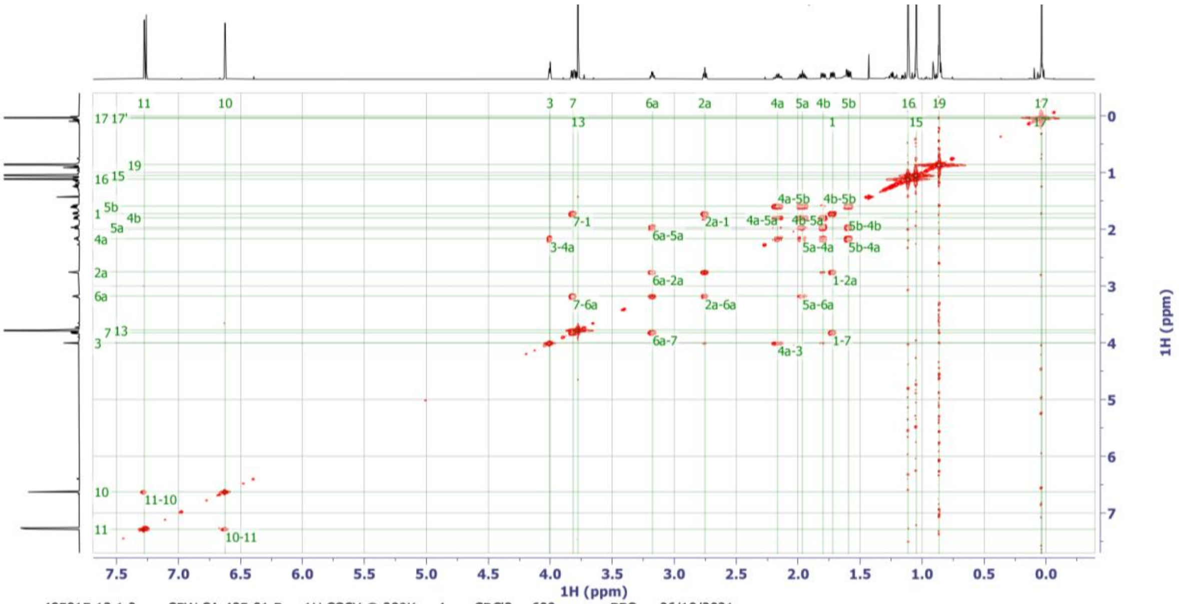

spwsa49501B.12.1.2rr — SPW-SA-495-01 B — 1H COSY @ 298K — 4 mg CDCl3 av600neo cryoBBO — 06/10/2021

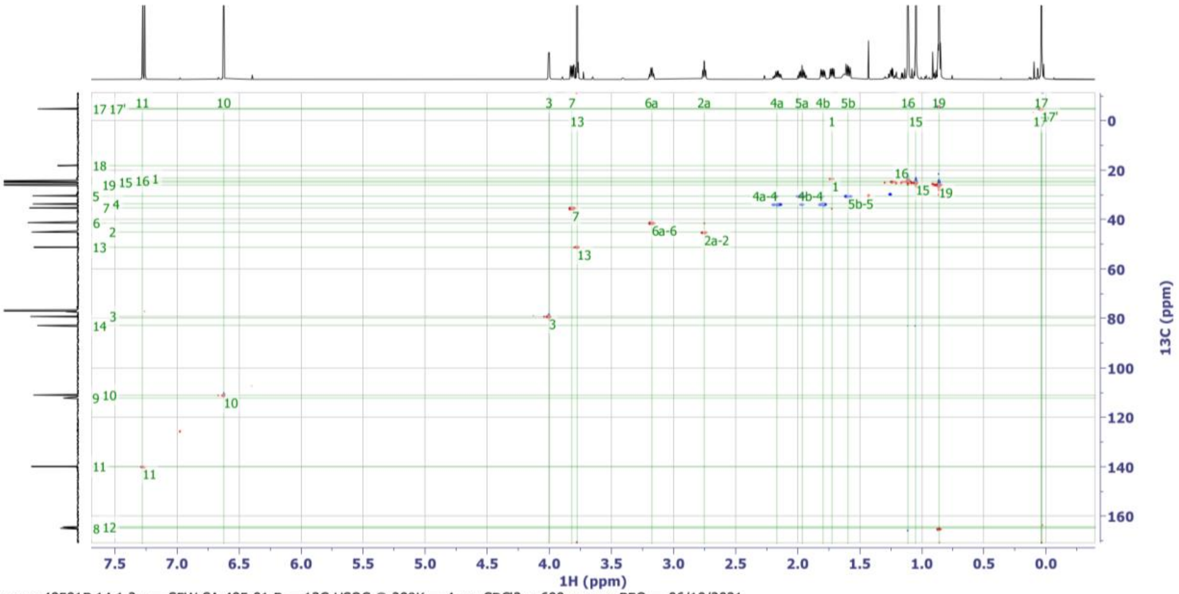

spwsa49501B.14.1.2rr — SPW-SA-495-01 B — 13C-HSQC @ 298K — 4 mg CDCl3 av600neo cryoBBO — 06/10/2021

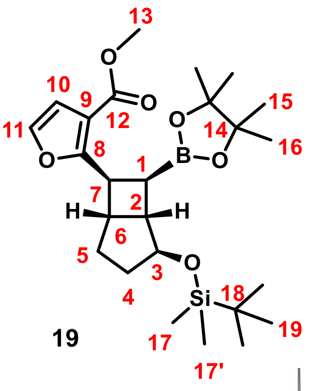

19

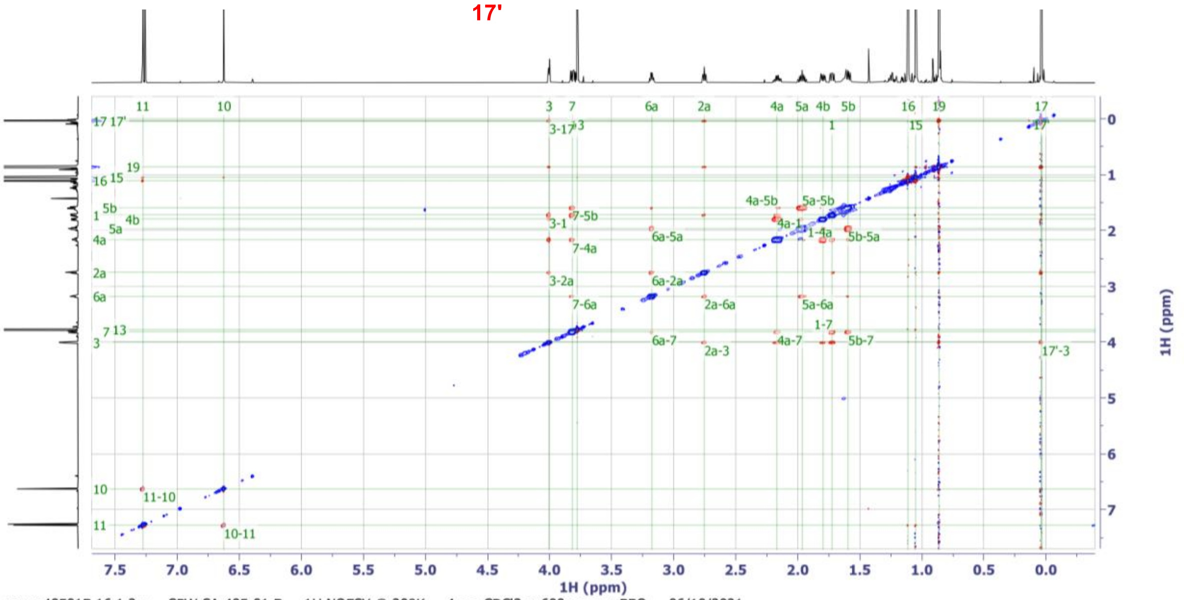

spwsa49501B.16.1.2rr — SPW-SA-495-01 B — 1H NOESY @ 298K — 4 mg CDCl3 av600neo cryoBBO — 06/10/2021

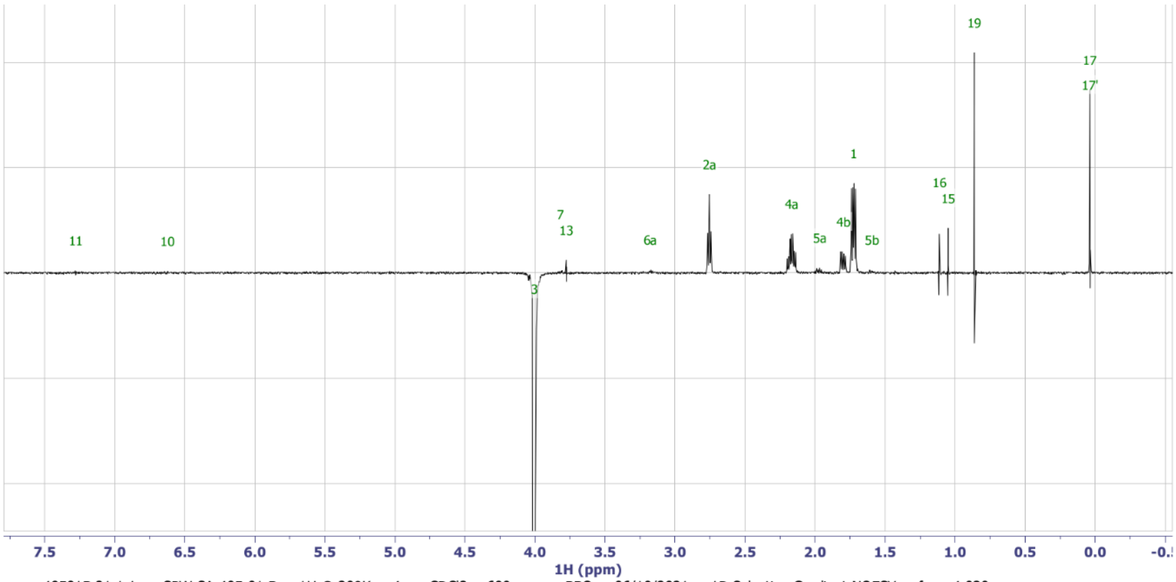

spwsa49501B.21.1.1r — SPW-SA-495-01 B — 1H @ 298K — 4 mg CDCl3 av600neo cryoBBO — 06/10/2021 — 1D Selective Gradient NOESY — freq: 4.030ppm

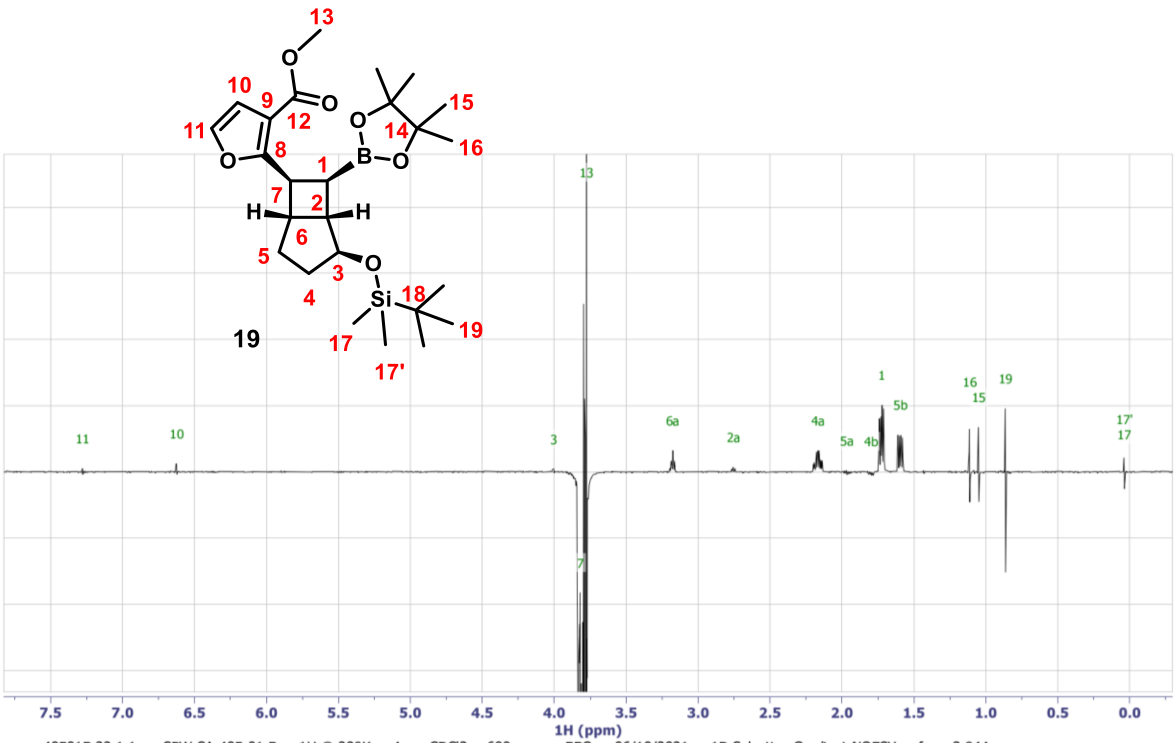

spwsa49501B.22.1.1r — SPW-SA-495-01 B — 1H @ 298K — 4 mg CDCl3 av600neo cryoBBO — 06/10/2021 — 1D Selective Gradient NOESY — freq: 3.844ppm

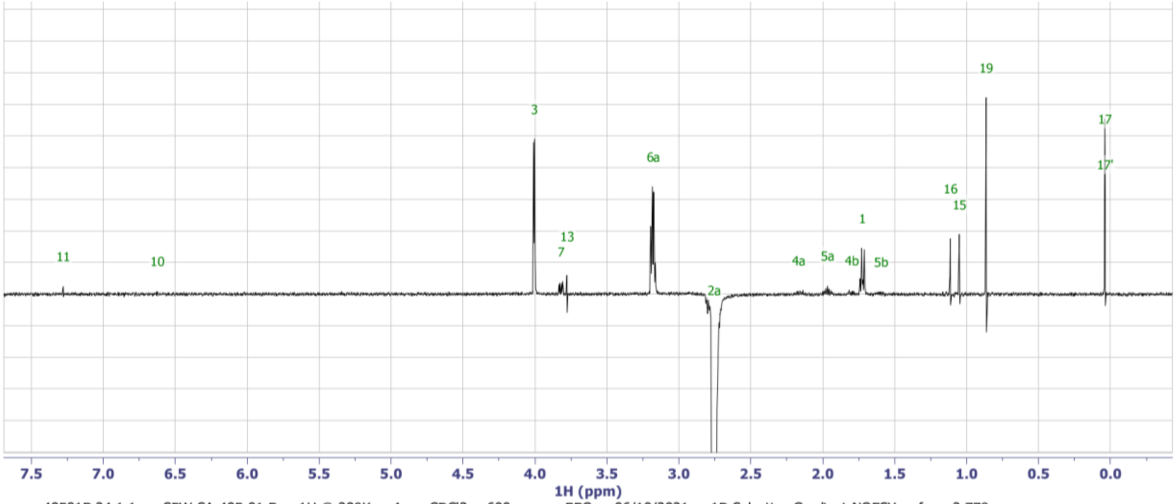

spwsa49501B.24.1.1r — SPW-SA-495-01 B — 1H @ 298K — 4 mg CDCl3 av600neo cryoBBO — 06/10/2021 — 1D Selective Gradient NOESY — freq: 2.778ppm

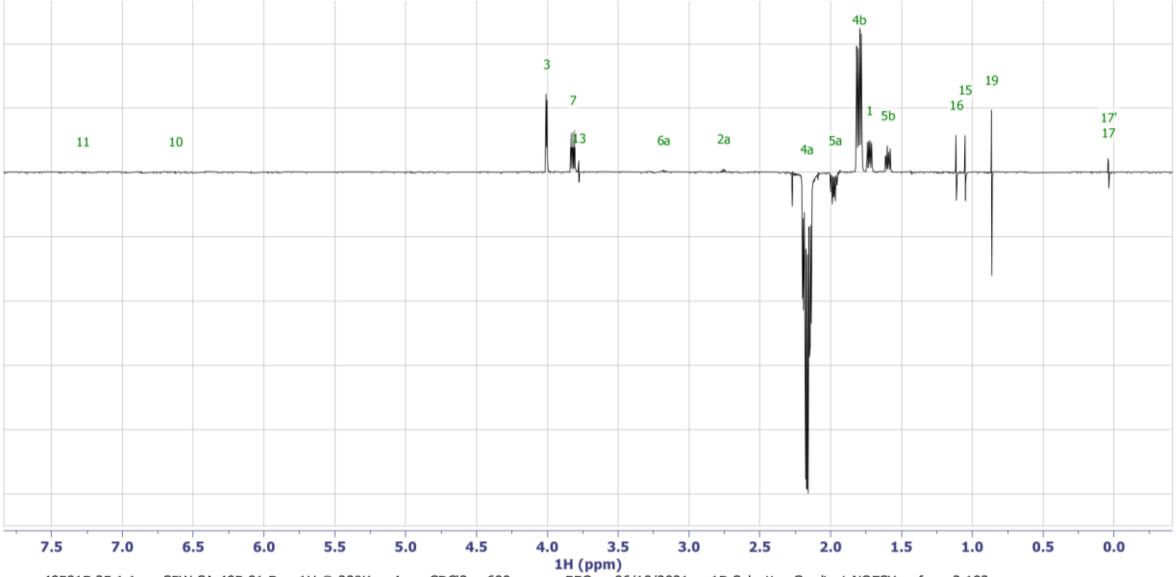

spwsa49501B.25.1.1r — SPW-SA-495-01 B — 1H @ 298K — 4 mg CDCl3 av600neo cryoBBO — 06/10/2021 — 1D Selective Gradient NOESY — freq: 2.192ppm

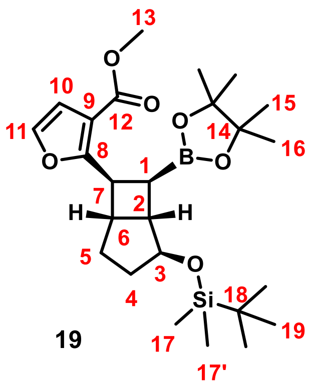

19

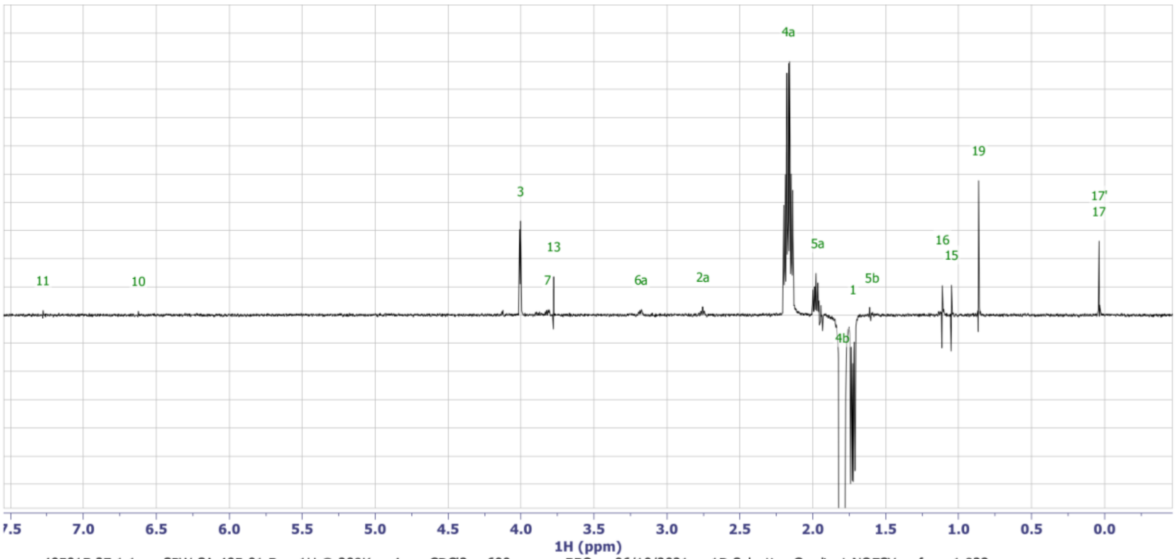

spwsa49501B.27.1.1r — SPW-SA-495-01 B — 1H @ 298K — 4 mg CDCl3 av600neo cryoBBO — 06/10/2021 — 1D Selective Gradient NOESY — freq: 1.823ppm

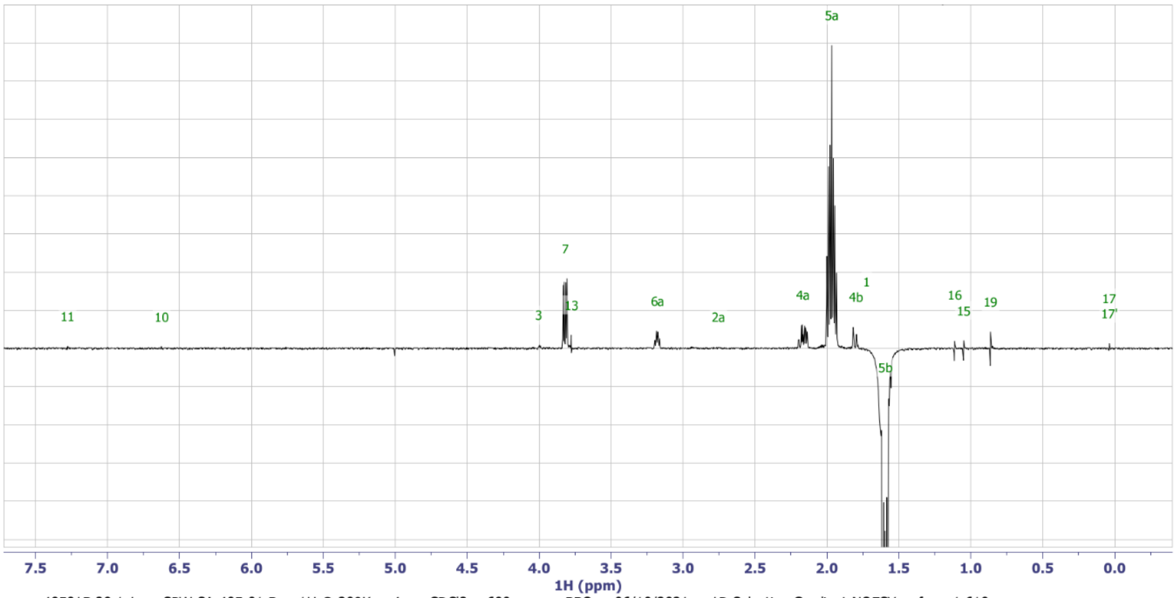

spwsa49501B.29.1.1r — SPW-SA-495-01 B — 1H @ 298K — 4 mg CDCl3 av600neo cryoBBO — 06/10/2021 — 1D Selective Gradient NOESY — freq: 1.619ppm

1H (CDCl3, 400.12 MHz)

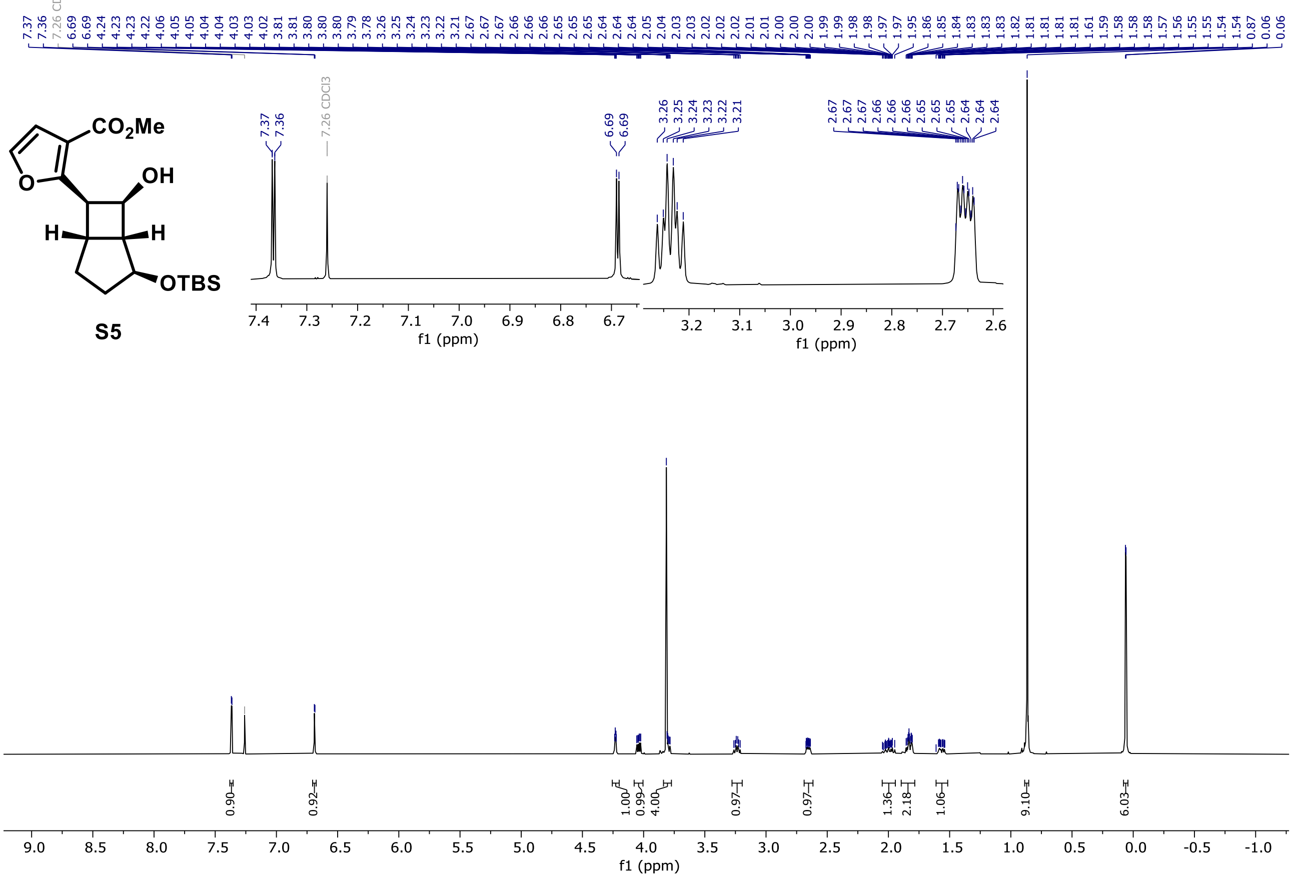

13C (CDCl3, 100.62 MHz)

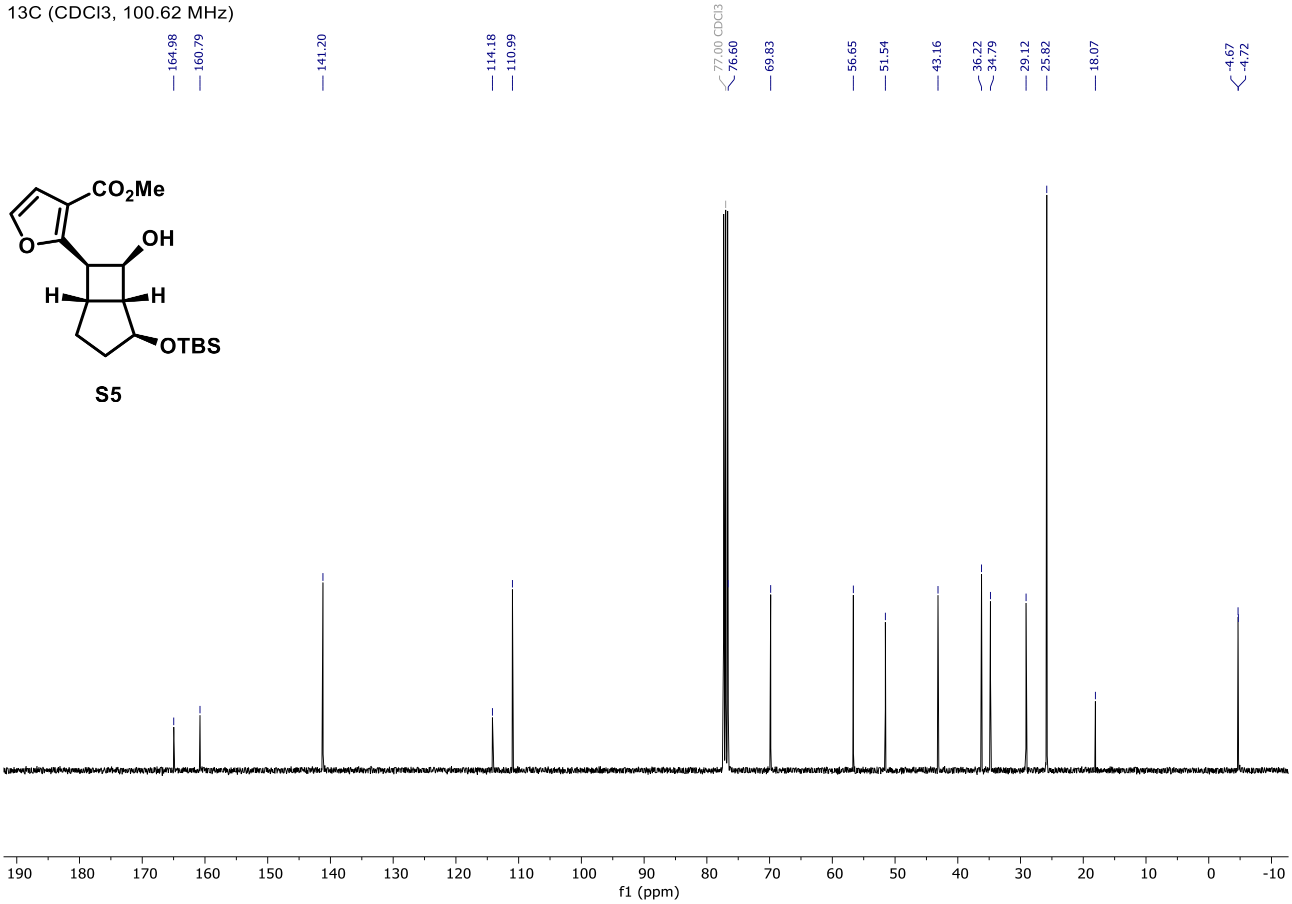

1H (CDCl3, 400.12 MHz)

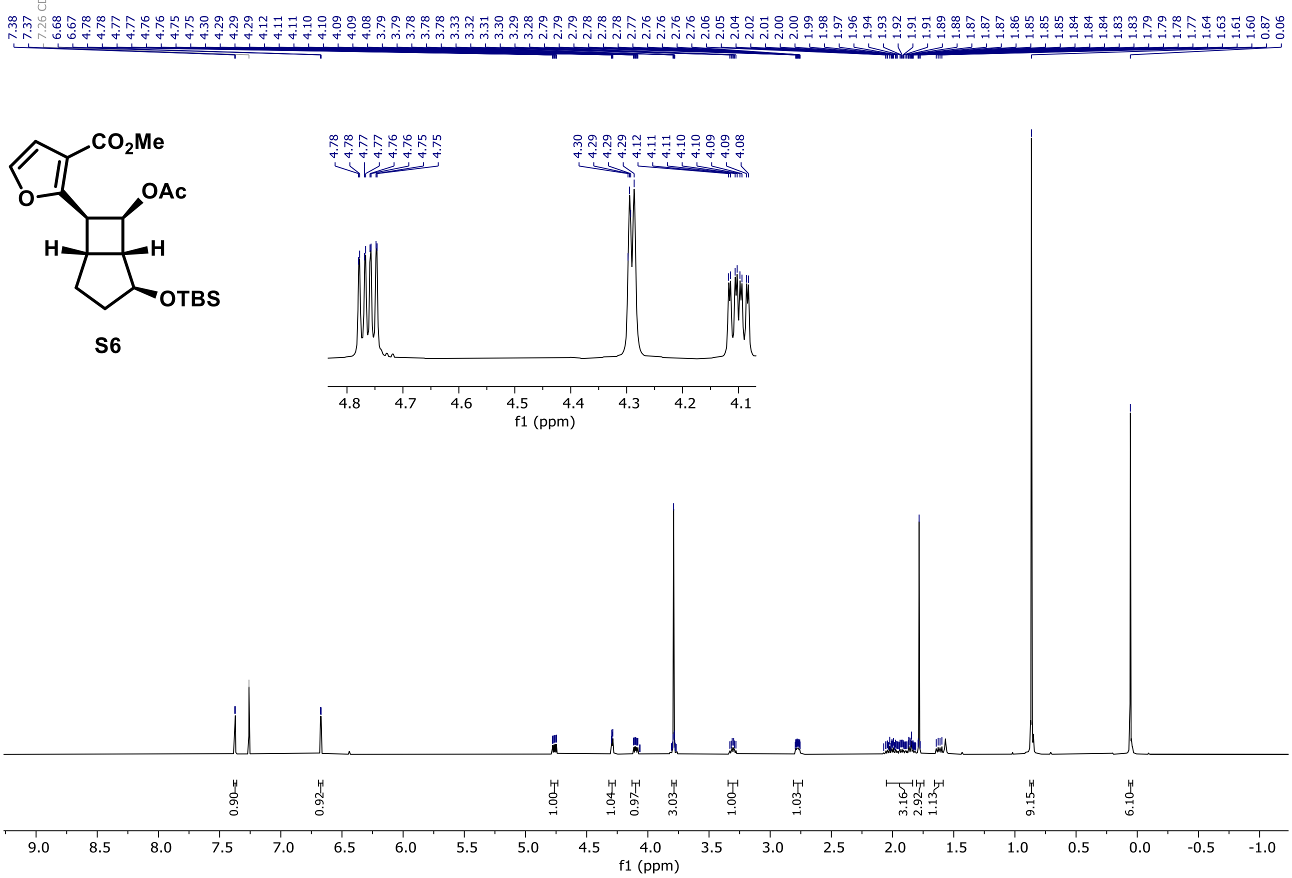

13C (CDCl3, 100.62 MHz)

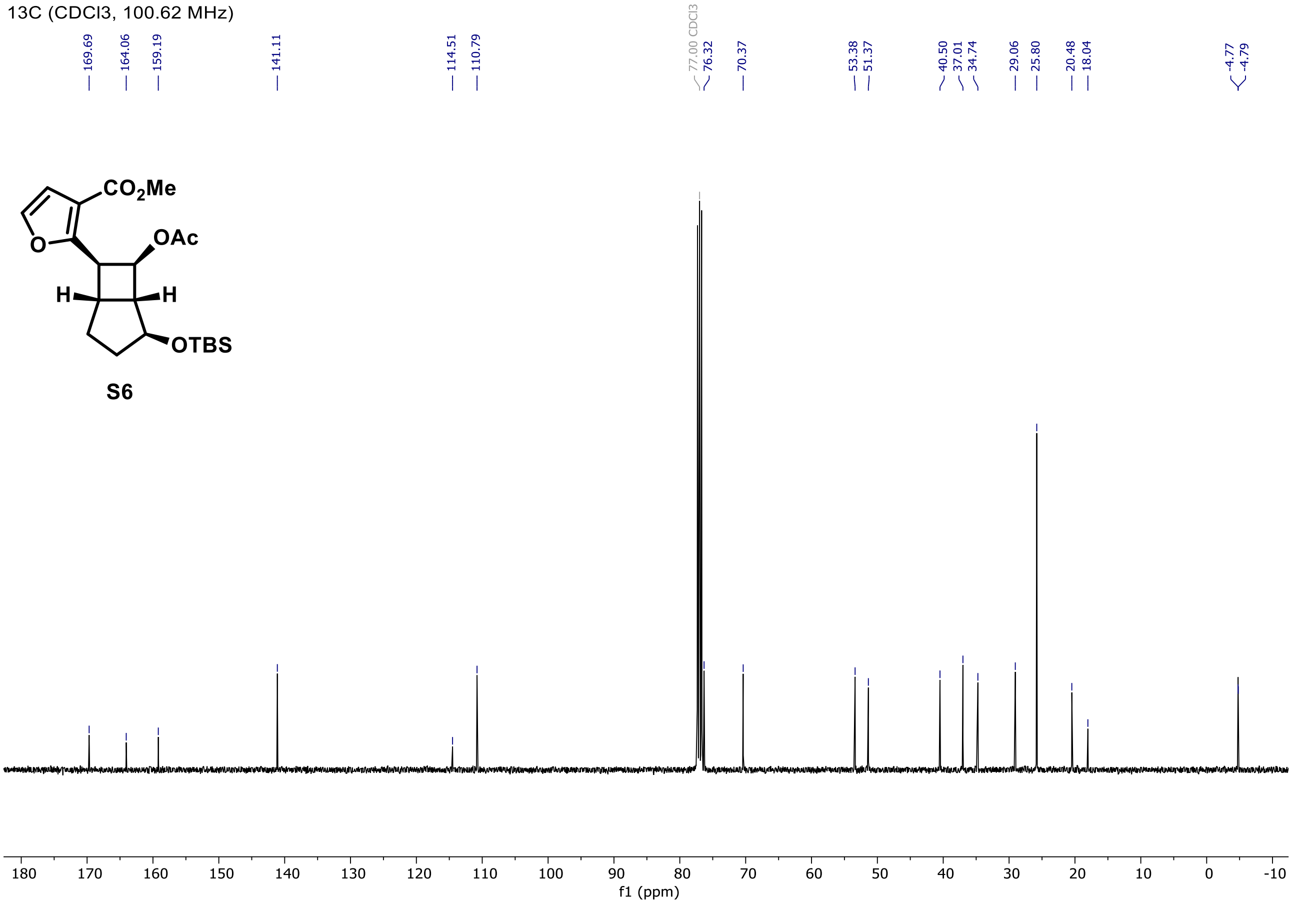

1H (CDCl3, 400.12 MHz)

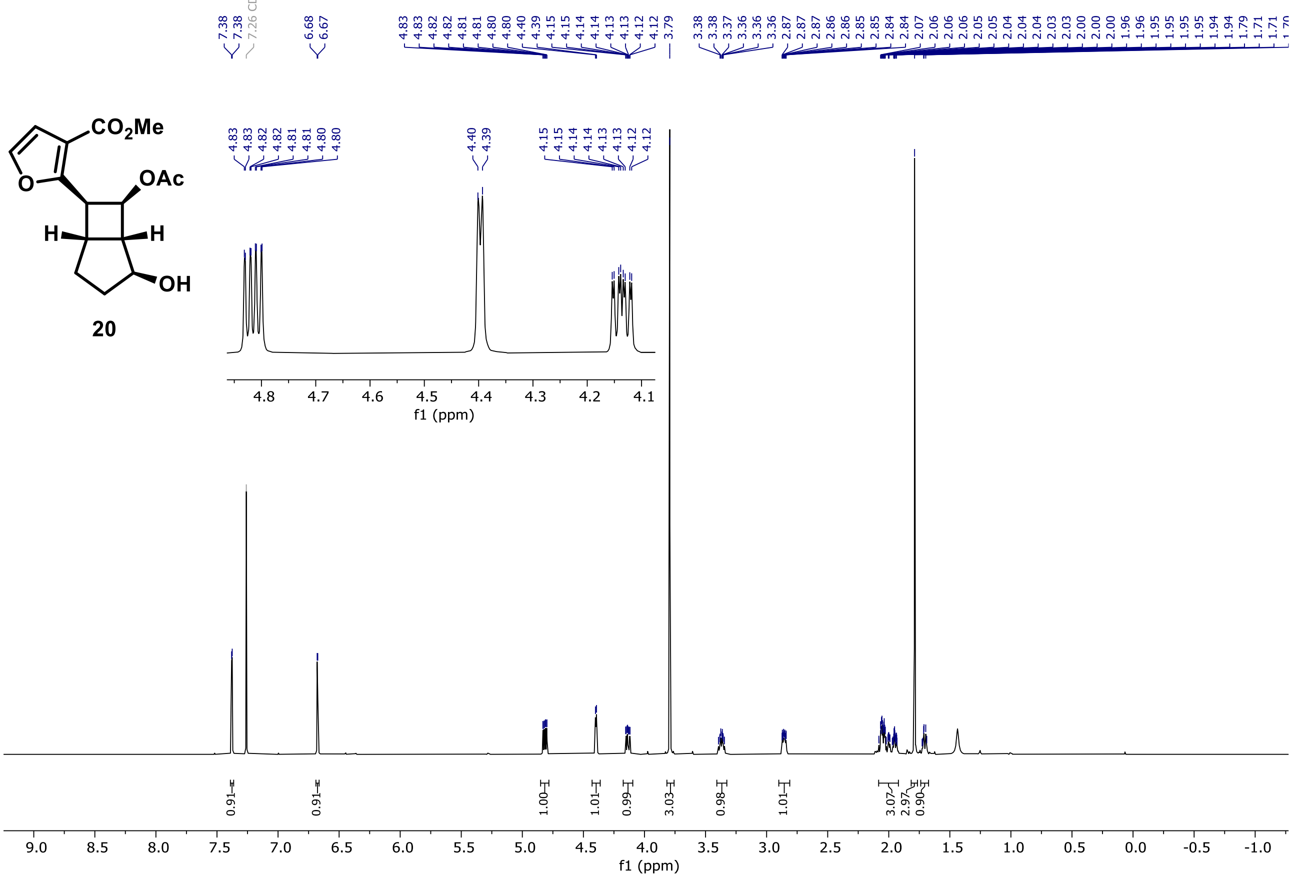

13C (CDCl3, 100.62 MHz)

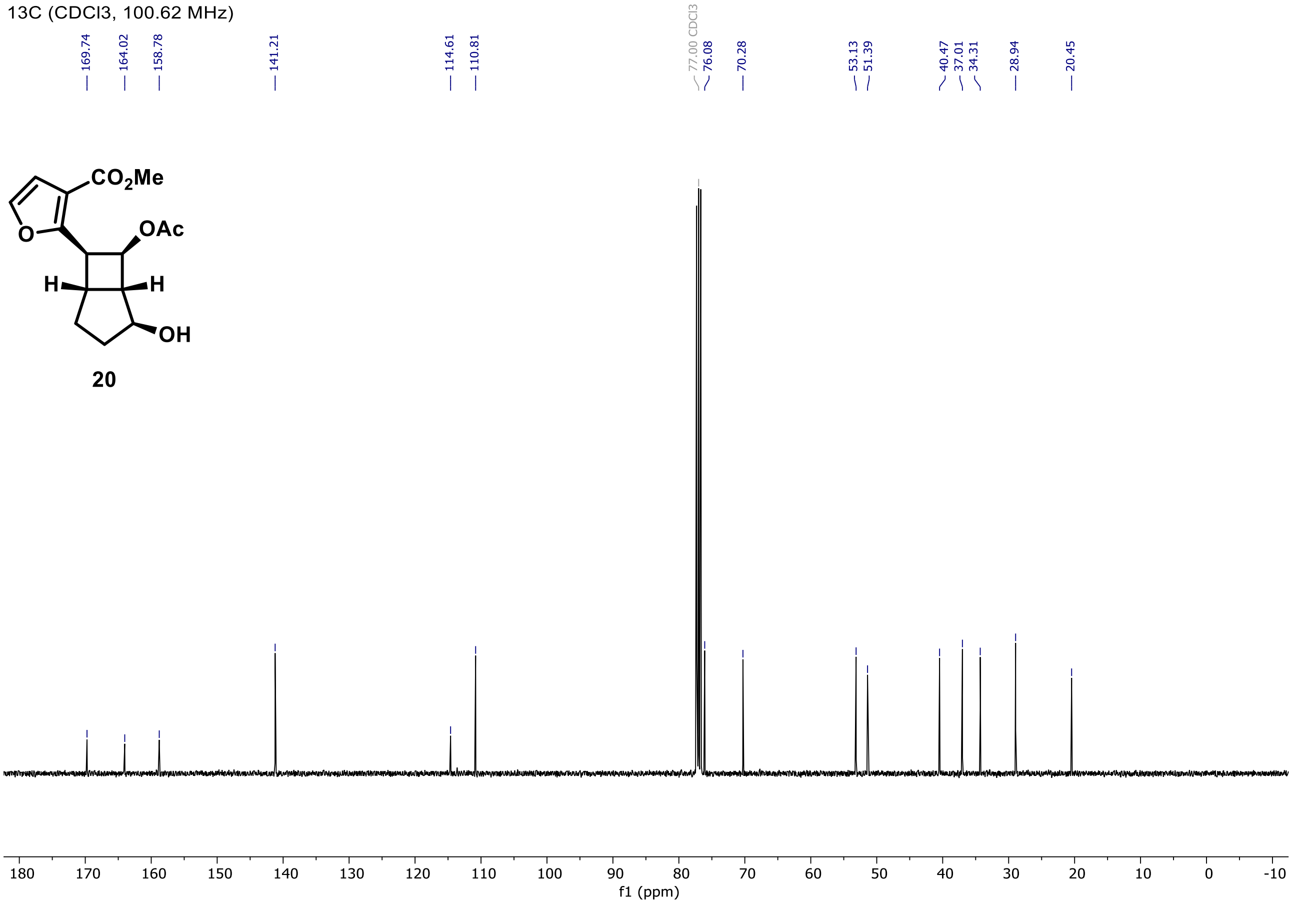

1H (CDCl3, 400.12 MHz)

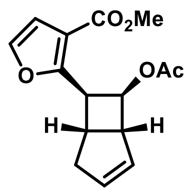

21

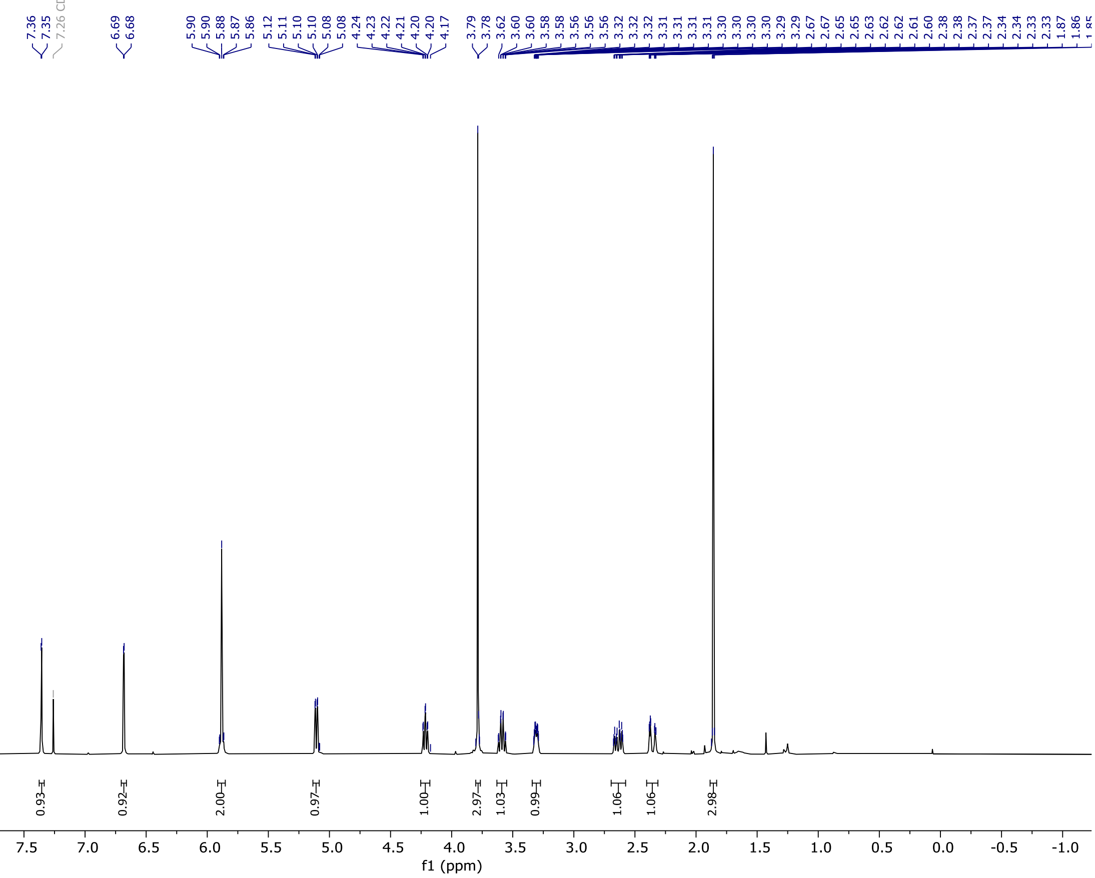

13C (CDCl3, 100.62 MHz)

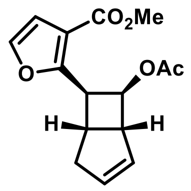

21

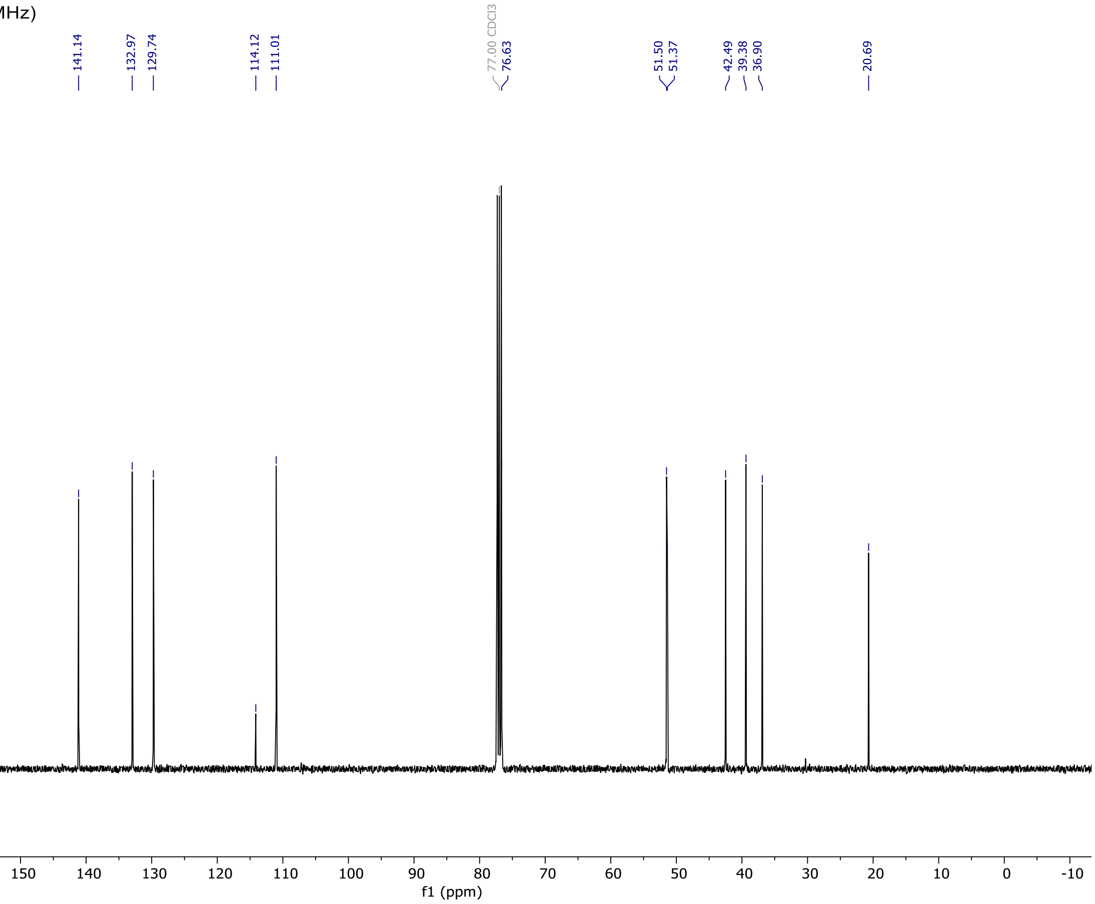

1H (CDCl3, 400.12 MHz)

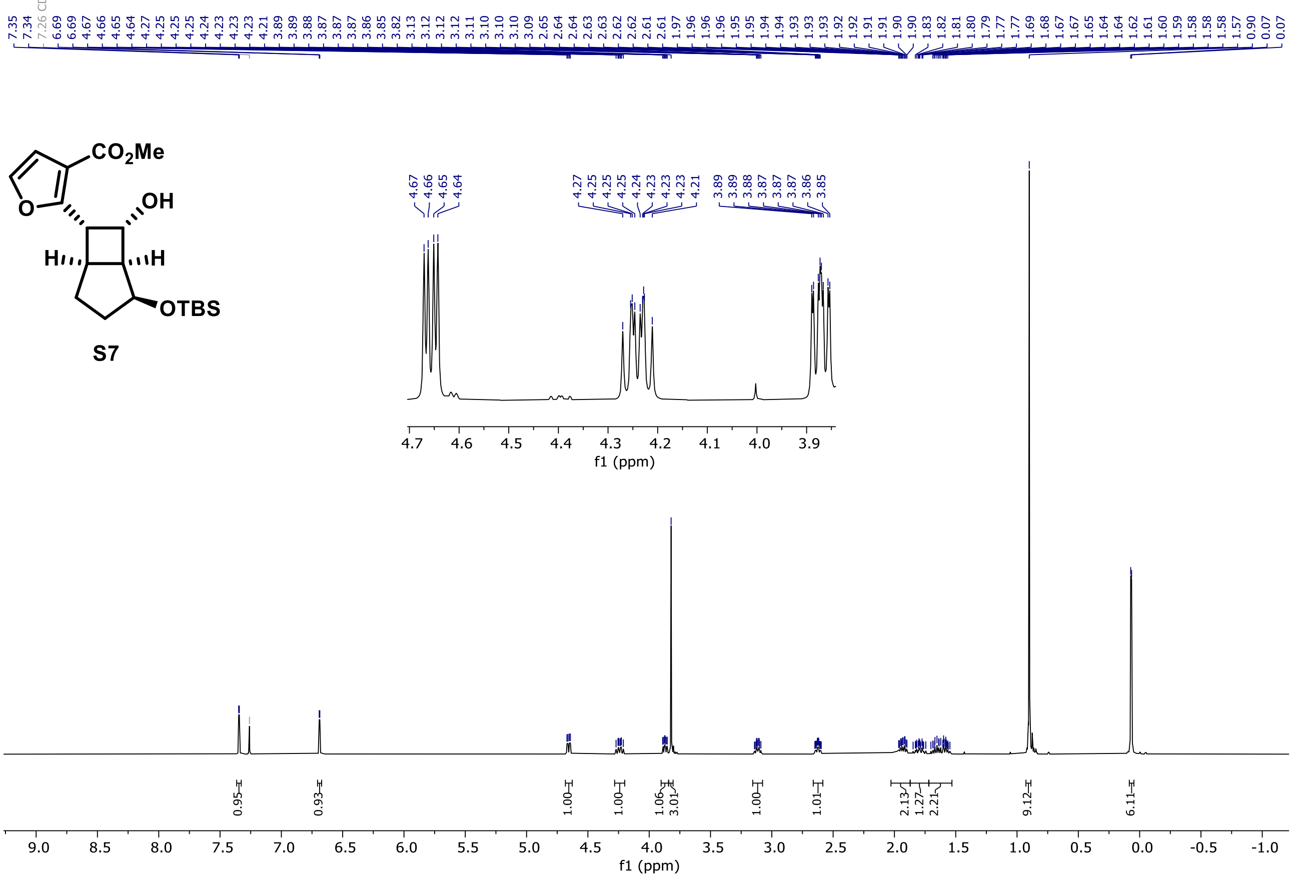

13C (CDCl3, 100.62 MHz)

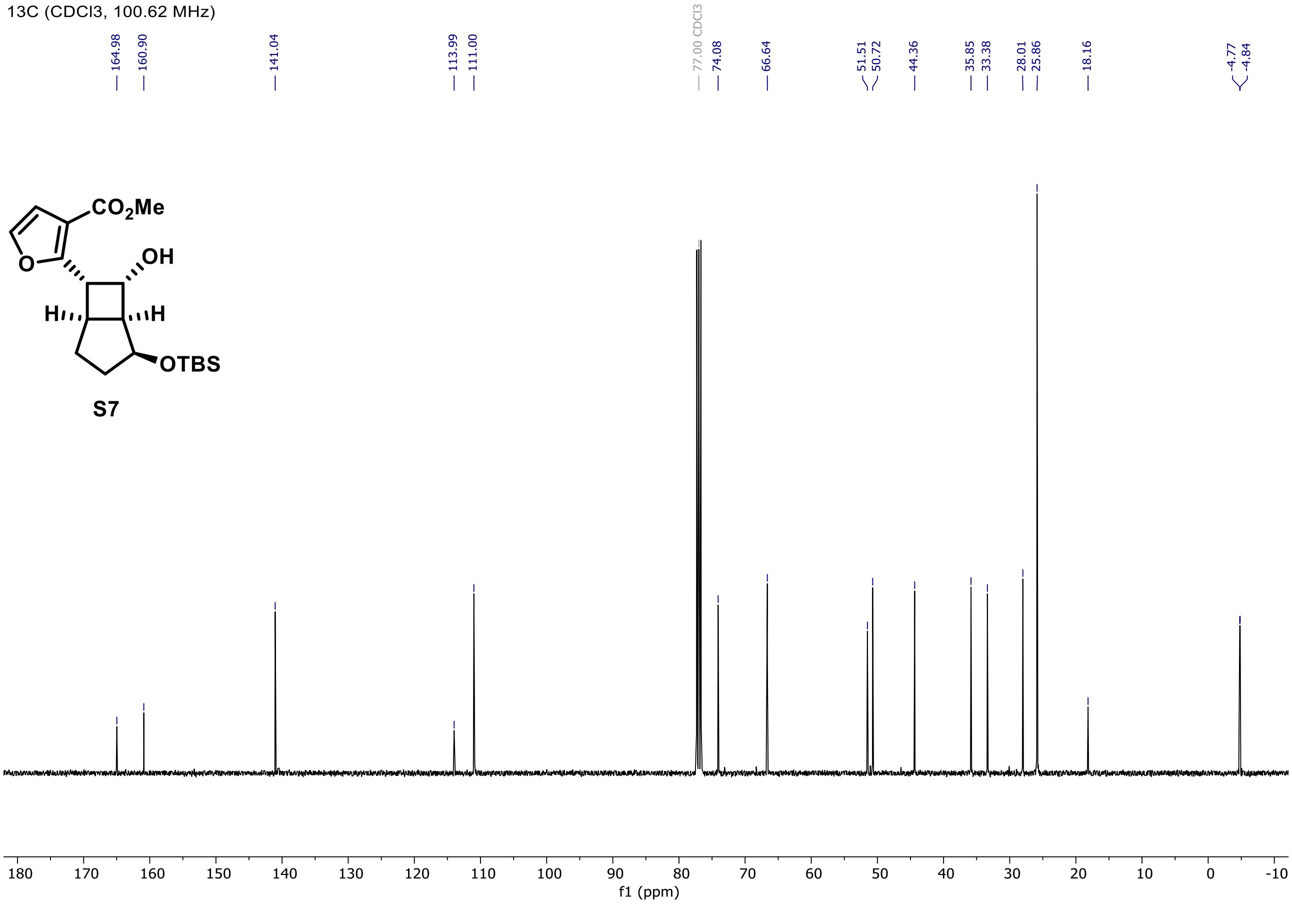

1H (CDCl3, 400.12 MHz)

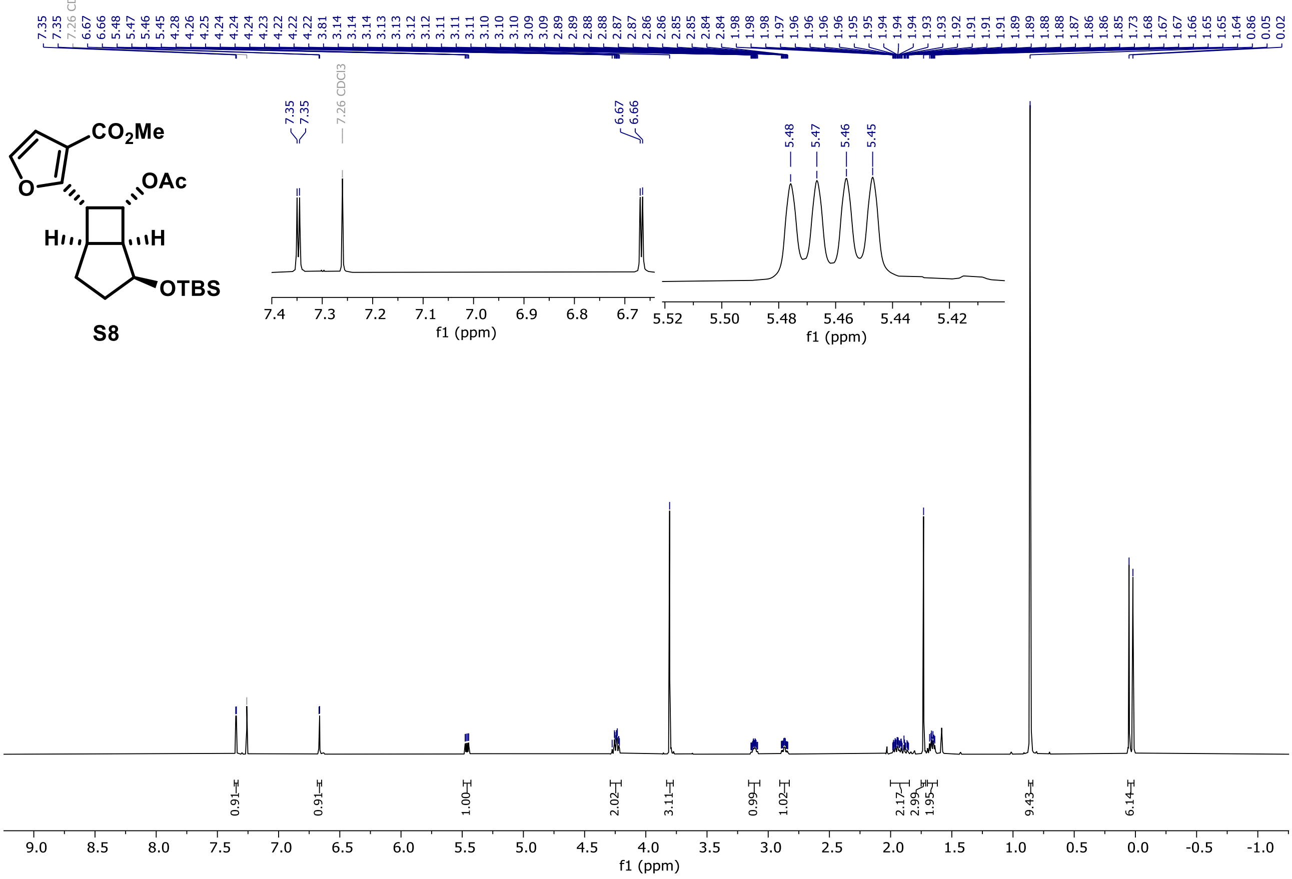

13C (CDCl3, 100.62 MHz)

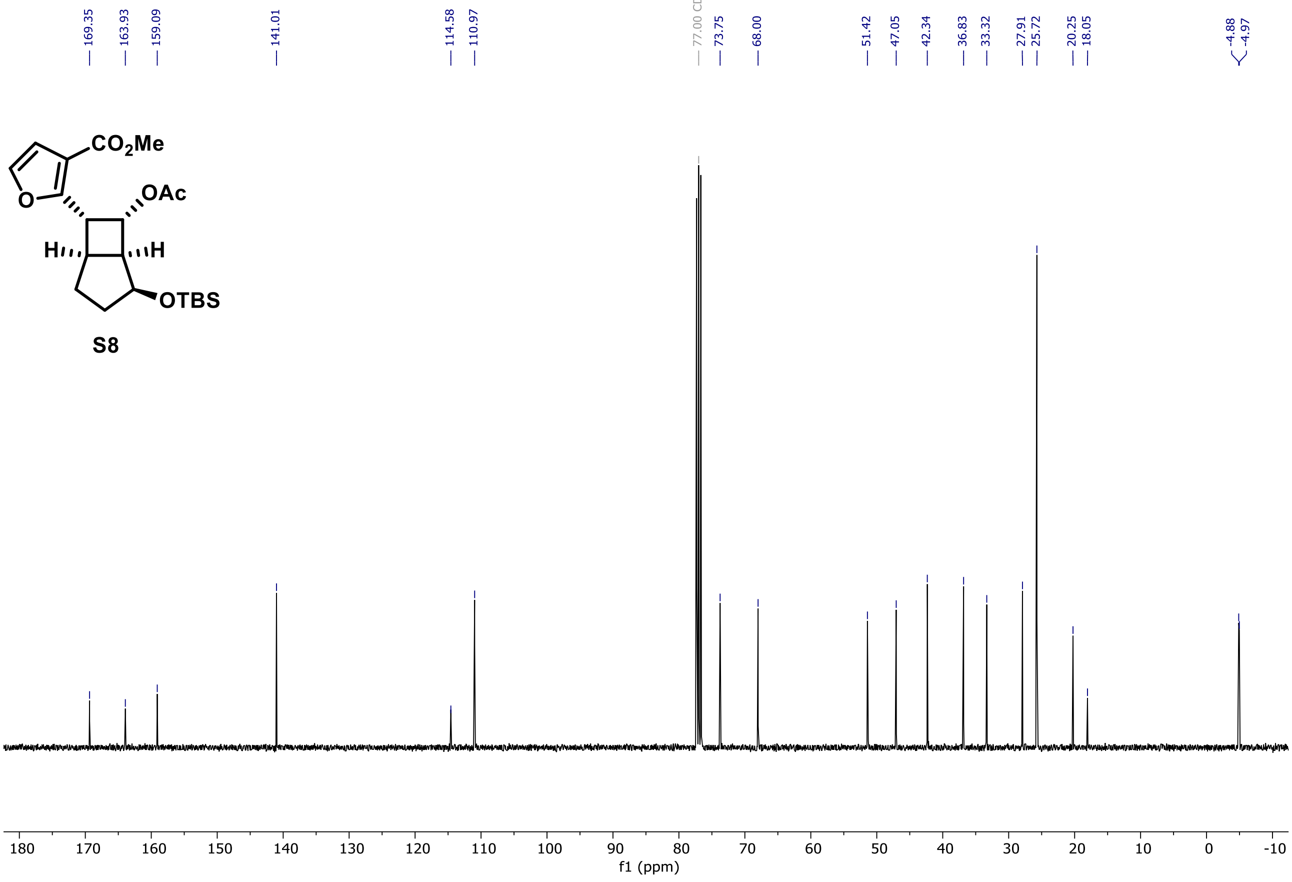

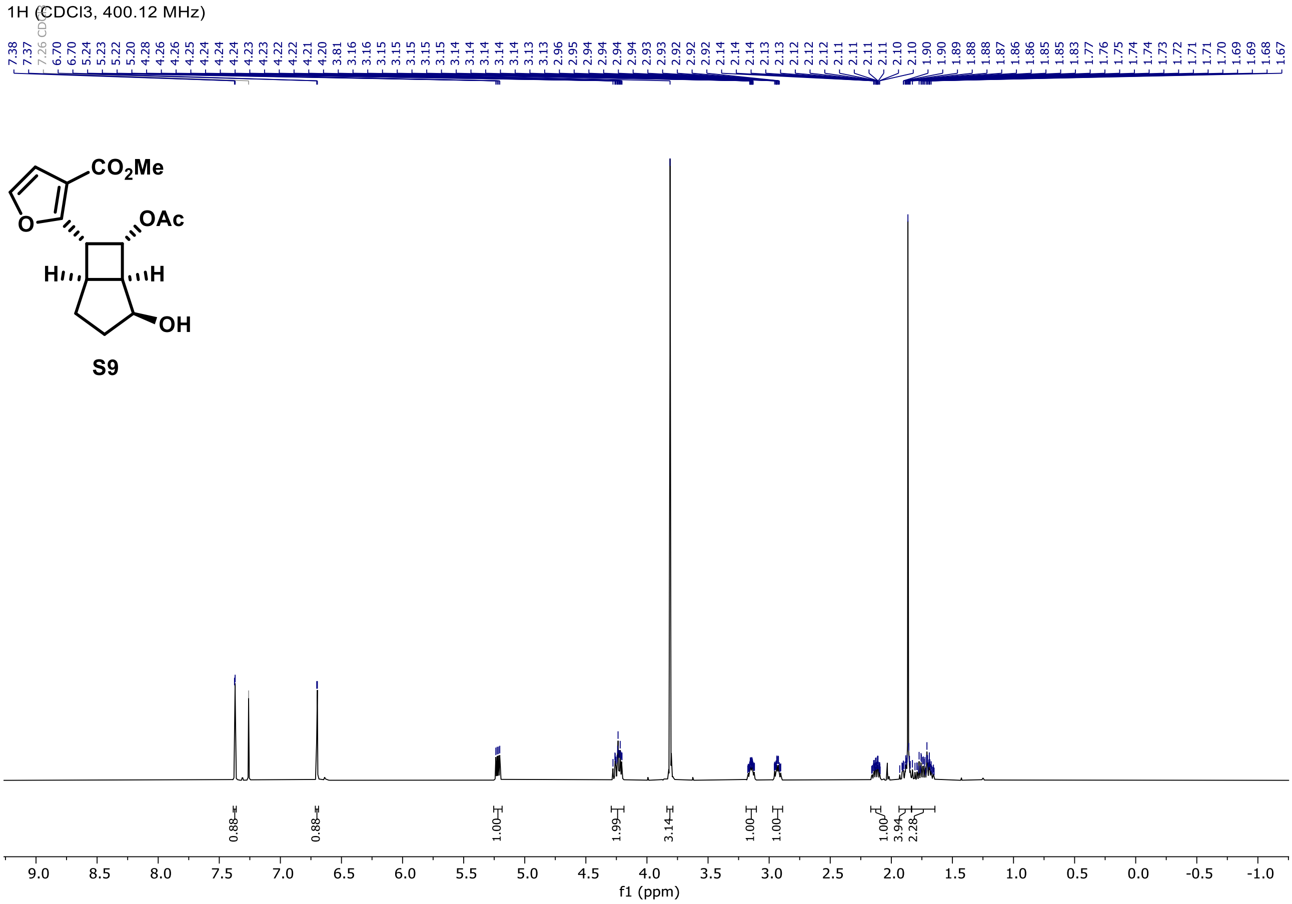
1H (CDCl3, 400.12 MHz)
7.38 7.37 7.26 CDCl3 6.70 6.70 5.24 5.23 5.22 5.20 4.28 4.26 4.26 4.25 4.24 4.24 4.24 4.23 4.23 4.22 4.22 4.21 4.20 3.81 3.16 3.16 3.15 3.15 3.15 3.15 3.14 3.14 3.14 3.14 3.14 3.13 3.13 2.96 2.95 2.94 2.94 2.94 2.94 2.93 2.93 2.92 2.92 2.92 2.14 2.14 2.14 2.13 2.13 2.12 2.12 2.12 2.11 2.11 2.11 2.11 2.10 2.10 1.90 1.90 1.89 1.88 1.88 1.87 1.86 1.86 1.85 1.85 1.83 1.77 1.76 1.75 1.74 1.74 1.73 1.72 1.71 1.71 1.70 1.69 1.69 1.68 1.67
CO2Me
OAc
O
H
H
OH
S9
0.88 0.88 1.00 1.99 3.14 1.00 1.00 1.00 3.94 2.28
9.0 8.5 8.0 7.5 7.0 6.5 6.0 5.5 5.0 4.5 4.0 3.5 3.0 2.5 2.0 1.5 1.0 0.5 0.0 -0.5 -1.0
f1 (ppm)

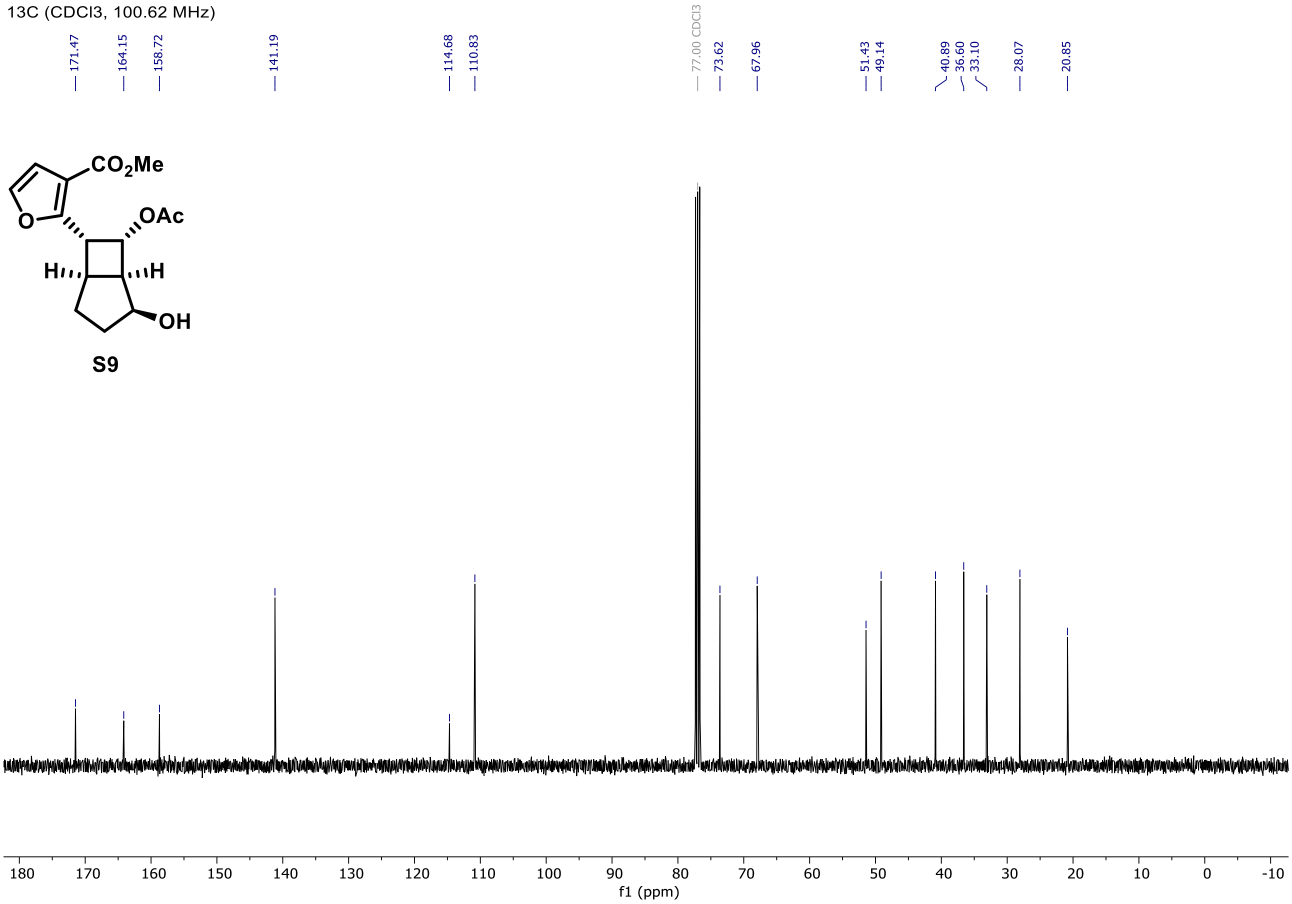
13C (CDCl3, 100.62 MHz)
171.47 164.15 158.72 141.19 114.68 110.83 77.00 CDCl3 73.62 67.96 51.43 49.14 40.89 36.60 33.10 28.07 20.85
CO2Me
OAc
O
H
H
OH
S9
180 170 160 150 140 130 120 110 100 90 80 70 60 50 40 30 20 10 0 -10
f1 (ppm)

1H (CDCl3, 400.12 MHz)

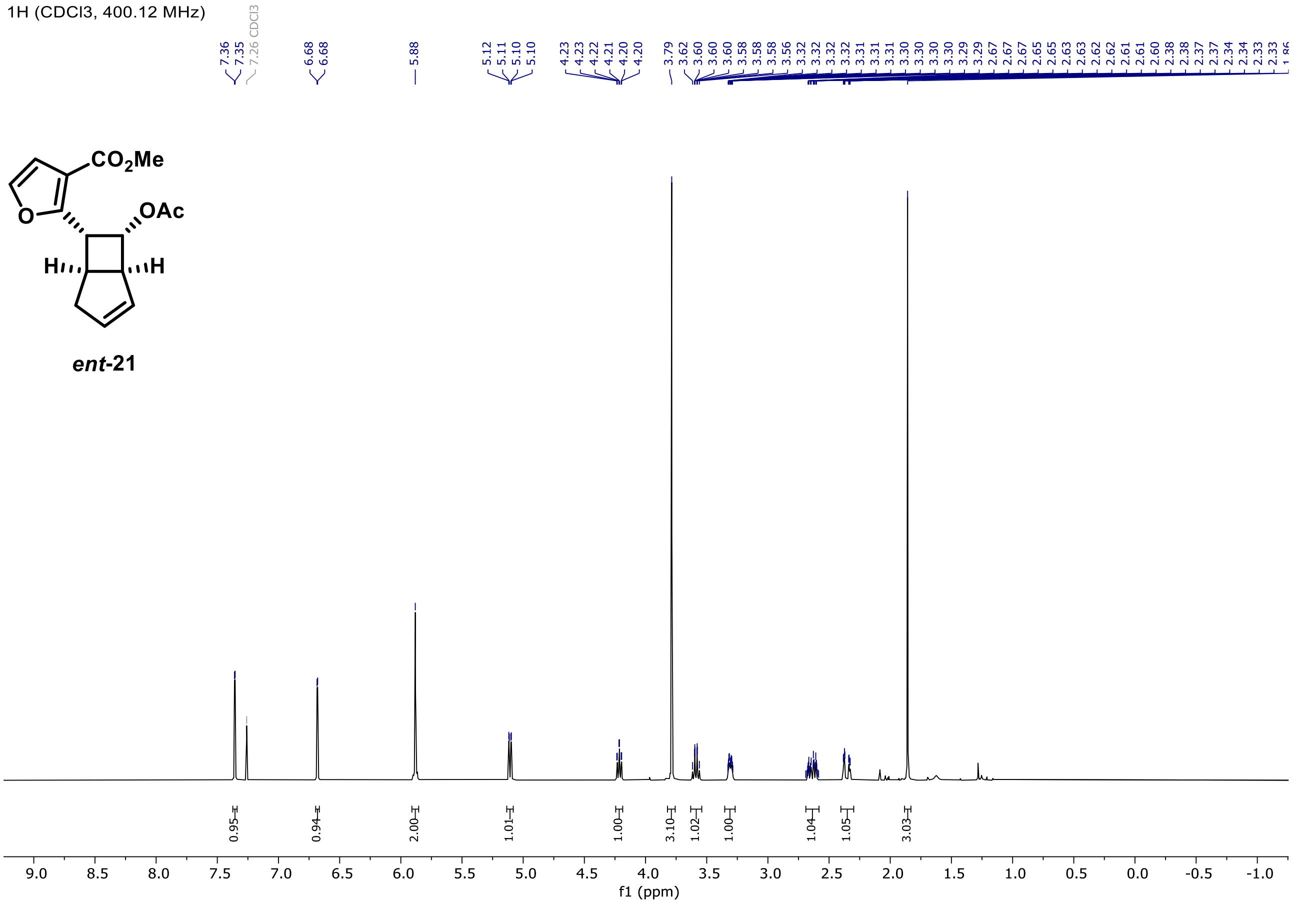

ent-21

13C (CDCl3, 100.62 MHz)

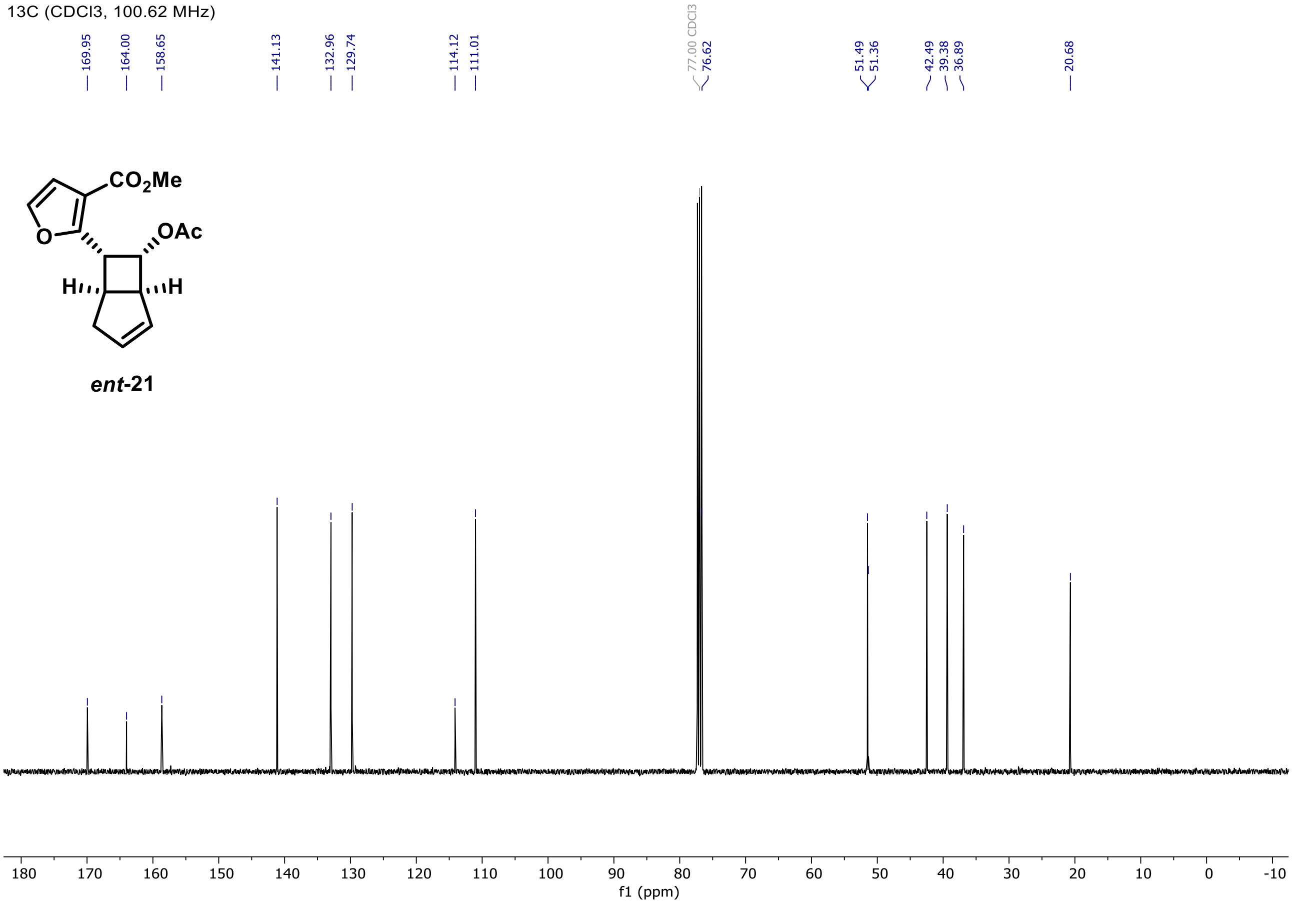

ent-21

1H (CDCl3, 400.12 MHz)

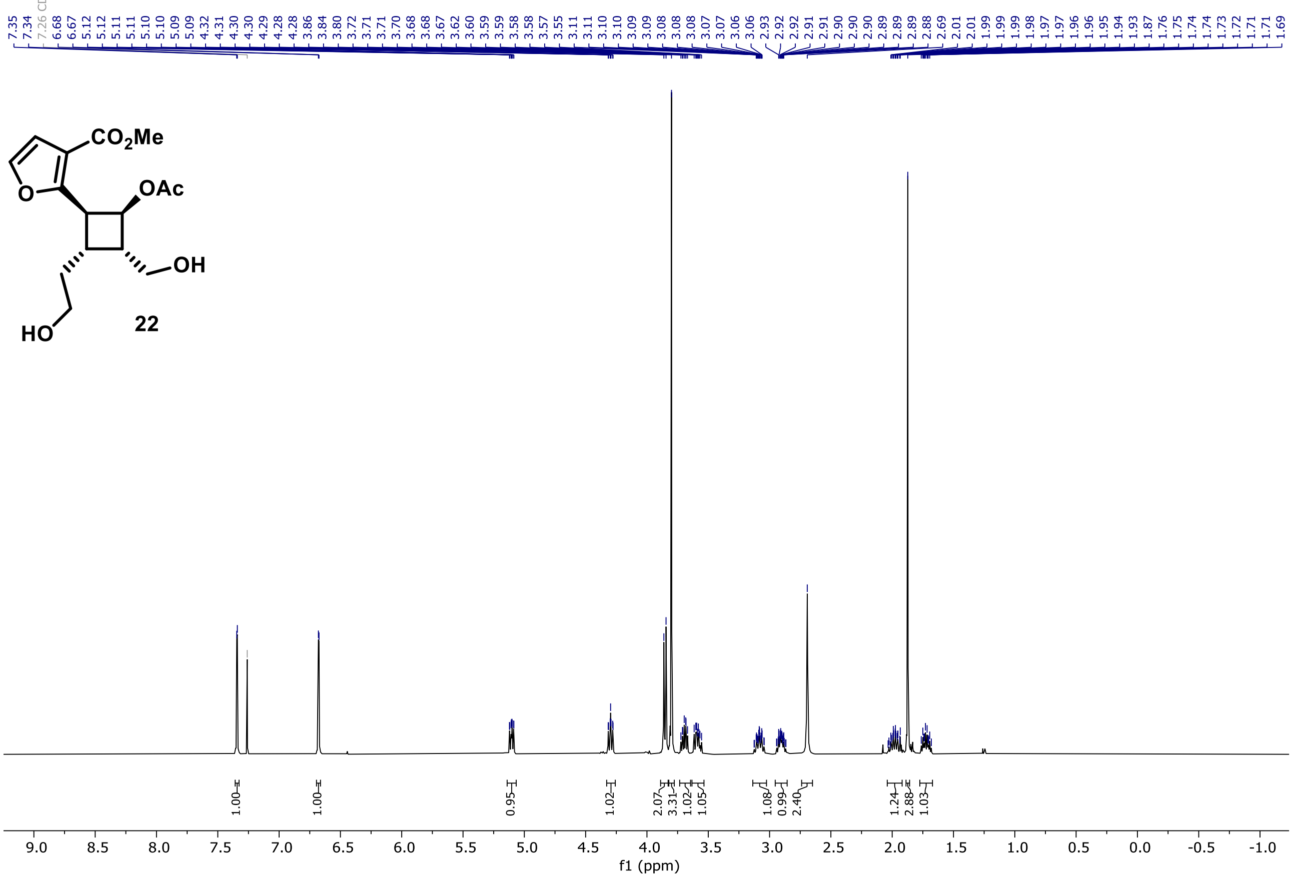

13C (CDCl3, 100.62 MHz)

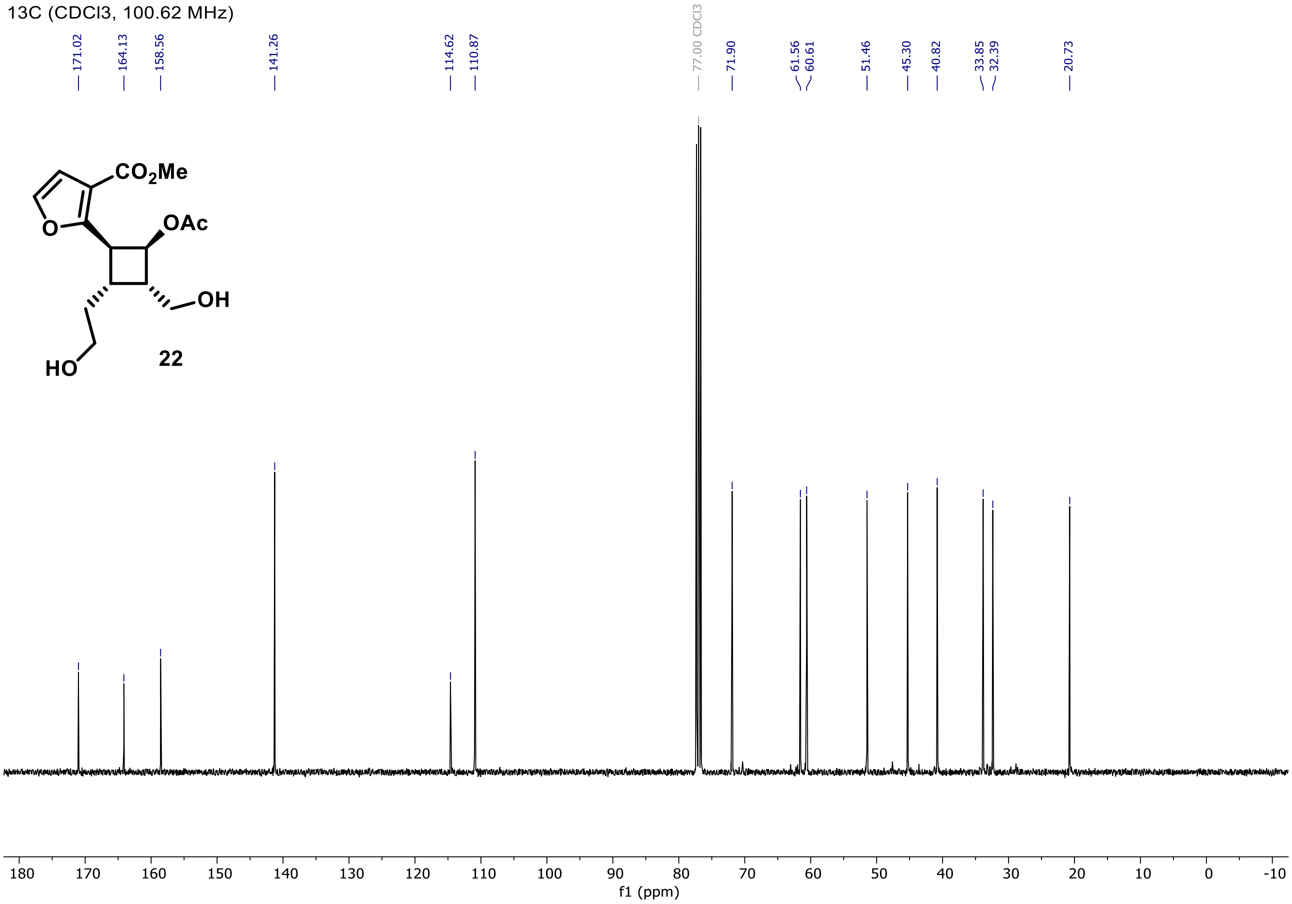

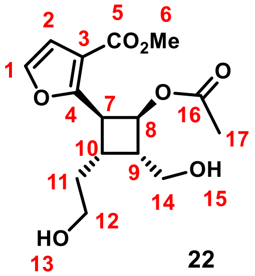

22

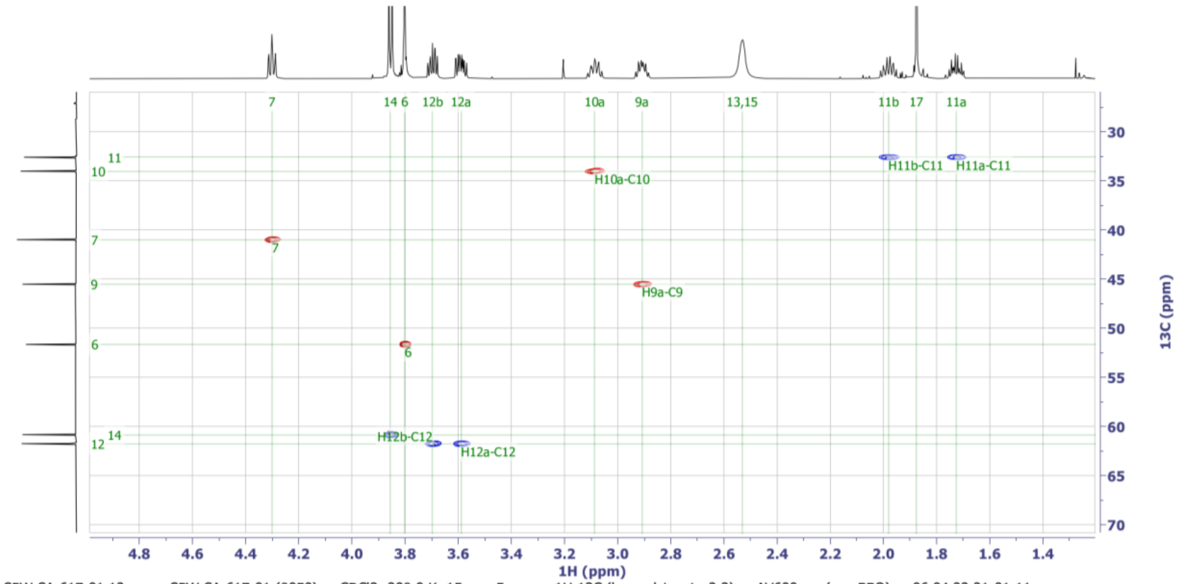

SPW-SA-617-01.12.ser — SPW-SA-617-01 (8059) — CDCl3; 298.0 K; 15 mg; 5 mm — 1H-13C (hsqcedetgpsisp2.3) — AV600neo (cryoBBO) — 06.04.22 21:01:11

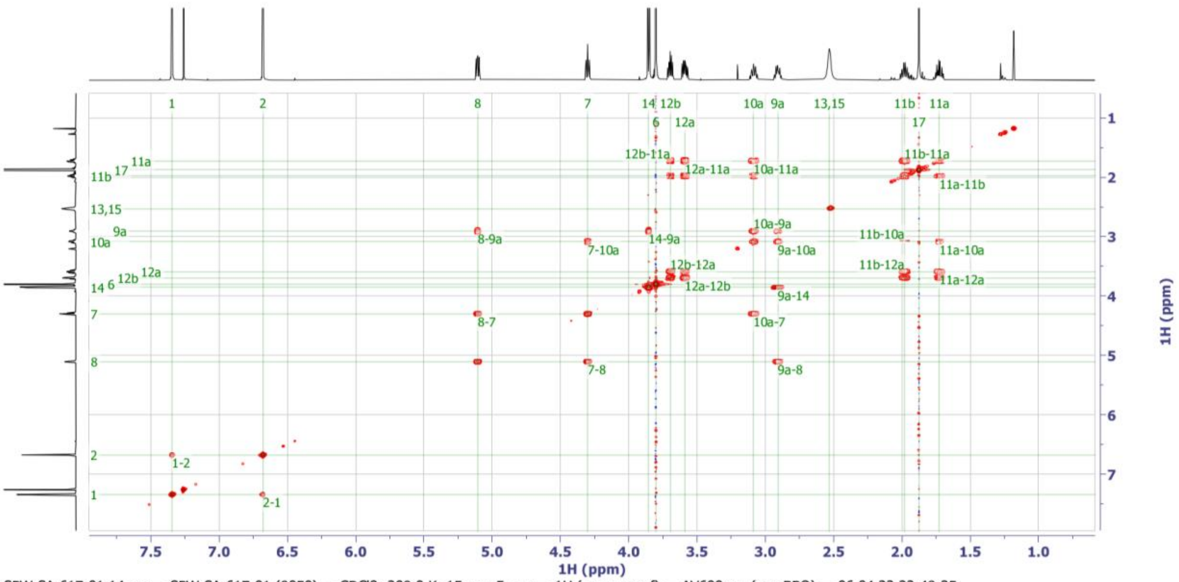

SPW-SA-617-01.14.ser — SPW-SA-617-01 (8059) — CDCl3; 298.0 K; 15 mg; 5 mm — 1H (cosygpppqf) — AV600neo (cryoBBO) — 06.04.22 22:48:35

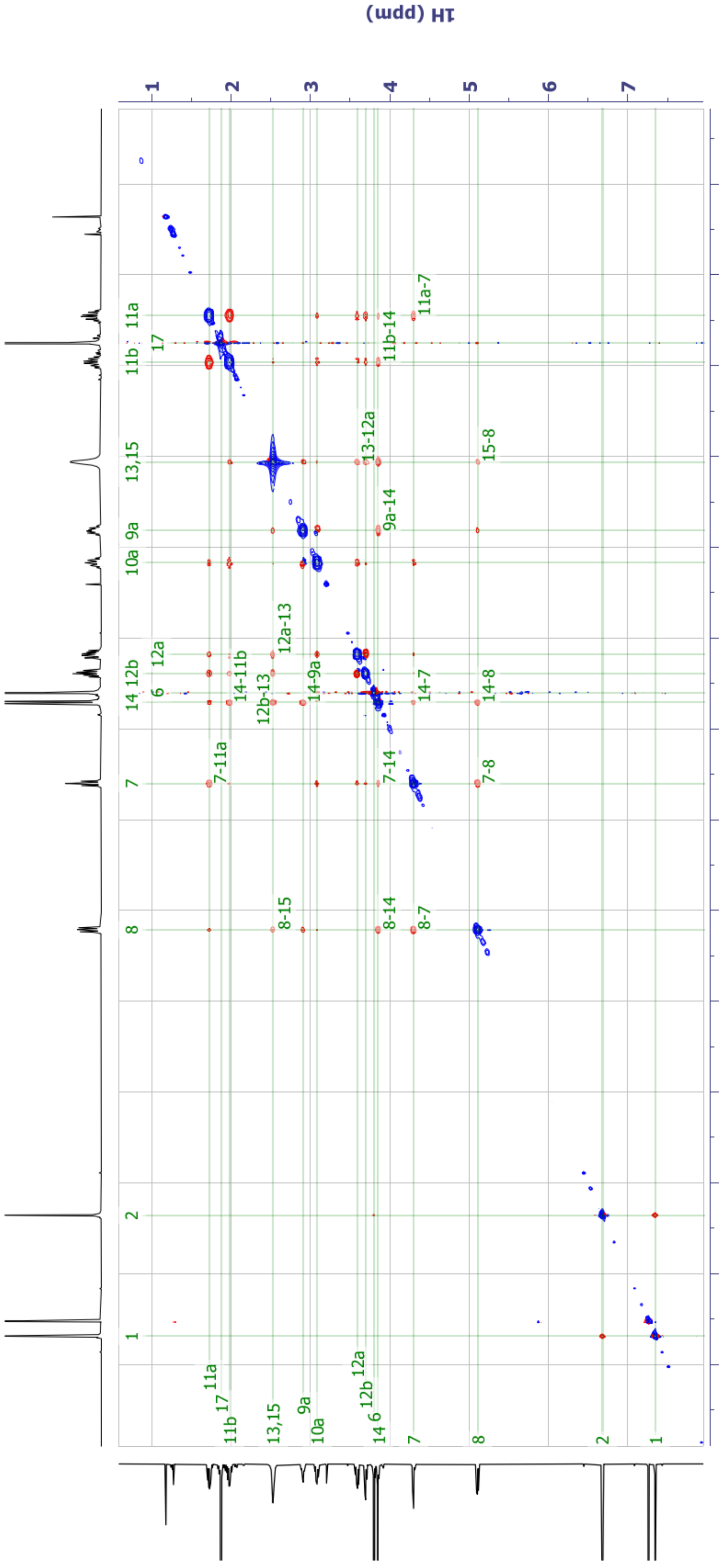

SPW-SA-617-01.15.ser — SPW-SA-617-01 (8059) — CDCl3; 298.0 K; 15 mg; 5 mm — 1H (noesygpphpp) — AV600neo (cryoBBO) — 06.04.22 22:39:45

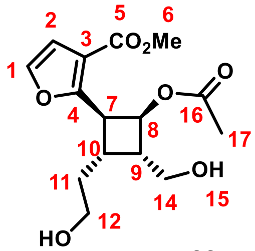

22

1H (CDCl3, 400.12 MHz)

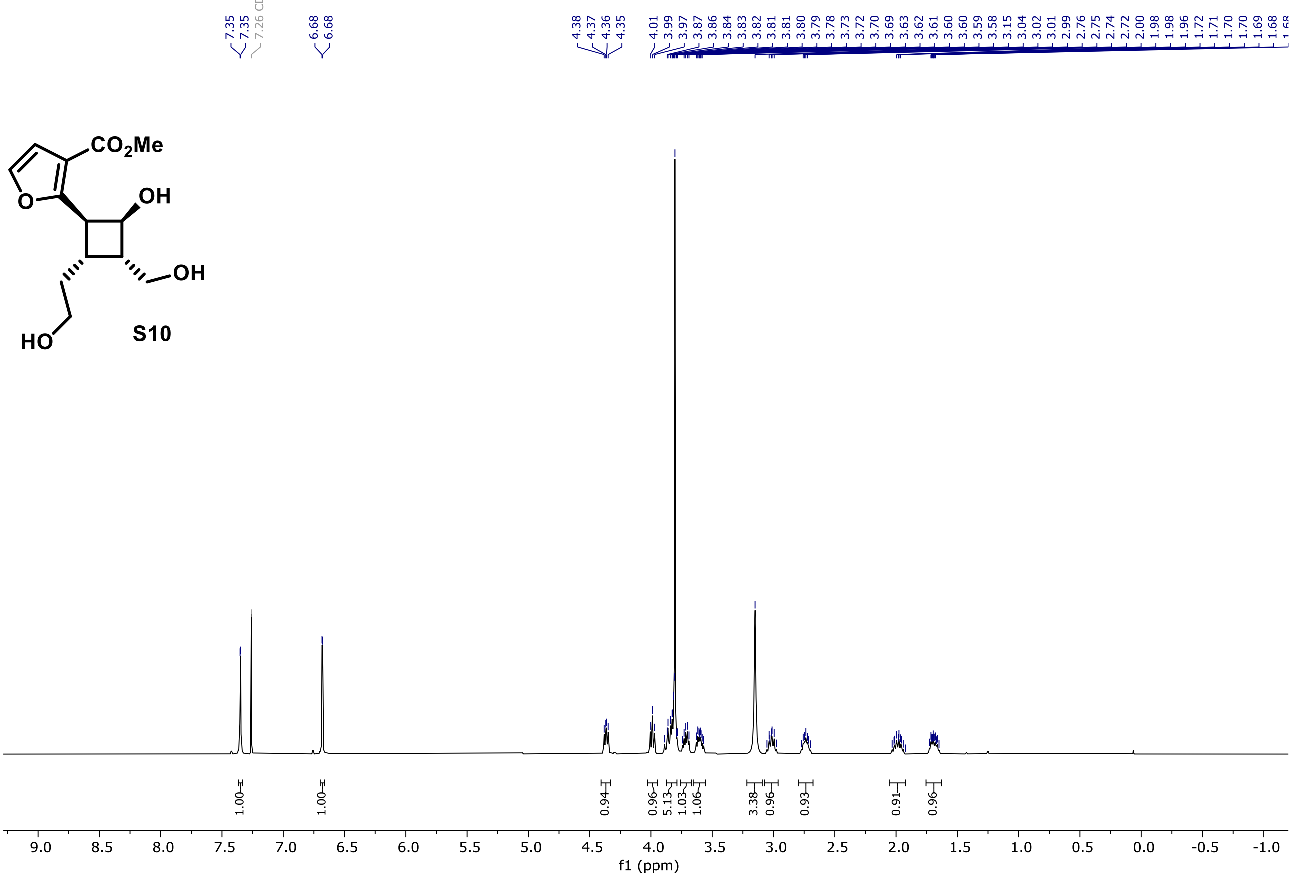

13C (CDCl3, 100.62 MHz)

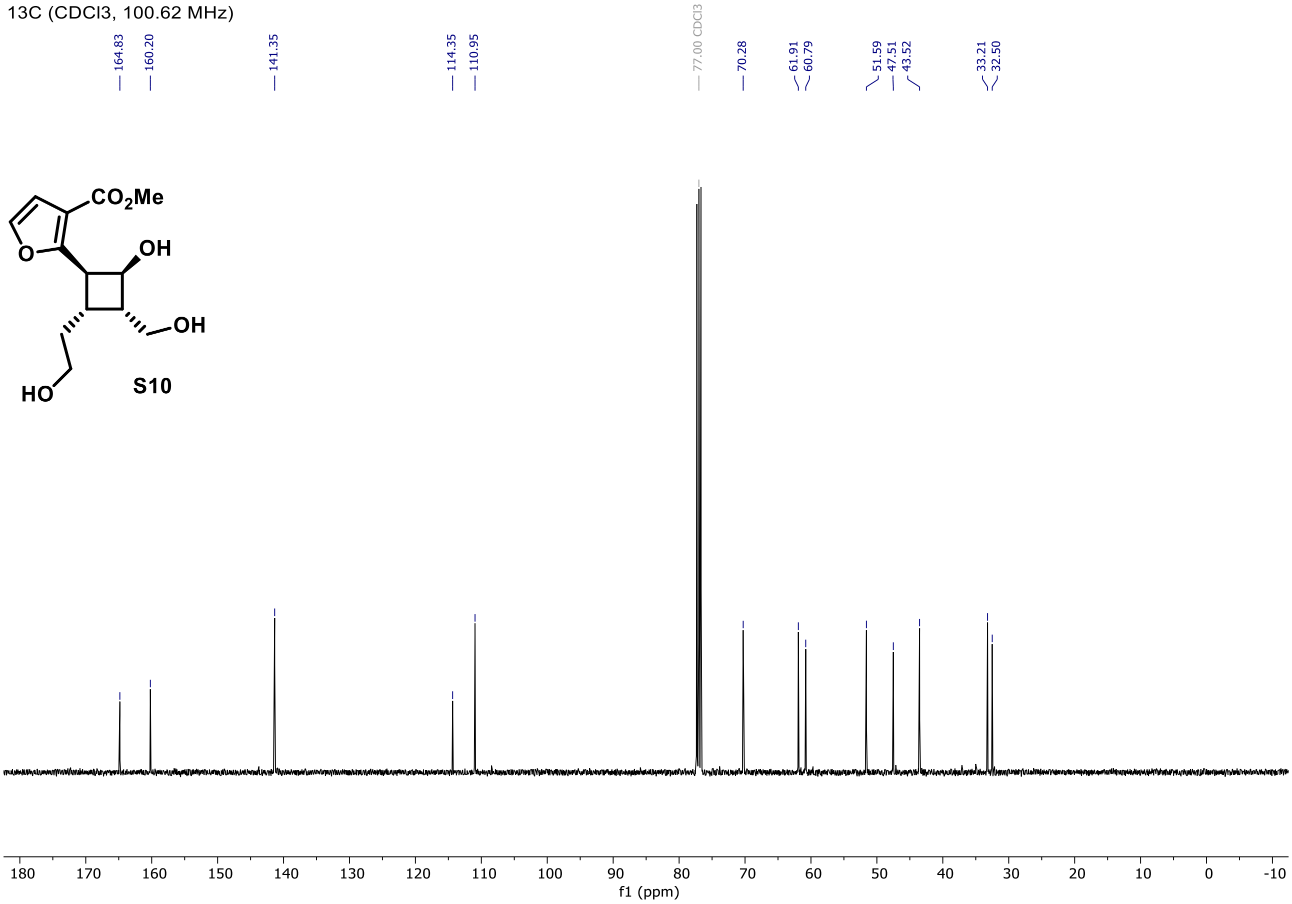

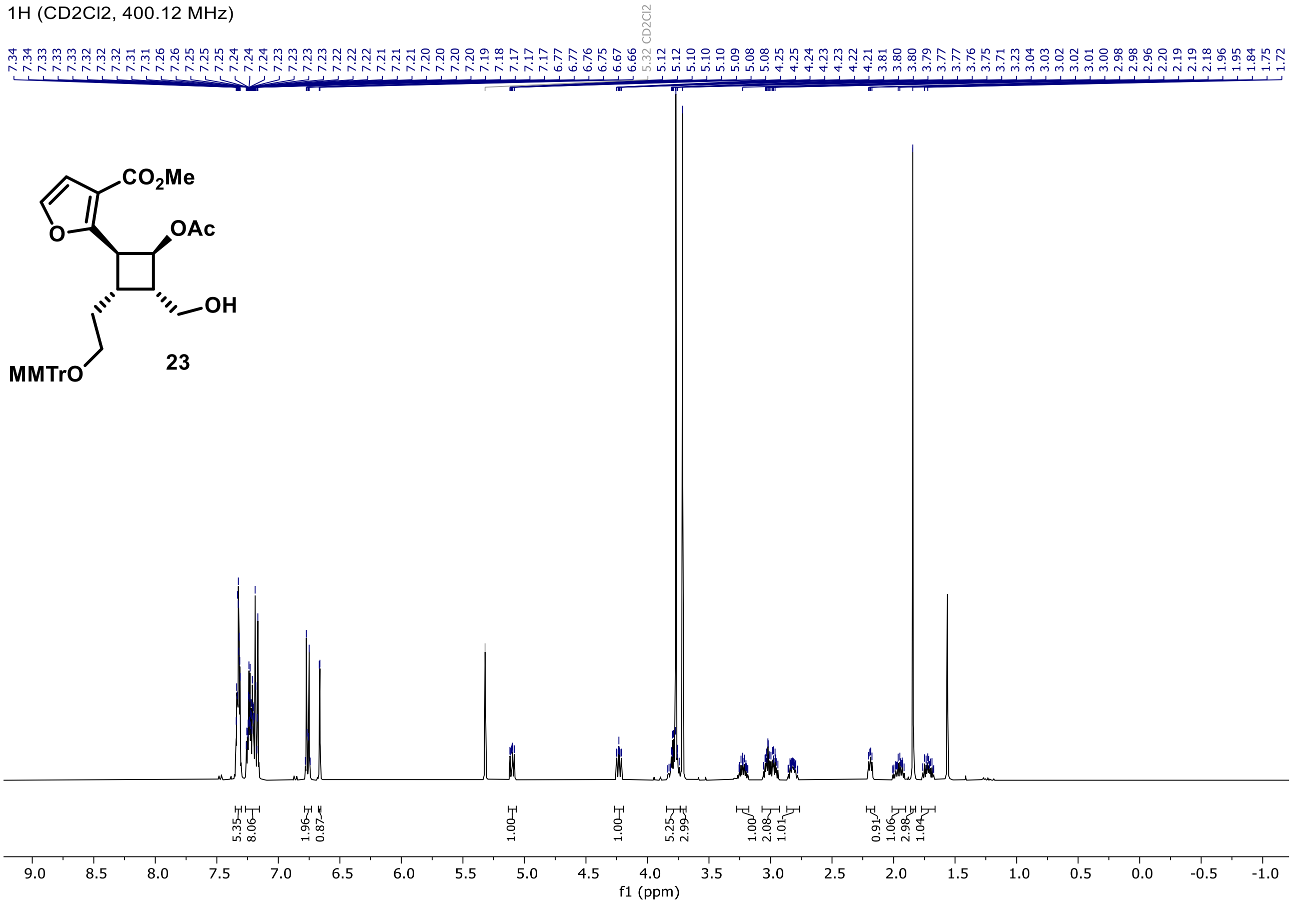
1H (CD2Cl2, 400.12 MHz)
7.34 7.34 7.33 7.33 7.33 7.32 7.32 7.32 7.31 7.31 7.26 7.26 7.25 7.25 7.25 7.24 7.24 7.24 7.24 7.23 7.23 7.23 7.22 7.22 7.22 7.21 7.21 7.20 7.20 7.20 7.19 7.18 7.17 7.17 7.17 6.77 6.77 6.76 6.75 6.67 6.66 5.32 CD2Cl2 5.12 5.12 5.10 5.10 5.10 5.09 5.08 5.08 4.25 4.25 4.24 4.23 4.23 4.22 4.21 3.81 3.80 3.80 3.79 3.77 3.77 3.76 3.75 3.71 3.23 3.04 3.03 3.02 3.02 3.01 3.00 2.98 2.98 2.96 2.20 2.19 2.18 1.96 1.95 1.84 1.75 1.72
CO2Me
OAc
O
OH
MMTrO
23
5.35 8.06 1.96 0.87 1.00 1.00 5.25 2.99 1.00 2.08 1.01 0.91 1.06 2.98 1.04
9.0 8.5 8.0 7.5 7.0 6.5 6.0 5.5 5.0 4.5 4.0 3.5 3.0 2.5 2.0 1.5 1.0 0.5 0.0 -0.5 -1.0
f1 (ppm)

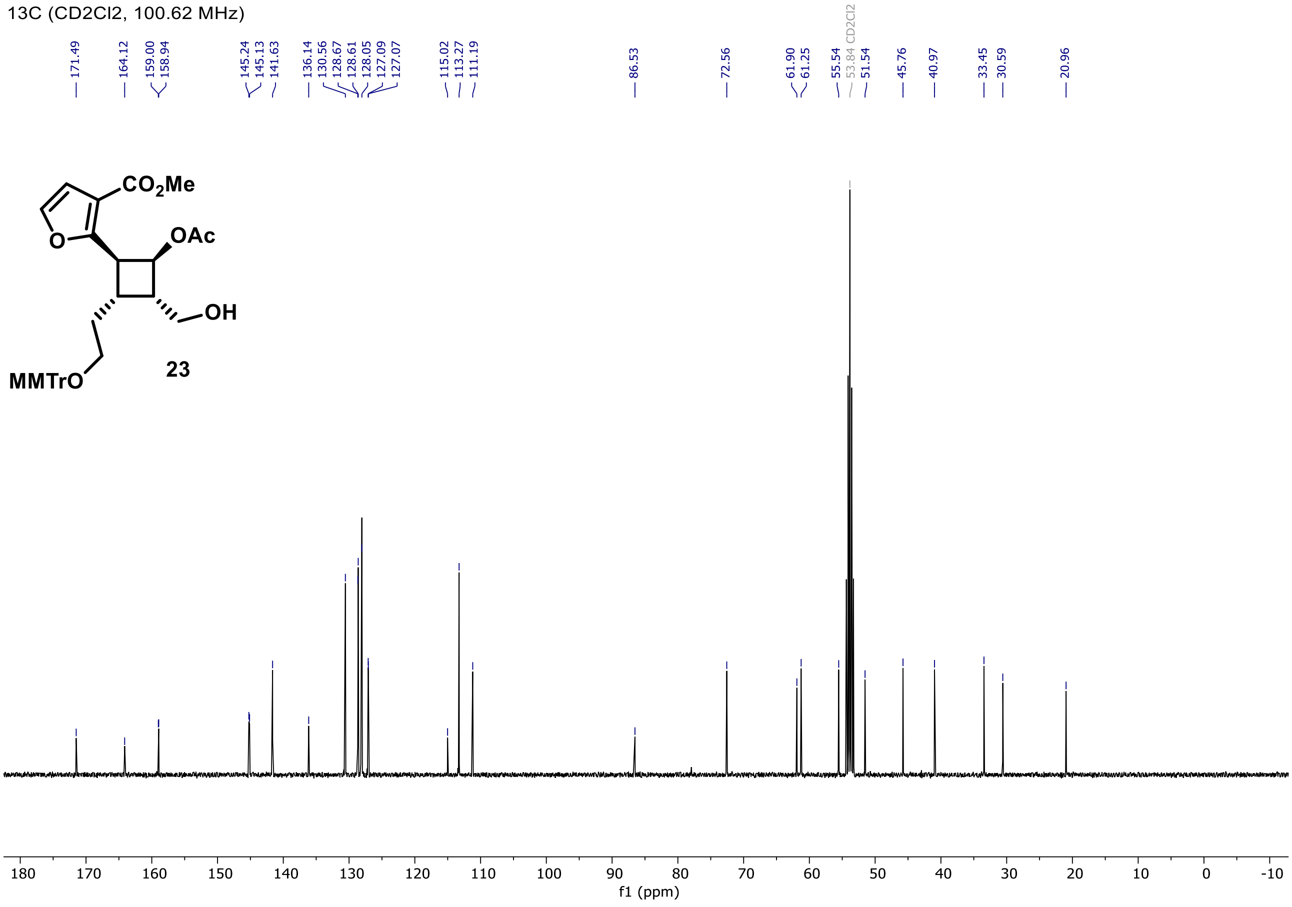
13C (CD2Cl2, 100.62 MHz)
171.49 164.12 159.00 158.94 145.24 145.13 141.63 136.14 130.56 128.67 128.61 128.05 127.09 127.07 115.02 113.27 111.19 86.53 72.56 61.90 61.25 55.54 53.84 CD2Cl2 51.54 45.76 40.97 33.45 30.59 20.96
CO2Me
OAc
O
OH
MMTrO
23
180 170 160 150 140 130 120 110 100 90 80 70 60 50 40 30 20 10 0 -10
f1 (ppm)

1H (CD2Cl2, 400.12 MHz)

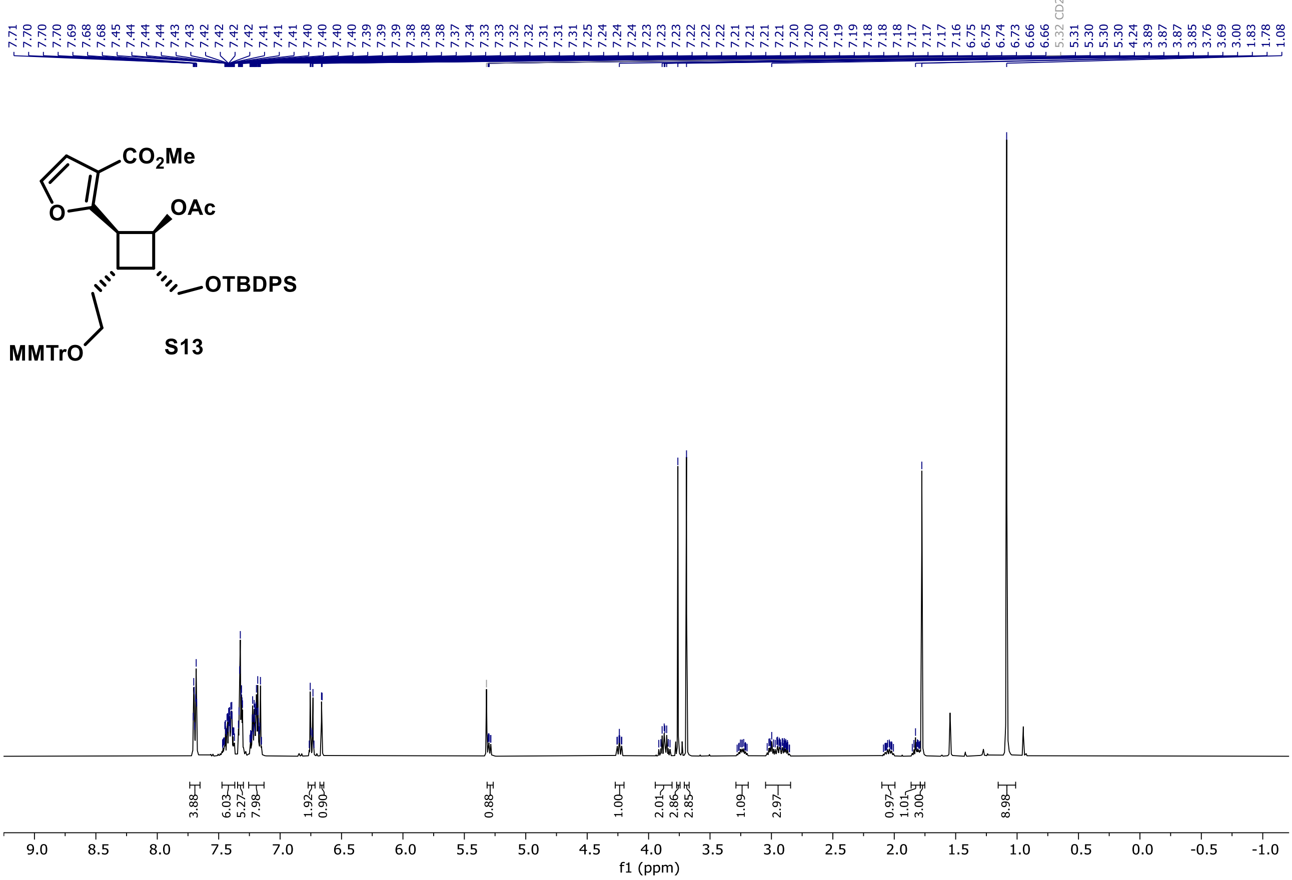

13C (CD2Cl2, 100.62 MHz)

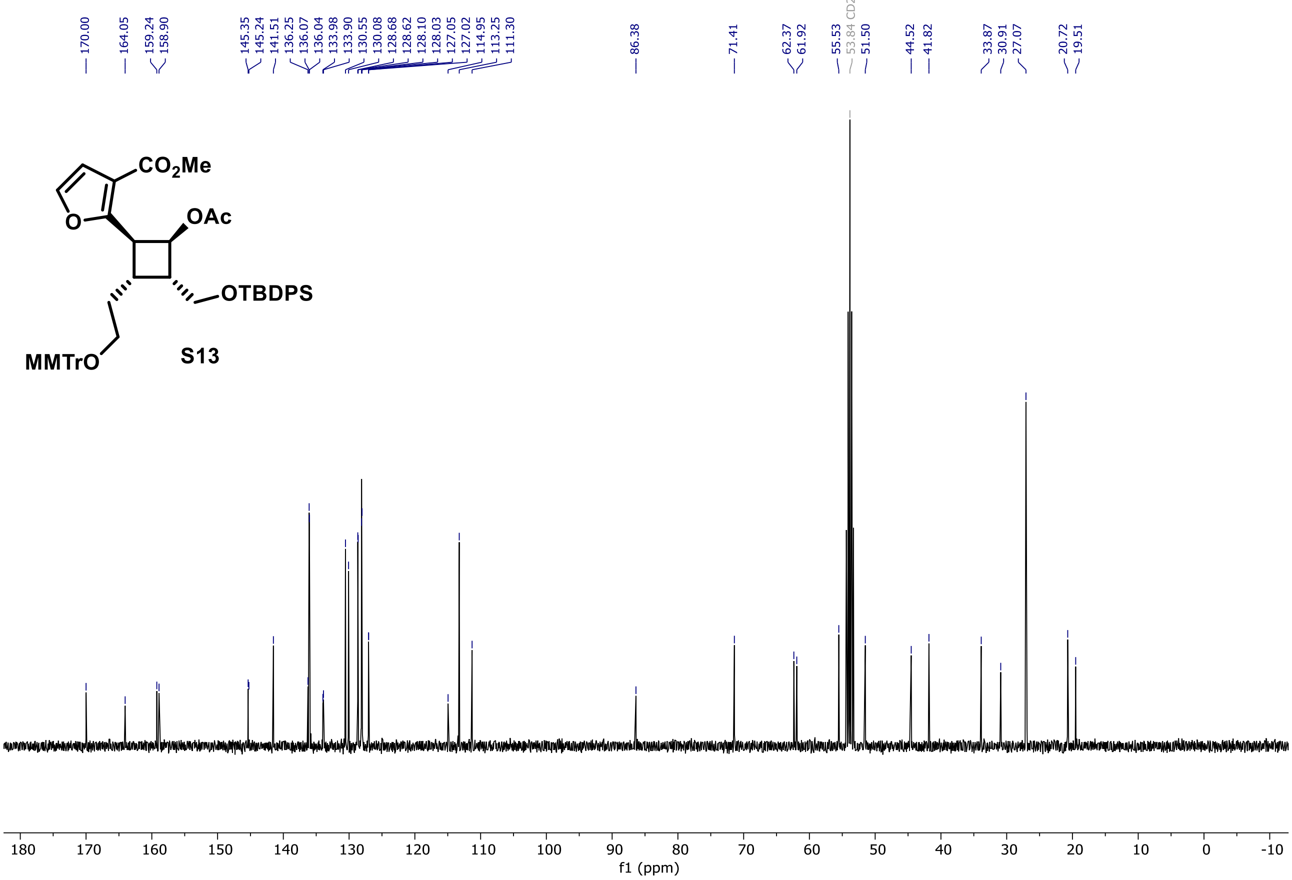

1H (CDCl3, 400.12 MHz)

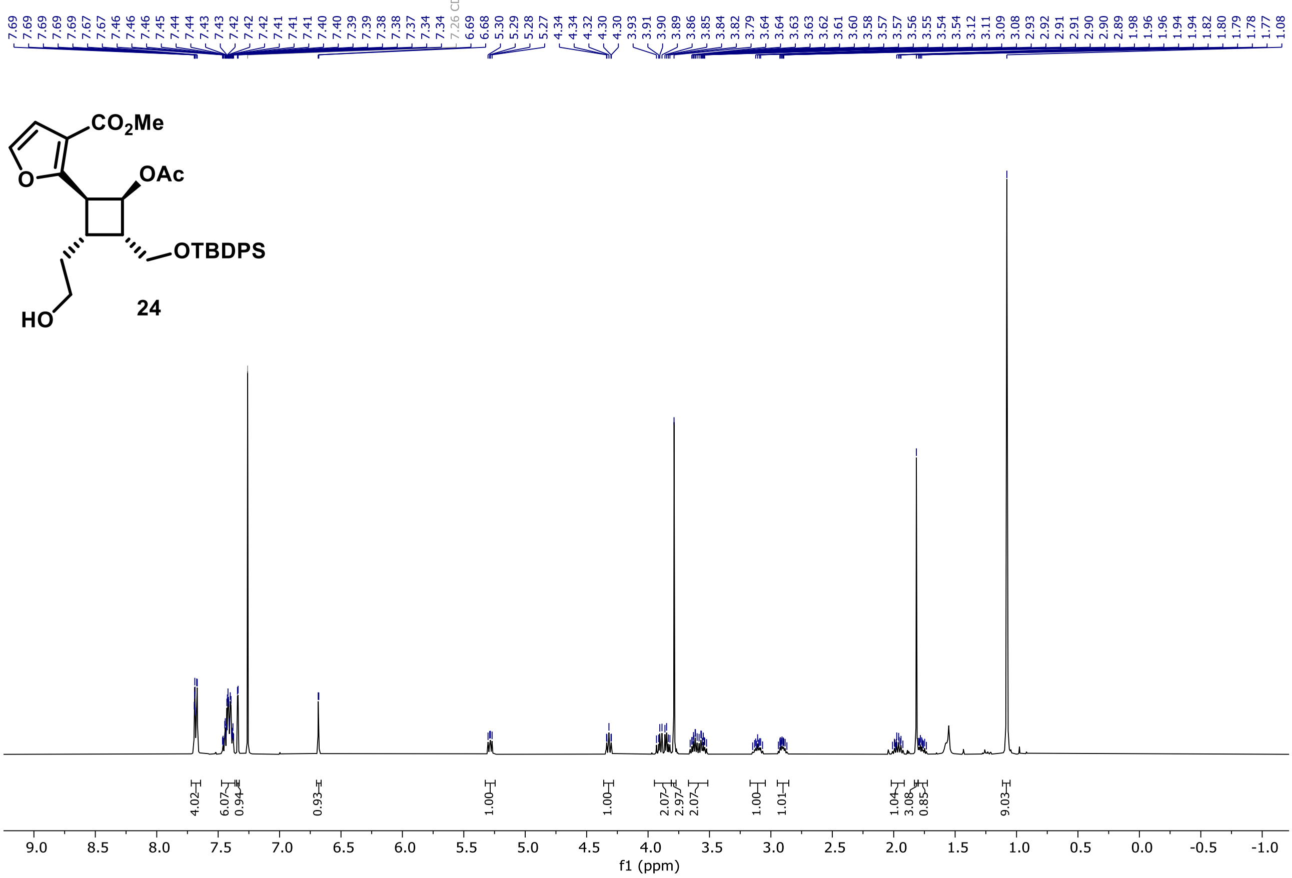

13C (CDCl3, 100.62 MHz)

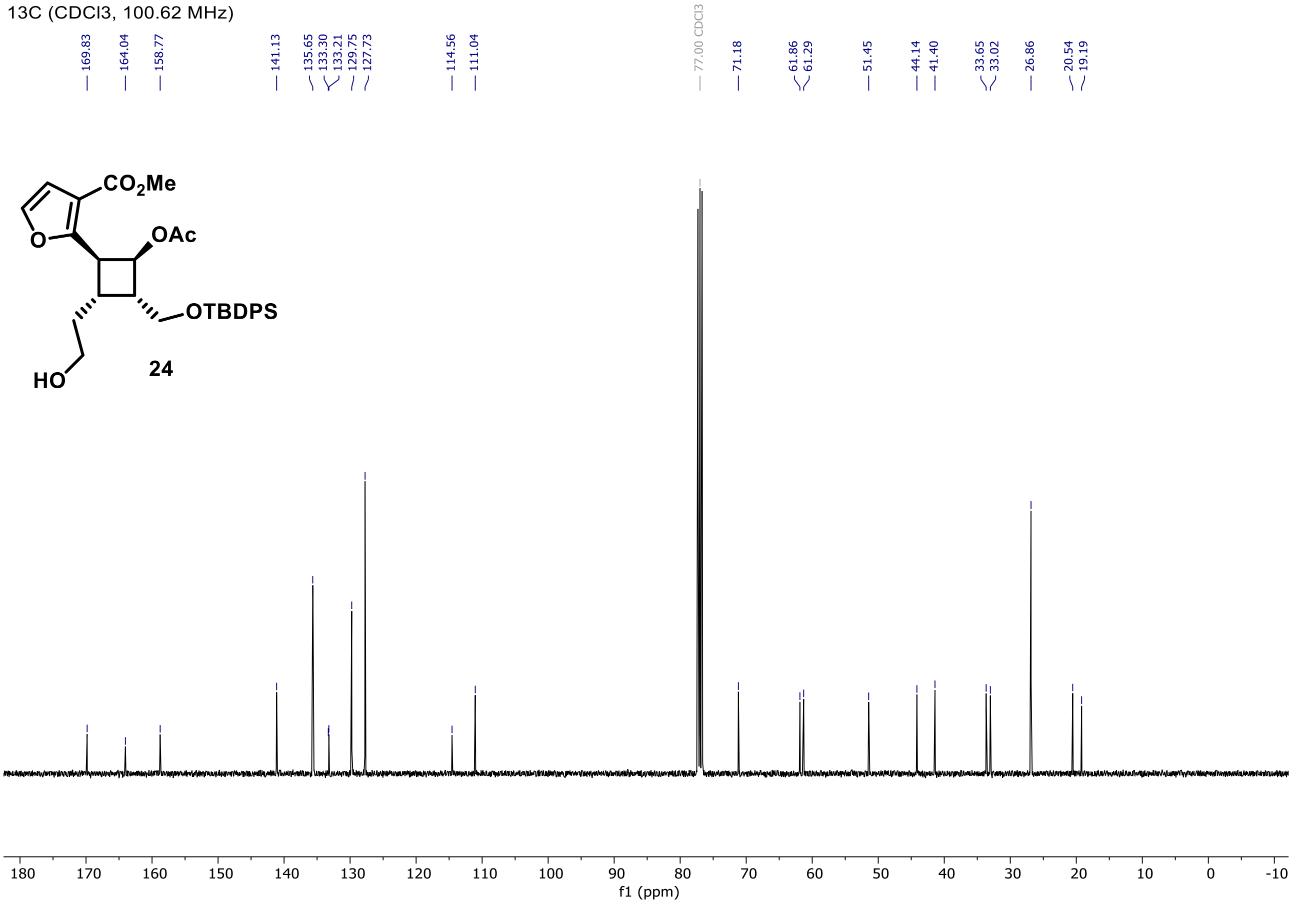

1H (CD2Cl2, 400.12 MHz)

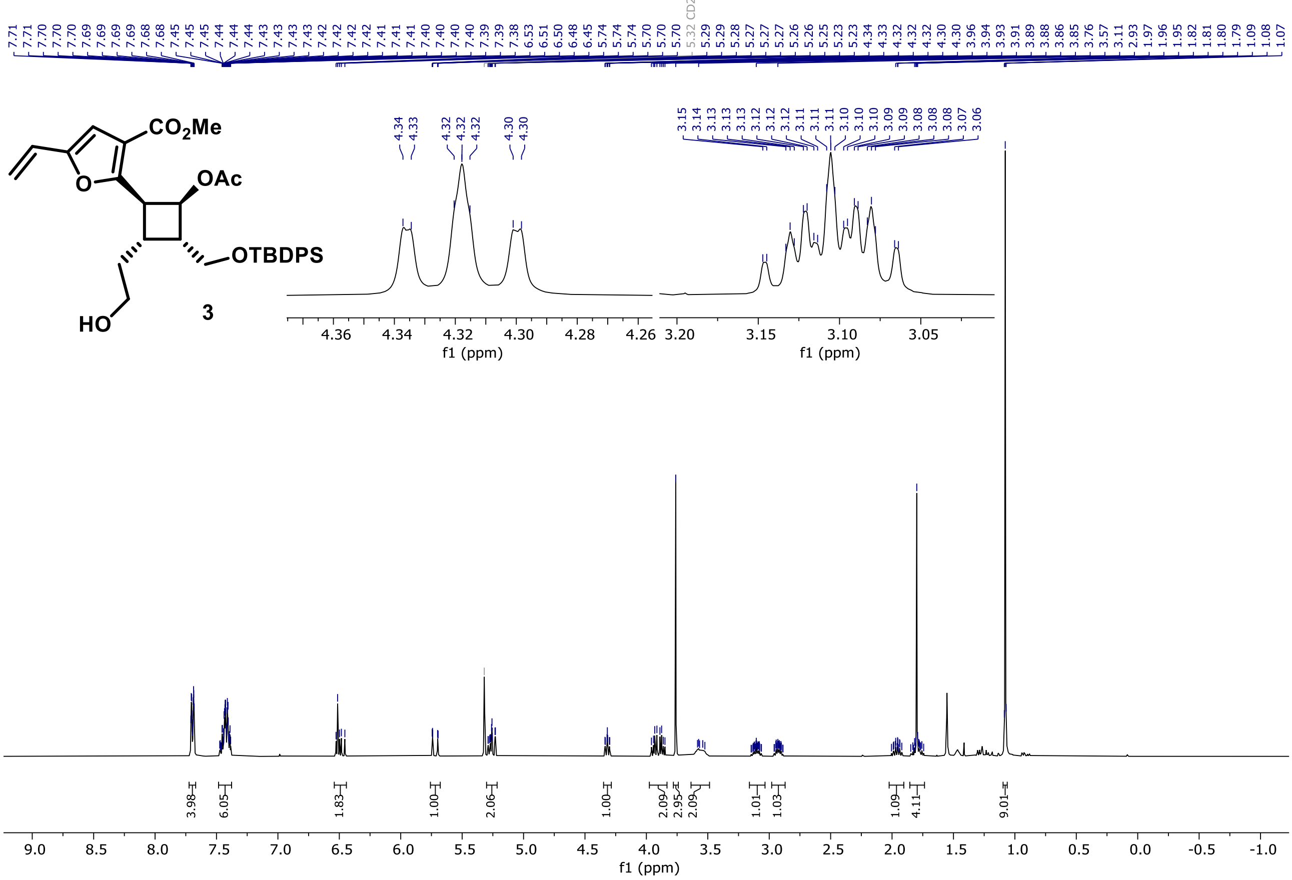

13C (CD2Cl2, 100.62 MHz)

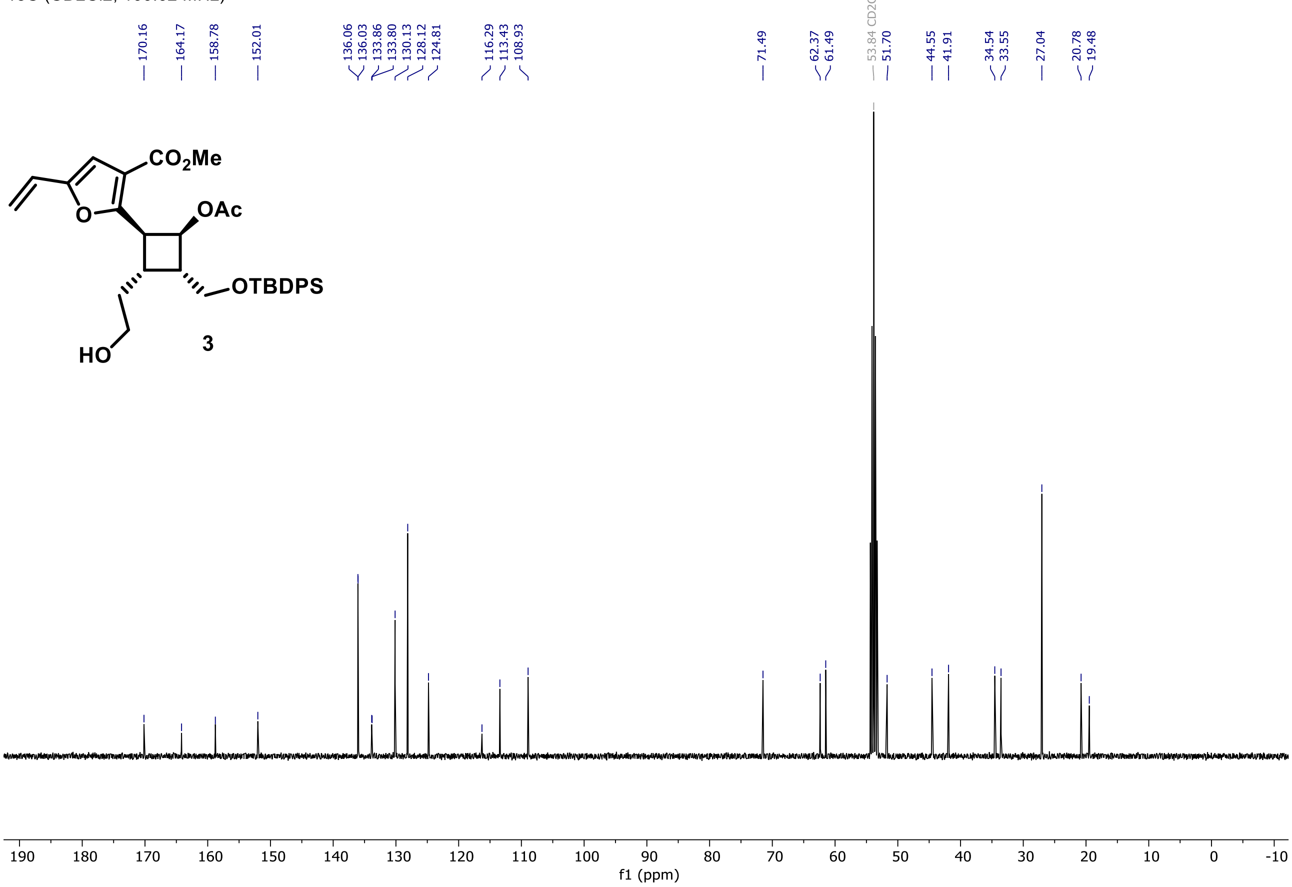

1H (CD2Cl2, 400.12 MHz)

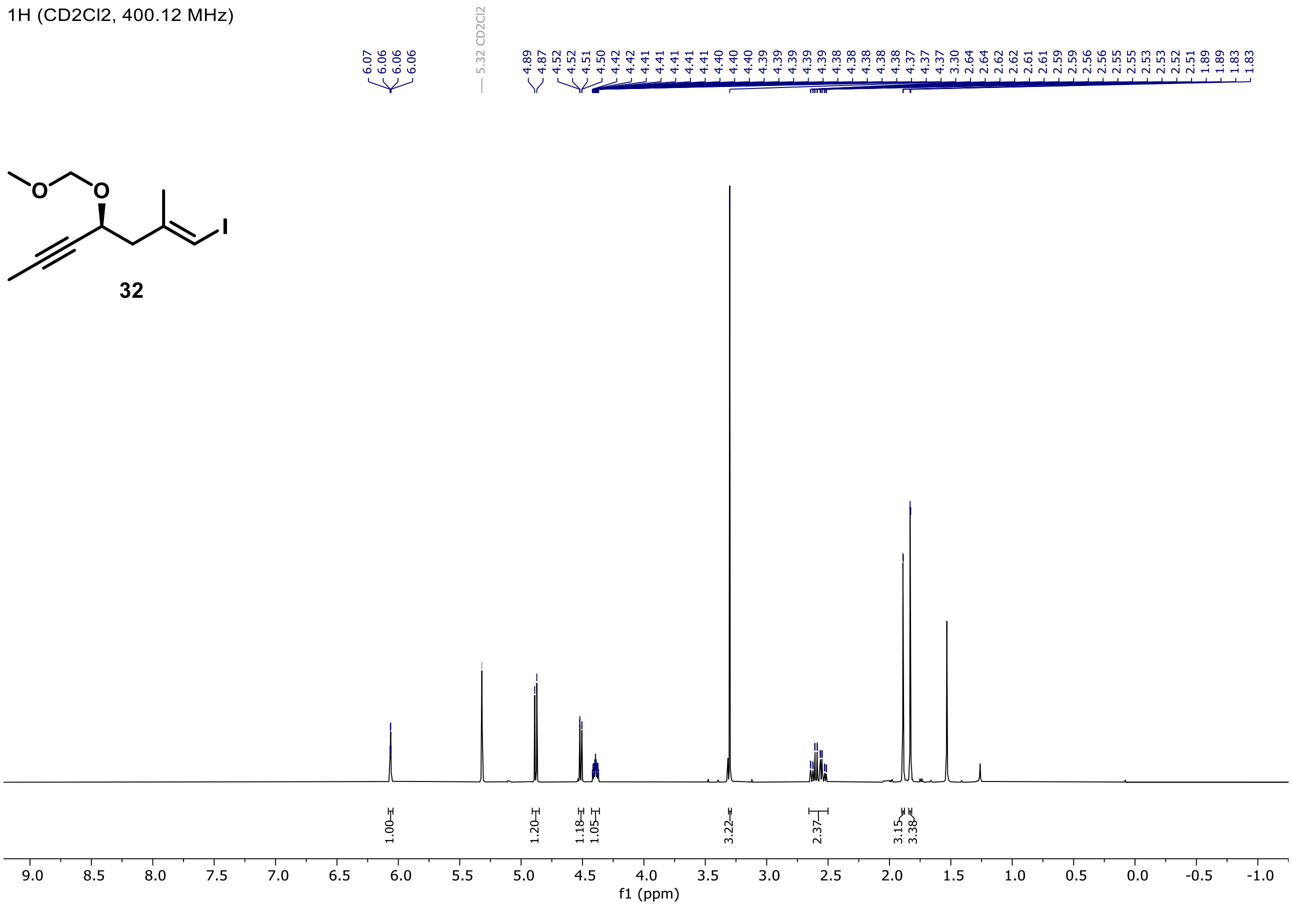

13C (CD2Cl2, 100.62 MHz)

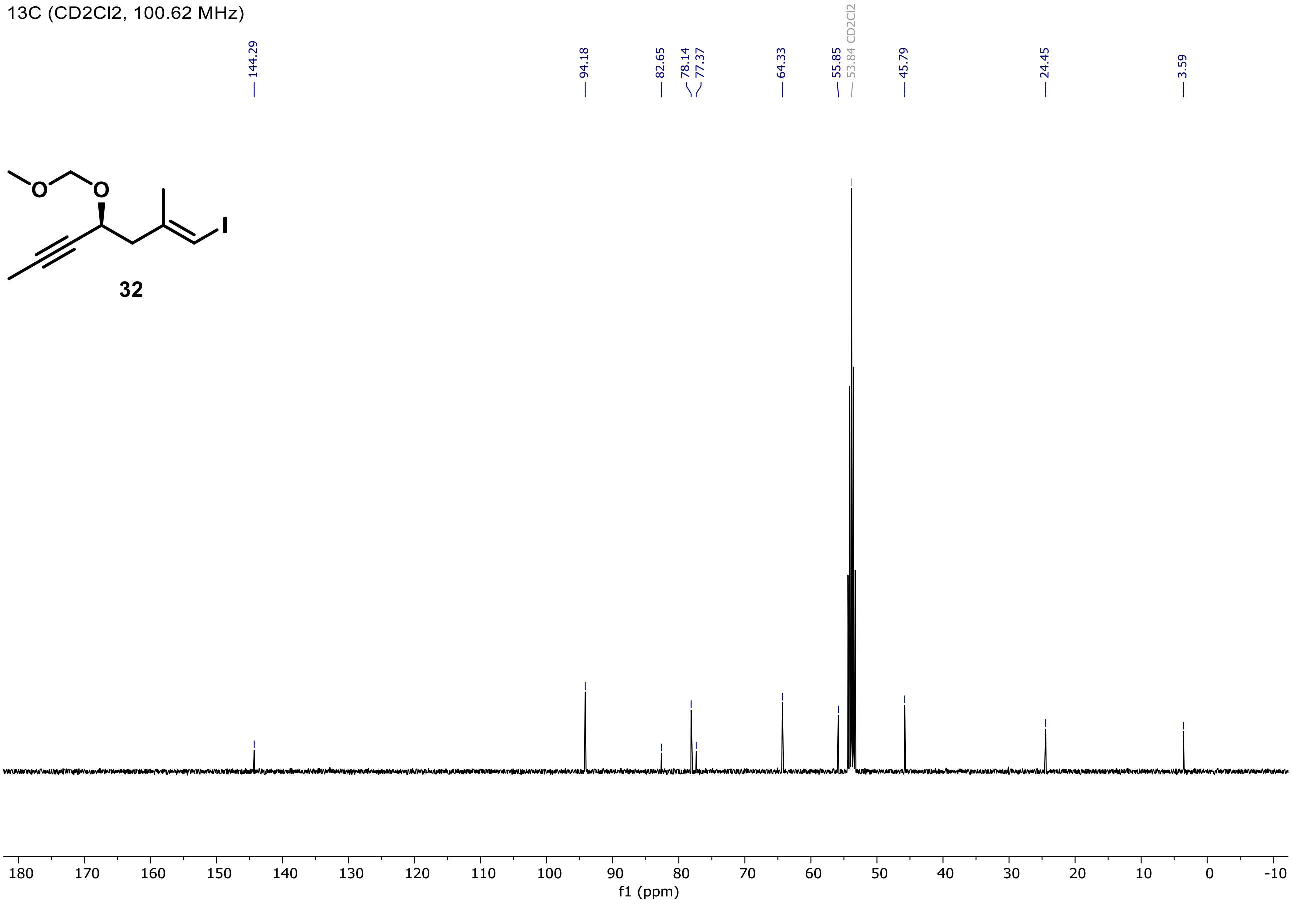

1H (CD2Cl2, 400.12 MHz)

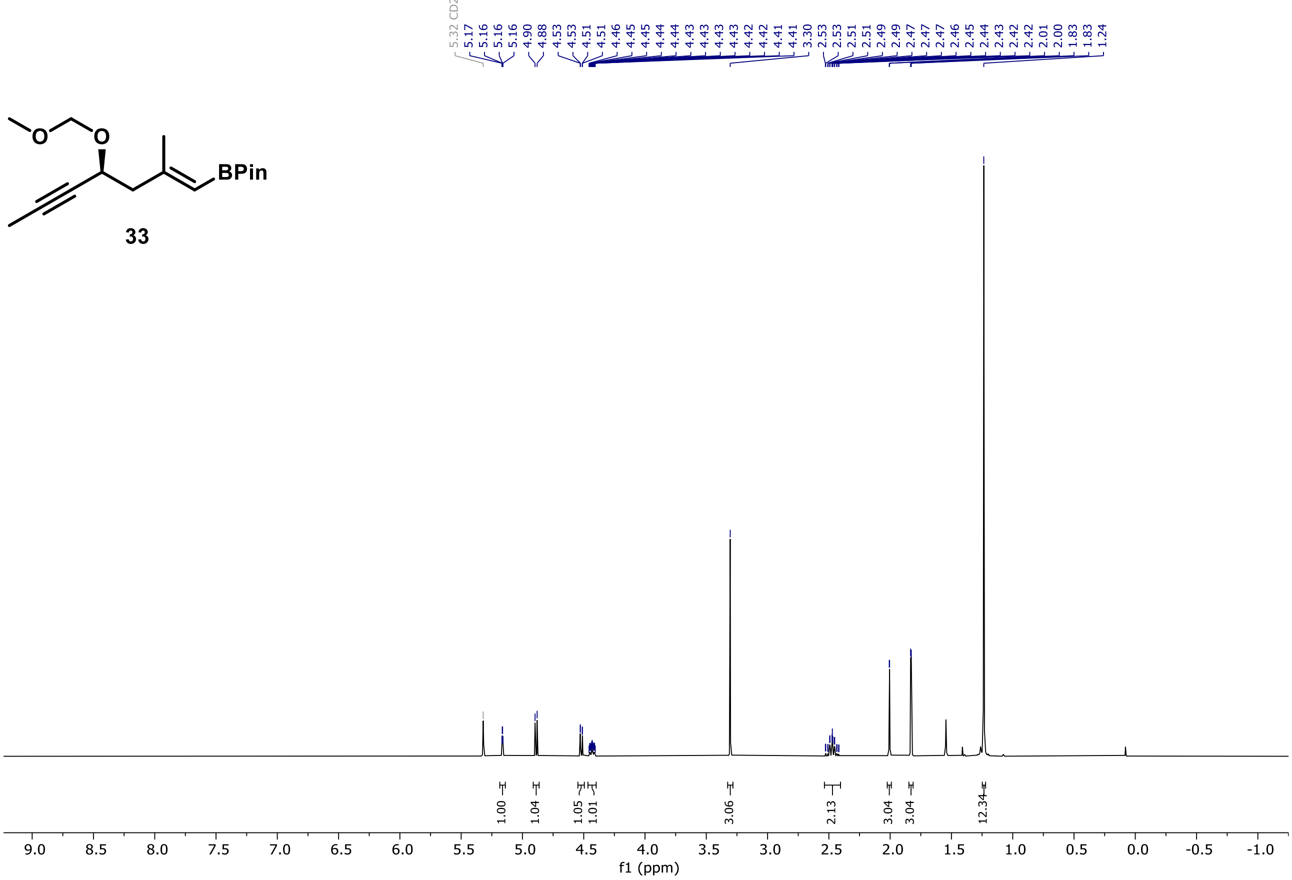

13C (CD2Cl2, 100.62 MHz)

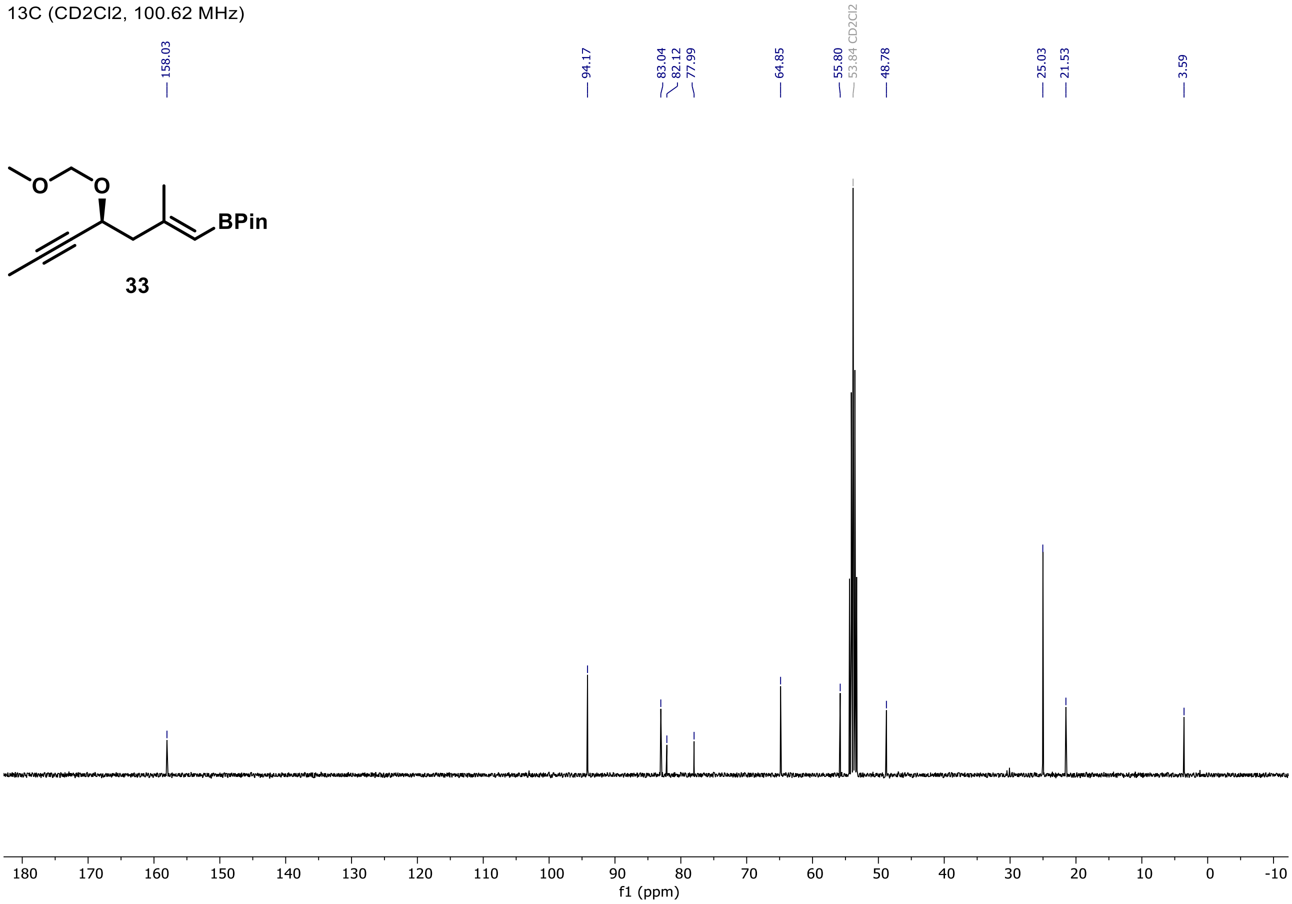

1H (CD2Cl2, 400.12 MHz)

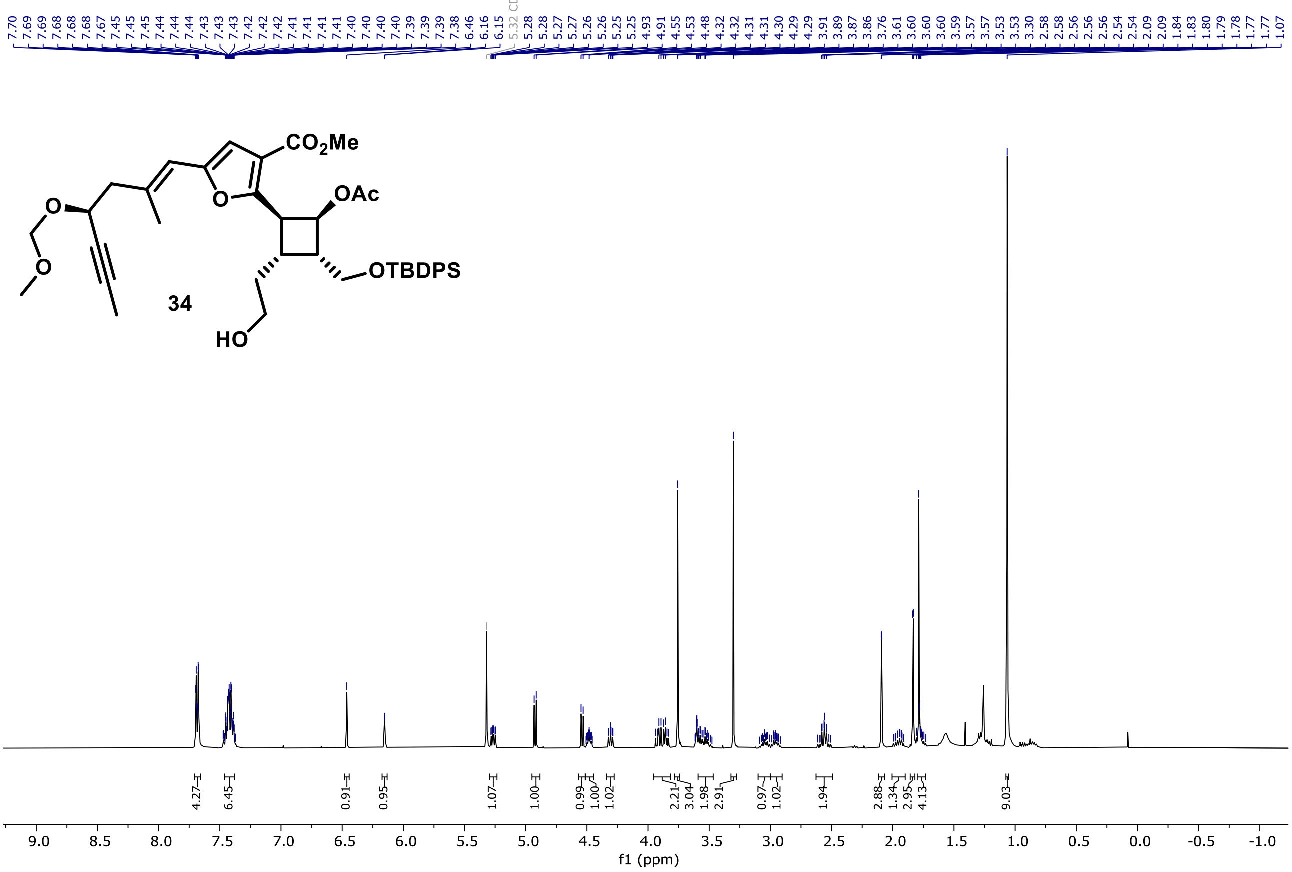

13C (CD2Cl2, 100.62 MHz)

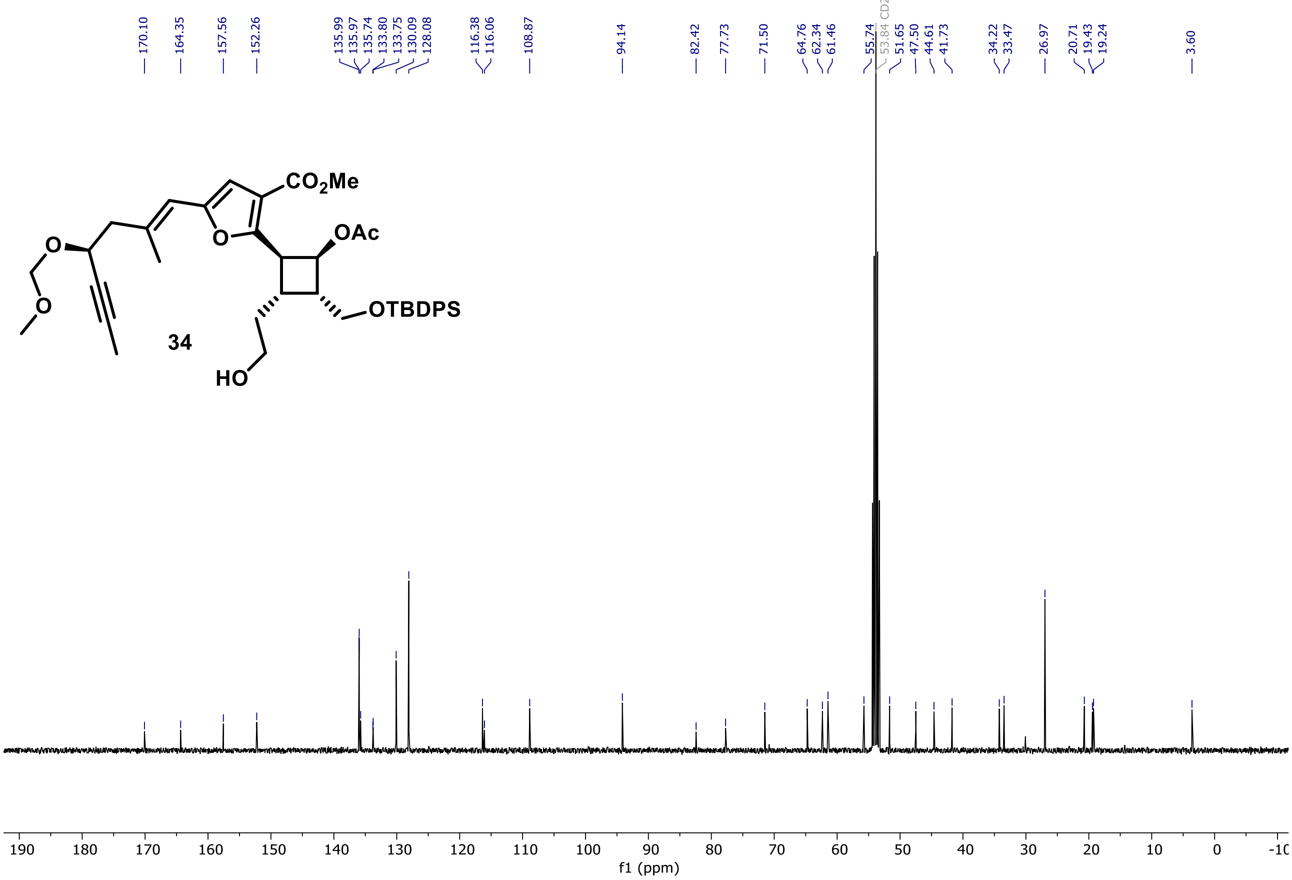

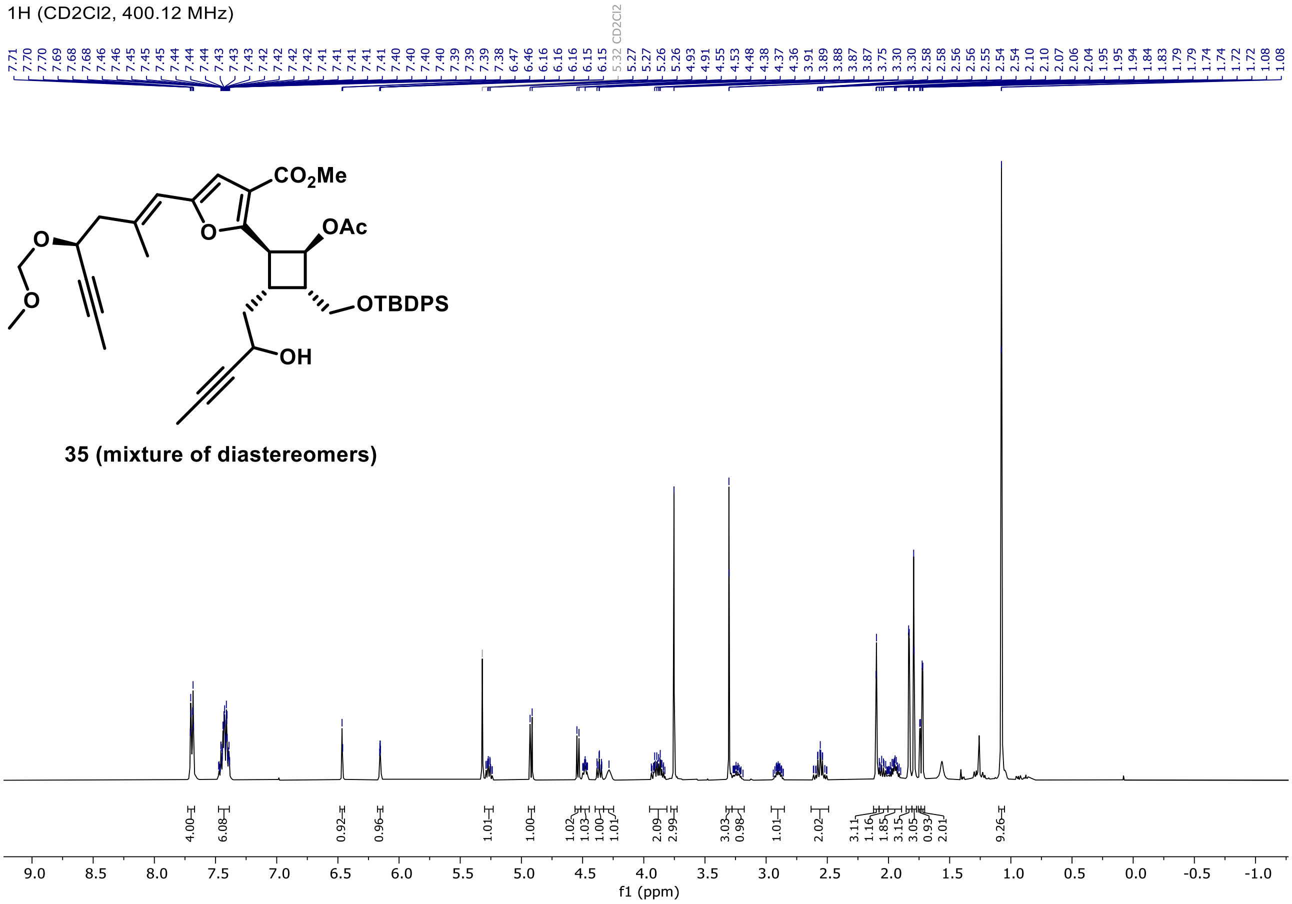

1H (CD2Cl2, 400.12 MHz)
7.71 7.70 7.70 7.69 7.68 7.46 7.46 7.45 7.45 7.45 7.44 7.44 7.44 7.43 7.43 7.43 7.42 7.42 7.42 7.42 7.41 7.41 7.41 7.41 7.40 7.40 7.40 7.40 7.39 7.39 7.39 7.38 6.47 6.46 6.16 6.16 6.16 6.15 6.15 5.32 CD2Cl2 5.27 5.27 5.26 5.26 4.93 4.91 4.55 4.53 4.48 4.38 4.37 4.36 3.91 3.89 3.88 3.87 3.87 3.75 3.30 3.30 2.58 2.58 2.56 2.56 2.55 2.54 2.54 2.10 2.10 2.07 2.06 2.04 1.95 1.95 1.94 1.84 1.83 1.79 1.79 1.74 1.74 1.72 1.08 1.08
CO2Me
OAc
OTBDPS
OH
O
35 (mixture of diastereomers)
4.00 6.08 0.92 0.96 1.01 1.00 1.02 1.03 1.00 1.01 2.09 2.99 3.03 0.98 1.01 2.02 3.11 1.16 1.85 3.15 3.05 0.93 2.01 9.26
9.0 8.5 8.0 7.5 7.0 6.5 6.0 5.5 5.0 4.5 4.0 3.5 3.0 2.5 2.0 1.5 1.0 0.5 0.0 -0.5 -1.0
f1 (ppm)

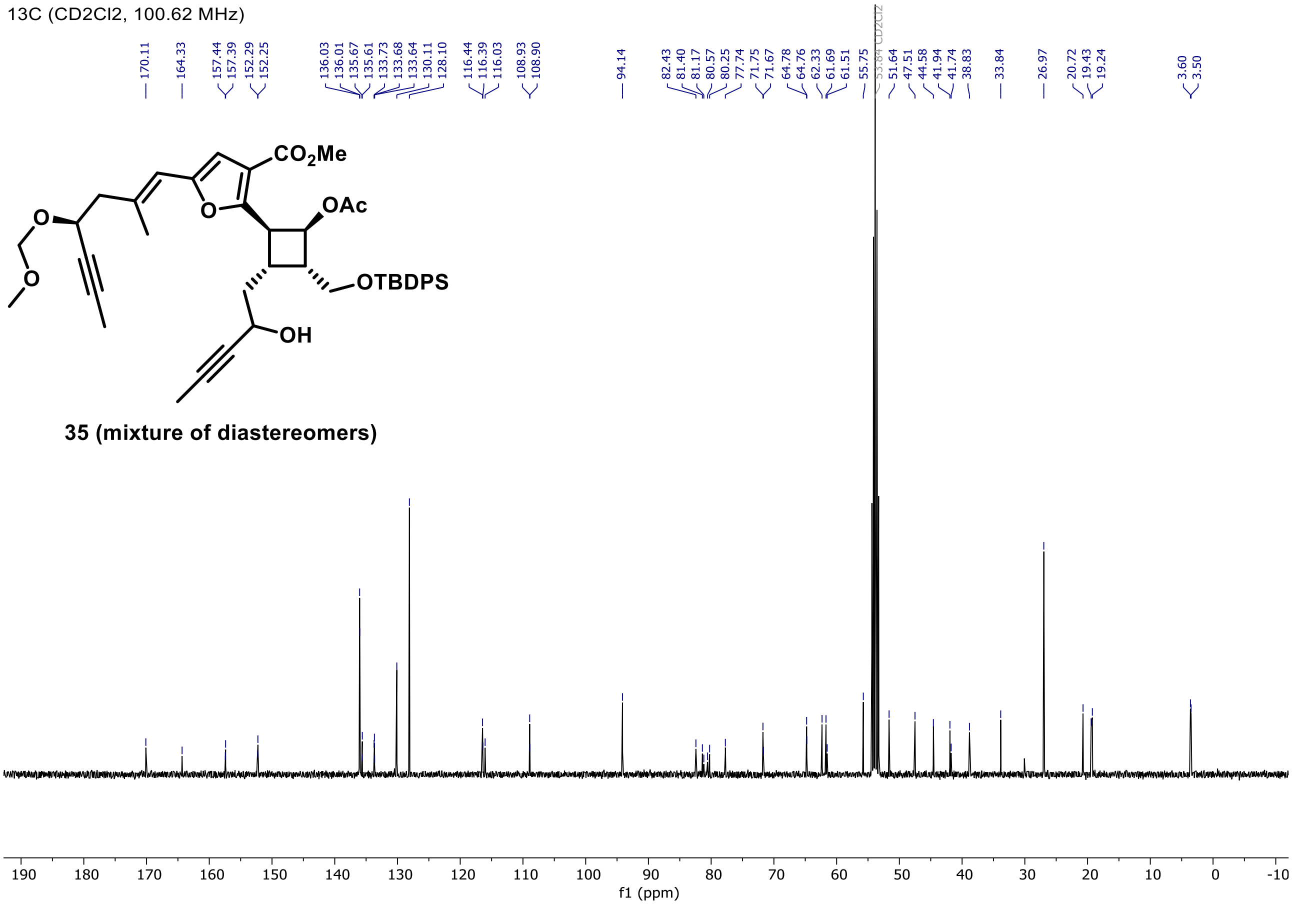

13C (CD2Cl2, 100.62 MHz)
170.11 164.33 157.44 157.39 152.29 152.25 136.03 136.01 135.67 135.61 133.73 133.68 133.64 130.11 128.10 116.44 116.39 116.03 108.93 108.90 94.14 82.43 81.40 81.17 80.57 80.25 77.74 71.75 71.67 64.78 64.76 62.33 61.69 61.51 55.75 53.84 CD2Cl2 51.64 47.51 44.58 41.94 41.74 38.83 33.84 26.97 20.72 19.43 19.24 3.60 3.50
CO2Me
OAc
OTBDPS
OH
O
35 (mixture of diastereomers)
190 180 170 160 150 140 130 120 110 100 90 80 70 60 50 40 30 20 10 0 -10
f1 (ppm)

References

[1] A. J. Varni, A. Fortney, M. A. Baker, J. C. Worch, Y. Qiu, D. Yaron, S. Bernhard, K. J. T. Noonan, T. Kowalewski, *J. Am. Chem. Soc.* **2019**, *141*, 8858–8867.

[2] W. J. Ramsay, N. A. W. Bell, Y. Qing, H. Bayley, *J. Am. Chem. Soc.* **2018**, *140*, 17538–17546.

[3] M. Penner, V. Rauniyar, L. T. Kaspar, D. G. Hall, *J. Am. Chem. Soc.* **2009**, *131*, 14216–14217.

[4] S. Schaubach, K. Gebauer, F. Ungeheuer, L. Hoffmeister, M. K. Ilg, C. Wirtz, A. Fürstner, *Chem. - A Eur. J.* **2016**, *22*, 8494–8507.

[5] J. Hillenbrand, M. Leutzsch, E. Yiannakas, C. Gordon, C. Wille, N. Nöthling, C. Copéret, A. Fürstner, *J. Am. Chem. Soc*. **2020**, *142*, 11279–11294.
